# Prion-like low complexity regions enable avid virus-host interactions during HIV-1 infection

**DOI:** 10.1038/s41467-022-33662-6

**Published:** 2022-10-06

**Authors:** Guochao Wei, Naseer Iqbal, Valentine V. Courouble, Ashwanth C. Francis, Parmit K. Singh, Arpa Hudait, Arun S. Annamalai, Stephanie Bester, Szu-Wei Huang, Nikoloz Shkriabai, Lorenzo Briganti, Reed Haney, Vineet N. KewalRamani, Gregory A. Voth, Alan N. Engelman, Gregory B. Melikyan, Patrick R. Griffin, Francisco Asturias, Mamuka Kvaratskhelia

**Affiliations:** 1 Division of Infectious Diseases, Anschutz Medical Campus, University of Colorado School of Medicine, Aurora, CO 80045 USA; 2 Department of Biochemistry & Molecular Genetics, Anschutz Medical Campus, University of Colorado School of Medicine, Aurora, CO 80045 USA; 3 Department of Molecular Medicine, The Scripps Research Institute, Jupiter, FL 33458 USA; 4 Institute of Molecular Biophysics, Department of Biological Sciences, Florida State University, Tallahassee, FL 32306 USA; 5 Department of Pediatrics, Emory University, Atlanta, GA 30322 USA; 6 Department of Cancer Immunology & Virology, Dana-Farber Cancer Institute, Boston, MA 02215 USA; 7 Department of Medicine, Harvard Medical School, Boston, MA 02115 USA; 8 Department of Chemistry, Chicago Center for Theoretical Chemistry, Institute for Biophysical Dynamics, and James Franck Institute, The University of Chicago, Chicago, IL 60637 USA; 9 Center for Cancer Research, National Cancer Institute, Frederick, MD 21702 USA

**Keywords:** Virus-host interactions, Cryoelectron microscopy, Retrovirus, HIV infections

## Abstract

Cellular proteins CPSF6, NUP153 and SEC24C play crucial roles in HIV-1 infection. While weak interactions of short phenylalanine-glycine (FG) containing peptides with isolated capsid hexamers have been characterized, how these cellular factors functionally engage with biologically relevant mature HIV-1 capsid lattices is unknown. Here we show that prion-like low complexity regions (LCRs) enable avid CPSF6, NUP153 and SEC24C binding to capsid lattices. Structural studies revealed that multivalent CPSF6 assembly is mediated by LCR-LCR interactions, which are templated by binding of CPSF6 FG peptides to a subset of hydrophobic capsid pockets positioned along adjoining hexamers. In infected cells, avid CPSF6 LCR-mediated binding to HIV-1 cores is essential for functional virus-host interactions. The investigational drug lenacapavir accesses unoccupied hydrophobic pockets in the complex to potently impair HIV-1 inside the nucleus without displacing the tightly bound cellular cofactor from virus cores. These results establish previously undescribed mechanisms of virus-host interactions and antiviral action.

## Abstract (web summary)

Host proteins CPSF6, NUP153, and SEC24C are vital for HIV-1 infection. They bind to the viral capsid protein and contribute to shuttling of virions through the cytoplasm (SEC24C), import into the nucleus (NUP153 and CPSF6) and subsequent trafficking to preferred integration sites (CPSF6). Here, Wei et al. combine structural, biochemical and virological assays to emphasize the importance of prion-like low complexity domains surrounding short phenylalanine-glycine regions in binding and increasing the avidity when interacting with viral capsid.

## Introduction

The cellular proteins cleavage and polyadenylation specificity factor 6 (CPSF6), nucleoporin NUP153 and SEC24C contribute to early steps of HIV-1 replication. These proteins engage with the outer shell of the HIV-1 core, which is composed of the capsid protein (CA) arranged into large hexameric lattices as well as into 12 pentamers needed to form the closed conical structure. The HIV-1 core interior contains the viral RNA genome and key viral enzymes (reverse transcriptase and integrase) that catalyze conversion of the single-stranded viral RNA into the double-stranded DNA copy and its subsequent integration into host cell chromatin. SEC24C stabilizes HIV-1 cores and co-traffics with them across the cytoplasm^1^. NUP153 and CPSF6 mediate nuclear import of viral cores^2,3^. Furthermore, CPSF6 navigates HIV-1 inside the nucleus to nuclear speckles (NSs) and thereby promotes integration in speckle-associated domains (SPADs) and gene-rich regions^4,5^.

Prior X-ray structures have revealed that short phenylalanine-glycine (FG)-containing peptides of CPSF6, NUP153 and SEC24C bind to CA hexamers in the hydrophobic cavity created by two adjoining CA subunits^1,6^. However, the interactions of FG peptides from CPSF6 and NUP153 exhibited very low binding affinity to an isolated CA hexamer, which may not be sufficient for functional virus-host interactions. The structural and mechanistic bases for interactions of CPSF6, NUP153 and SEC24C with biologically relevant mature CA lattices are unknown.

HIV-1 CA and, in particular, the hydrophobic cavity that binds the FG peptides, is an important therapeutic target. The long-acting, ultra-potent CA inhibitor lenacapavir (LEN), which represents a profoundly novel antiviral approach and is currently in Phase III clinical trials, selectively binds to this hydrophobic pocket^7,8^. Therefore, achieving mechanistic and structural understanding of how the FG-containing cofactors engage with biologically relevant CA lattices will help us to better define these critical virus-host interactions as therapeutic targets as well as elucidate the mode of action of LEN.

Here, we have discovered that, unlike the respective FG peptides, CPSF6, NUP153 and SEC24C avidly bind to hexameric CA lattices. These interactions are mediated by prion-like low complexity regions (LCRs) that encompass the previously described FG peptides within each of these proteins. Our cryo-EM studies coupled with hydrogen-deuterium exchange and mass spectrometry (HDX-MS) experiments reveal that LCR-LCR interactions enable polyvalent assembly of CPSF6 onto curved hexameric CA lattices. In turn, CPSF6-CPSF6 interactions are templated by binding of the embedded FG peptides to a subset of cognate hydrophobic CA pockets positioned along adjoining hexamers. Experiments conducted in infected cells have validated the structural findings by demonstrating an essential role of the CPSF6 LCR for functional virus-host interactions. Moreover, our characterization of CPSF6 interactions with HIV-1 CA provides unexpected insight into the exceptionally potent antiviral activity of LEN. The inhibitor can engage unoccupied hydrophobic pockets in curved hexameric CA lattices even after polyvalent assembly of CPSF6, and potently impair HIV-1 inside the nucleus without displacing tightly bound cellular cofactors from virus cores.

## Results

### Prion-like LCRs flanking the FG peptides enable avid binding of CPSF6, NUP153, and SEC24C to HIV-1 cores

To date, only the low affinity binding of 15-mer anchoring FG peptides from CPSF6 and NUP153 to crosslinked CA hexamers have been reported^6^. Here, surface plasmon resonance (SPR) based-experiments demonstrated that 15-mer FG peptides CPSF6_313-327_ and NUP153_1409-1423_ bound to CA hexamers with *K*_*d*_ values of ~71 and 131 µM, respectively (Supplementary Table 1), which is consistent with the previous results^6^. The 15-mer SEC24C_228-242_ FG peptide, which had not been examined previously, exhibited an even lower binding affinity (*K*_*d*_ of ~1070 µM) to crosslinked CA hexamers (Supplementary Table 1).

**Fig. 1:**
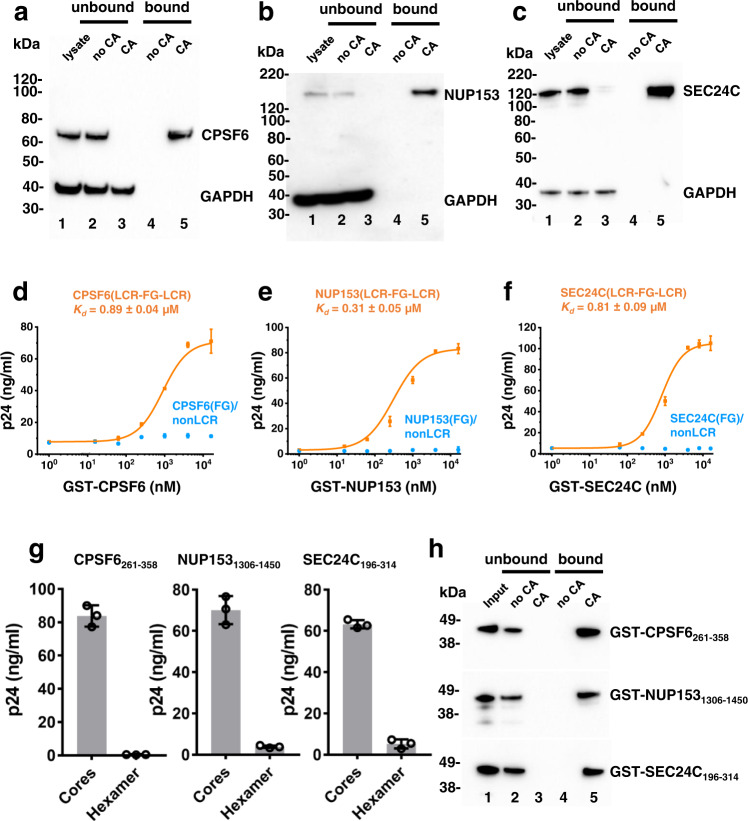
Biochemical characterization of CPSF6, NUP153 and SEC24C interactions with HIV-1 cores and CA tubes. Representative immunoblots showing CA nanotube mediated co-pelleting of endogenous CPSF6 (**a**), NUP153 (**b**) and SEC24C (**c**) from MT4 cell lysates. The proteins of interest were visualized by antibodies ab175237(Abcam) against CPSF6, NB100-93329 (Novus) against NUP153, ab122633 (Abcam) against SEC24C. Lane 1: cell lysate. Lane 2: supernatant or unbound fraction after pelleting in the absence of CA tubes. Lane 3: supernatant or unbound fraction after co-pelleting with pre-formed CA tubes. Lane 4: pelleted or bound fraction in the absence of CA tubes. Lane 5: pelleted or bound fraction in the presence of CA tubes. The experiments were repeated 3 times independently with similar results. Quantitation of GST-mediated affinity pull-down of native HIV-1 cores bound to indicated concentrations of GST-CPSF6_261-358_(LCR-FG-LCR) vs GST-CPSF6(FG)/nonLCR (**d**), GST-NUP153_1306-1450_(LCR-FG-LCR) vs GST-NUP153(FG)/nonLCR (**e**); and GST-SEC24C_196-314_(LCR-FG-LCR) vs GST-SEC24C(FG)/nonLCR (**f**). The results were analyzed by Origin 2019 (v.9.6) software to determine binding *K*_*d*_ values. Each data point represents mean values + /− SD from three independent experiments. Source data are provided as a Source Data file. **g** Quantitation of GST-mediated affinity pull-down of isolated native HIV-1 cores vs crosslinked CA hexamers with 2 μM GST-CPSF6_261-358_(LCR-FG-LCR) (left), GST-NUP153_1306-1450_(LCR-FG-LCR) (middle) and GST-SEC24C_196-314_(LCR-FG-LCR) (right). Mean values + /− SD from three independent experiments are shown. Source data are provided as a Source Data file. **h** Representative immunoblots of three independent experiments showing co-pelleting of GST-CPSF6_261-358_(LCR-FG-LCR) (top), GST-NUP153_1306-1450_(LCR-FG-LCR) (middle) and GST-SEC24C_196-314_(LCR-FG-LCR) (bottom) with pre-formed CA nanotubes. The experiments were repeated 3 times independently with similar results.

Could such low affinity binding be sufficient for functional virus-host interactions or do other regions in CPSF6, NUP153 and SEC24C enable avid binding of these proteins to HIV-1 cores? We first addressed this question by examining the binding efficiency of endogenous, full-length CPSF6, NUP153 and SEC24C to pre-formed WT CA nanotubes, which closely mimic curved hexameric lattices in native HIV-1 cores^9,10^. All three cellular proteins, which were present at very low (nM) concentrations in lysates of MT4 cells, nearly completely co-pelleted with WT CA nanotubes, which was indicative of high affinity binding (Fig. 1a–c). As a control, we monitored GAPDH, which, although readily detected in cell lysates and unbound fractions, was absent from CA co-pelleted fractions. When asking what additional regions beyond the FG peptide enabled the avid binding of these proteins to CA, we noticed that the 15-mer FG peptides in CPSF6, NUP153 and SEC24C are embedded in substantially larger prion-like LCRs (Supplementary Fig. 1). The LCRs are distinct, disordered, compositionally biased regions commonly found in prion-like proteins and RNA binding proteins^11,12^. The prion-like LCRs show a strong bias for uncharged amino acids and can self-assemble when provided a relevant template, which can involve self-templating as in the case of prions, or templating on another macromolecular surface^11,13,14^. Indeed, LCRs in NUPs, including NUP153, are known to contribute to template mediated self-assembly of NUPs into larger structures, such as hydrogels^13,15^. Furthermore, LCRs in RNA binding proteins mediate liquid-phase transitions that drive ribonucleoprotein granule assembly^16^. However, it is not known if and how LCRs from CPSF6, NUP153 and SEC24C affect interactions of these cellular proteins with HIV-1 cores.

Accordingly, we next examined binding affinities of larger CPSF6, NUP153 and SEC24C fragments, in which the FG peptides were embedded within their respective LCRs, to native cores isolated from HIV-1 virions. For this, we prepared purified recombinant GST-CPSF6_261-358_(LCR-FG-LCR), GST-NUP153_1306-1450_(LCR-FG-LCR) and GST-SEC24C_196-314_(LCR-FG-LCR) (Supplementary Fig. 2a, c). Strikingly, the larger fragments from all three proteins bound to HIV-1 cores with sub-µM *K*_*d*_ values (Fig. 1d–f). We also examined interactions of isolated HIV-1 cores with control proteins GST-CPSF6(FG)/nonLCR, GST-NUP153(FG)/nonLCR and GST-SEC24C(FG)/nonLCR, where the 15-mer FG peptides were fully preserved but flanking LCR sequences were replaced by non-specific flexible sequences of typical amino acid composition (Supplementary Fig. 2b, d). Consistent with low affinity binding of the 15-mer peptides to cross-linked hexamers (Supplementary Table 1)^6^, GST-CPSF6(FG)/nonLCR, GST-NUP153(FG)/nonLCR and GST-SEC24C(FG)/nonLCR failed to effectively bind to HIV-1 cores (Fig. 1d–f). These results demonstrate the importance of LCRs for high affinity binding of CPSF6, NUP153 and SEC24C to HIV-1 cores.

We then compared binding to native cores vs isolated crosslinked CA hexamers. The results in Fig. 1g show that GST-CPSF6(LCR-FG-LCR), GST-NUP153(LCR-FG-LCR) and GST-SEC24C(LCR-FG-LCR) effectively bound to native cores but not to isolated crosslinked hexamers. Since native cores contain hexameric CA lattices and CA pentamers, we also examined binding of GST-CPSF6(LCR-FG-LCR), GST-NUP153(LCR-FG-LCR) and GST-SEC24C(LCR-FG-LCR) to tubular CA assemblies which contain only hexameric CA lattices. The results in Fig. 1h show that like the full-length endogenous proteins (Fig. 1a–c), the LCR-FG-LCR constructs from CPSF6, NUP153 and SEC24C effectively bound to pre-formed hexameric CA lattices. Collectively, our biochemical results indicate that avid binding of CPSF6, NUP153 and SEC24C to HIV-1 cores requires interactions of their LCR-FG-LCR segments with hexameric CA lattices.

### The LCR is essential for CPSF6 interaction with HIV-1 cores in infected cells

Over a decade of research into the roles of CPSF6 in HIV-1 infection has established a battery of powerful virology assays. We exploited these methodologies to examine contributions of the CPSF6 LCR to HIV-1 ingress. To specifically interrogate possible roles of the LCR, we designed chimeric constructs in the context of full-length CPSF6, where native LCRs, which flank the FG peptide (CPSF6_313-327_), were replaced with either nonLCR or alternative prion-like LCRs from other proteins that do not interact with HIV-1 CA^1^ (Supplementary Fig. 3). The two examples of nonLCR regions were chosen from Beta-adducin (ADD2) and Neuromodulin (NEURM)^17,18^, as these protein segments are known to be highly flexible like the CPSF6 LCR. However, unlike CPSF6 LCR, the selected nonLCRs contain typical levels of charged residues^17,18^ (Supplementary Fig. 4a). The two examples of alternative, prion-like LCRs were chosen from RNA-binding protein FUS (FUS) and Cyclin-dependent kinase 19 (CDK19)^19–21^, which in common with the CPSF6 LCR, are both flexible and exhibit a strong bias for uncharged amino acids (Supplementary Fig. 4a)^22^. AlphaFold structural predictions for WT CPSF6_261-358_ and corresponding chimeric constructs revealed that in each case the CA binding CPSF6 FG peptide adopted a “U” shaped conformation, whereas flanking LCR and nonLCR sequences were, as designed, disordered (Supplementary Fig. 4b). In the experimental setting with the full-length chimeric proteins, all other CPSF6 sequences were preserved (Supplementary Fig. 3a). These constructs were expressed in CPSF6 KO HEK293T (CKO) cells by gammaretroviral transduction (Supplementary Fig. 3b, c), and various roles of CPSF6 in HIV-1 infection were monitored in the resulting cell lines.

**Fig. 2:**
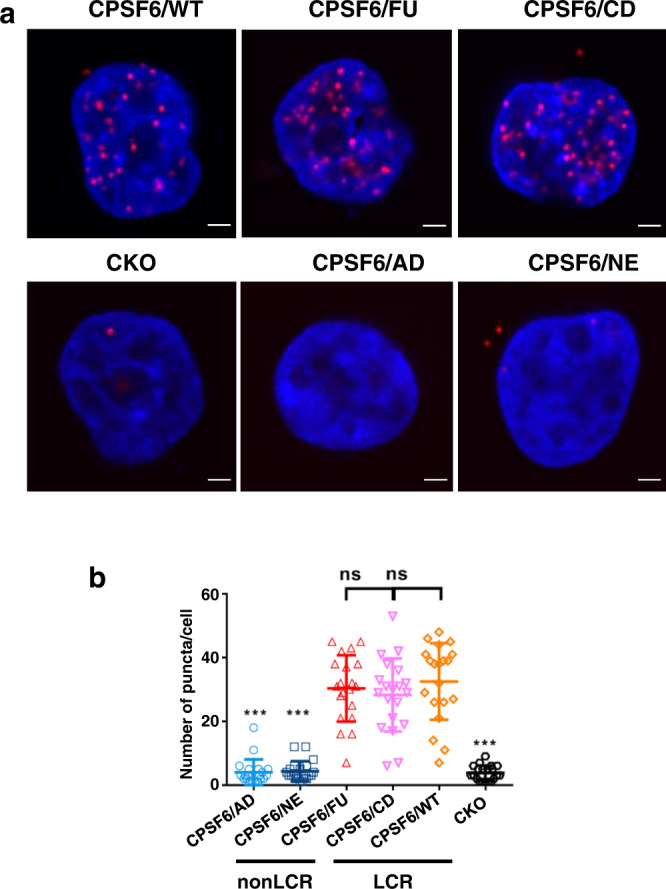
LCR sequences are critical for CPSF6 interaction with HIV-1 cores in infected cells. **a** PLAs showing association of HA-tagged ectopically expressed WT and chimeric CPSF6 proteins with incoming HIV-1 particles in HEK293T CKO cells. The cells were fixed at 6 hpi and PLAs were performed using anti-HA (ab236632, Abcam) and anti-HIV-1 CA specific antibodies (ARP-4121, NIH AIDS Reagent Program). The representative images show PLA puncta (red) and nuclei stained with DAPI (blue). Scale bar is 2 μm. **b** Quantitative results showing numbers of PLA puncta per cell by analyzing twenty-five cells for each sample. The averaged data (+/− SD) from three independent experiments are shown. Statistical significance of comparison of WT versus the chimeric CPSF6 proteins was determined by Student’s two-sample, two-tailed *t*-test. CPSF6/AD: *p* = 3.1E-12; CPSF6/NE: *p* = 2.4e-12; CPSF6/FU: *p* = 0.54; CPSF6/CD: *p* = 0.26; CKO: *p* = 8.8E-13. *P* > 0.05 was considered not significant (ns) and *p* < 0.0001 was considered highly significant (***). Source data are provided as a Source Data file. AD ADD2, NE NEURM, FU FUS, CD CDK19, WT wild-type CPSF6, CKO CPSF6 knock-out, LCR low complexity region.

We tested the association of chimeric proteins with HIV-1 cores in infected cells with proximity ligation assays (PLAs). The results in Fig. 2 show that, unlike CPSF6/WT that efficiently interacts with HIV-1 cores, the chimeric proteins CPSF6/AD and CPSF6/NE containing non-LCRs fail to associate with cores in infected cells. Strikingly, substitution of native LCRs with alternative, prion-like LCRs in CPSF6/FU and CPSF6/CD provided effective gain-of-binding (Fig. 2). Control immuno-staining experiments revealed that all chimeric proteins, including nonLCR and LCR constructs, exhibited nuclear distribution that closely mimicked WT CPSF6 (Supplementary Fig. 3c). The unaltered nuclear localization is likely due to preservation of the C-terminal RS domain, which is important for nuclear import of CPSF6^23^, in the chimeric proteins (Supplementary Fig. 3a).

Complementary biochemical assays tested binding of the purified recombinant GST-CPSF6_261-358_ and corresponding chimeric proteins to isolated HIV-1 cores (Supplementary Fig. 5). Results in Supplementary Fig. 5c show that chimeric GST-CPSF6(FG)/AD and GST-CPSF6(FG)/NE proteins, which contained the 15-mer FG peptide but lacked the flanking LCR content, failed to bind HIV-1 cores. In contrast, the chimeric proteins GST-CPSF6(FG)/FU and GST-CPSF6(FG)/CD, where the 15-mer peptide was flanked by alternative, prion-like LCRs, avidly bound to HIV-1 cores with *K*_*d*_ values comparable to GST-CPSF6_261-358_/WT. Collectively, our PLA and biochemical assays indicate that the LCRs flanking the FG peptide enable avid CPSF6 interaction with HIV-1 cores in infected cells and in vitro.

### The LCR is required for CPSF6’s function in HIV-1 infection

CPSF6 mediates HIV-1 nuclear import and the subsequent transport of viral replication complexes (VRC) to NSs, which in turn results in highly selective HIV-1 integration into SPADs and gene rich regions^4,5,24^. While CPSF6 depletion does not detectably influence HIV-1 infection in HEK293T cells, in the absence of CPSF6 VRCs are uncharacteristically localized at the nuclear periphery resulting in integration within adjacent lamina-associated domains (LADs)^4,5,25^.

**Fig. 3:**
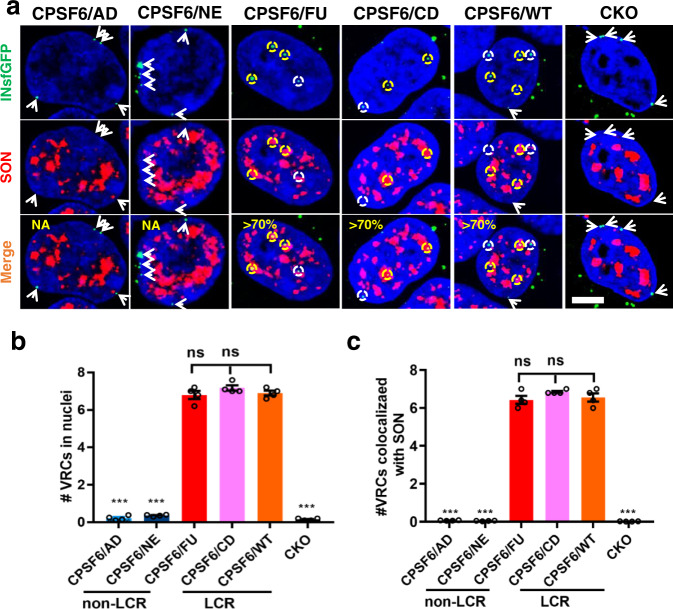
CPSF6 LCR effects on HIV-1 nuclear import and targeting to NS. **a**–**c** HEK293T CKO cells were back-complemented with indicated HA tagged CPSF6 (CPSF6) proteins and infected with VSV-G pseudotyped HIV-1 viruses (MOI 1) labeled with INsfGFP (green) for 4 h. Cells were fixed and immunostained for SON nuclear speckle (NS) marker (red) and nuclei with SiR-Hoechst (blue). **a** Single Z-stack images. Scale bar is 5 µm. **b** Quantitation of the number of IN-labeled viral replication complexes (VRCs) >0.5 µm of the nuclear periphery. **c** Quantification of IN-VRCs colocalized with SON-NSs. Mean values obtained from each experiment and SEM from a total of 4 independent experiments are shown in **b**, **c**. Each independent experiment analyzed >30 nuclei for each construct. Statistical significances of comparison of CPSF6/WT versus CPSF6/AD, CPSF6/NE, CPSF6/FU, CPSF6/CD and CKO (in **b**, **c**) were determined by Student’s two-sample, two-tailed *t*-test. *P*-values of CPSF6/AD, CPSF6/NE, CPSF6/FU, CPSF6/CD and CKO are 1.1E-05, 1.7E-05, 0.6, 0.09, 1.7E-05 (in **b**) and 8.8E-05, 9.2E-05, 0.69, 0.22, 8.5E-05 (in **c**), respectively. *P* > 0.05 was considered not significant (ns) and *p* < 0.001 was considered highly significant (***). Source data are provided as a Source Data file. CPSF6/AD CPSF6/ADD2, CPSF6/NE CPSF6/NEURM, CPSF6/FU CPSF6/FUS, CPSF6/CD CPSF6/CDK19, CPSF6/WT CPSF6/wild-type CPSF6, CKO CPSF6 knock-out, LCR low complexity region.

Here, we examined how chimeric proteins affected HIV-1 infectivity, nuclear import, colocalization with NSs, and SPAD-proximal integration targeting. Akin to WT CPSF6, overexpression of nonLCR or LCR containing chimeric proteins did not detectably alter HIV-1 infectivity in CKO cells (Supplementary Fig. 6a). In CKO cells expressing the chimeric nonLCR containing CPSF6/AD and CPSF6/NE proteins VRCs failed to effectively penetrate deeper inside the nucleus and instead, they accumulated at the nuclear periphery over the course of 8 h (Fig. 3 and Supplementary Fig. 6b–d). In contrast, cells expressing CPSF6/FU and CPSF6/CD, which contained alternative, prion-like LCRs flanking the FG peptide, supported HIV-1 transport inside the nucleus to the sites of NSs with similar efficiency and kinetics as WT CPSF6-expressing cells (Fig. 3 and Supplementary Fig. 6b–d). These results indicate that nonLCR containing chimeric CPSF6/AD and CPSF6/NE proteins fail to engage with HIV-1 during infection, whereas LCR containing CPSF6/FU and CPSF6/CD can effectively substitute for WT CPSF6 during HIV-1 nuclear import and transport to NSs.

HIV-1 trafficking to NS is accompanied by marked accumulation of CPSF6 around HIV-1 cores (Supplementary Fig. 7a)^4,26,27^. Unlike CPSF6/WT, the nonLCR based chimeric CPSF6/AD and CPSF6/NE proteins lacked this property (Supplementary Fig. 7b, c). By contrast, LCR containing chimeric CPSF6/FU and CPSF6/CD proteins strongly accumulated around HIV-1 cores in the nucleus of infected cells (Supplementary Fig. 7b, c).

**Fig. 4:**
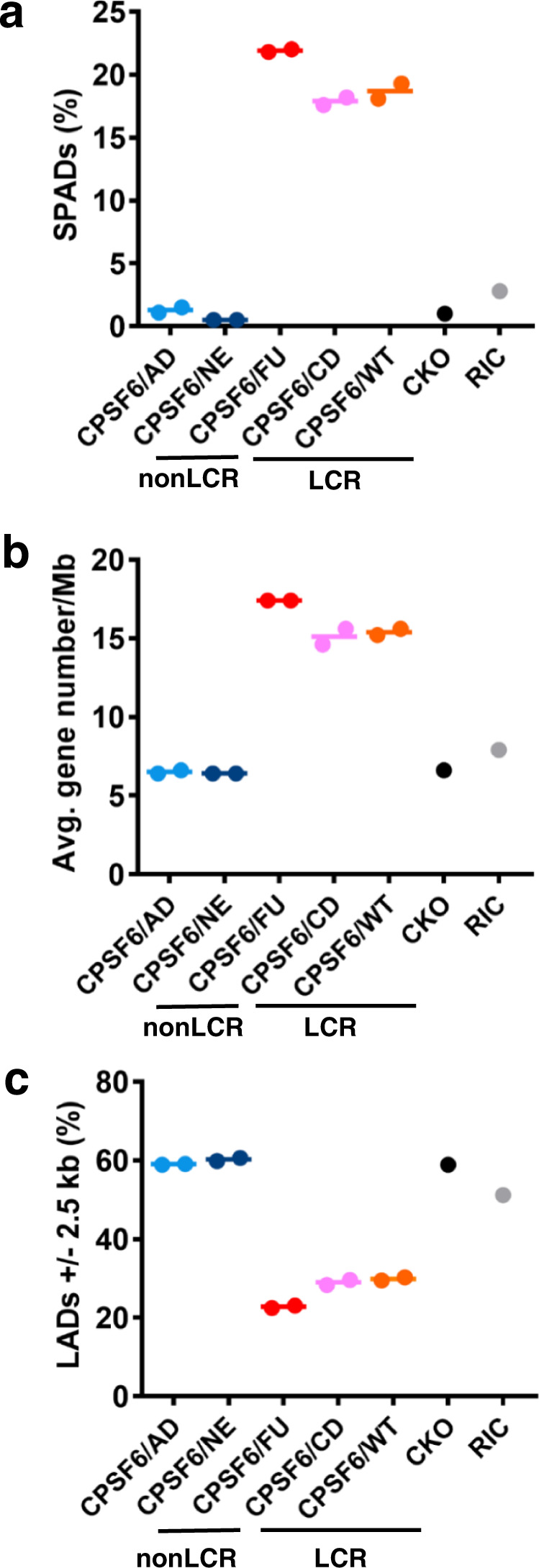
The CPSF6 LCR effects on integration site selection. Distribution of HIV-1 integration sites in SPADs (**a**), gene dense regions (**b**), and LADs (**c**). CPSF6/AD, CPSF6/NE, CPSF6/FU, and CPSF6/CD constructs were expressed alongside WT CPSF6 (CPSF6/WT) in CKO cells. The random integration control (RIC) was previously described^25^. With the exception of CKO cells, all experiments were done in duplicate. Data points from two independent experiment are shown and corresponding mean values are indicated by lines (also see Supplementary Table 2). Source data are provided as a Source Data file. CPSF6/AD CPSF6/ADD2, CPSF6/NE CPSF6/NEURM, CPSF6/FU CPSF6/FUS, CPSF6/CD CPSF6/CDK19, CPSF6/WT CPSF6/wild-type CPSF6, CKO CPSF6 knock-out, LCR low complexity region.

In complementary assays, we determined sites of HIV-1 integration selectivity (Fig. 4 and Supplementary Table 2). Expression of the chimeric proteins CPSF6/AD and CPSF6/NE, which lacked the LCR context for the FG peptide, exhibited HIV-1 integration patterns similar to those observed in CKO cells (Fig. 4 and Supplementary Table 2). In contrast, chimeric proteins CPSF6/FU and CPSF6/CD containing alternative LCRs directed HIV-1 integration into SPADs (Fig. 4a and Supplementary Table 2) and gene-dense regions (Fig. 4b and Supplementary Table 2) similarly to WT CPSF6. Conversely, the CPSF6/FU and CPSF6/CD chimeric proteins redirected HIV-1 integration away from LADs (Fig. 4c and Supplementary Table 2). Collectively, these loss- and gain-of-function assays indicate an essential role of the LCR for functional CPSF6 interactions in HIV-1-infected cells.

### Cryo-EM analysis of GST-CPSF6_261-358_(LCR-FG-LCR) bound to IP6-stabilized CA nanotubes

**Fig. 5:**
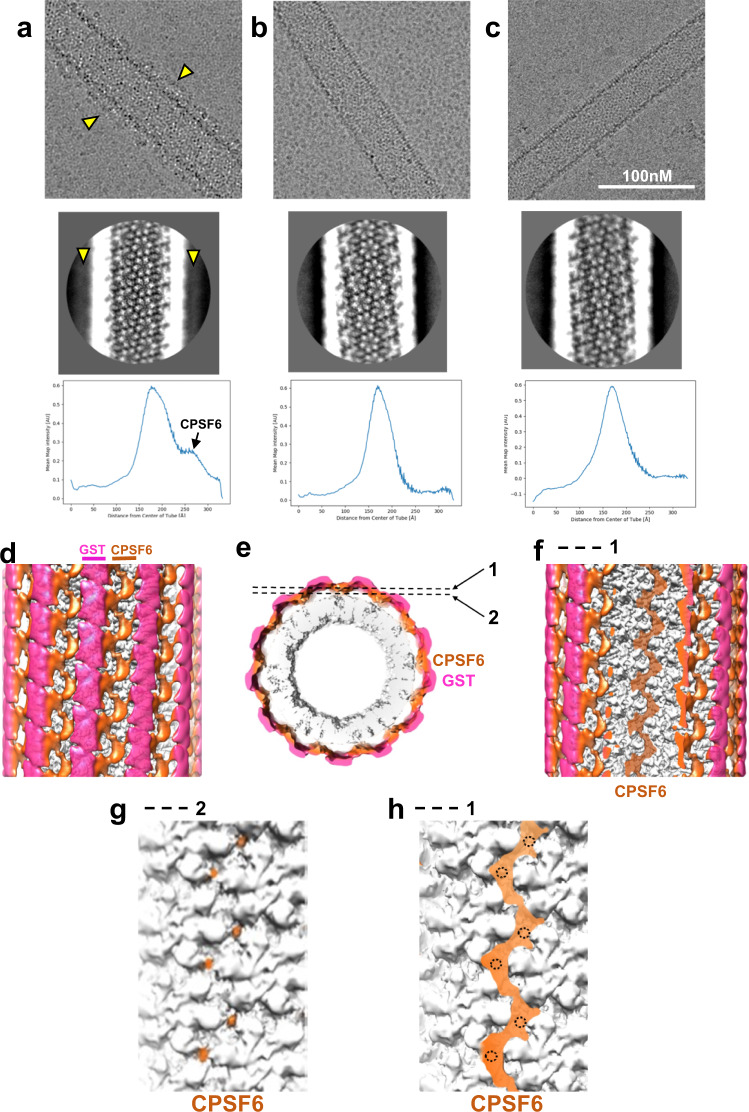
Cryo-EM analysis of CPSF6(LCR-FG-LCR) binding to IP6-stabilized CA tubes. Images of CA(A92E) tubes in the presence of WT GST-CPSF6_261-358_ (**a**); GST-CPSF6_261-358_(ΔFG), which contains LCR sequences but lacks the CA binding FG residues (**b**); and GST-CPSF6/AD(FG-nonLCR), which contains the CA binding 15-mer FG peptide but where flanking LCRs are replaced with nonLCR sequences from ADD2 (**c**). Partially ordered additional density (indicated by yellow arrow heads) along the surface of CA nanotubes was consistently observed in CA + WT GST-CPSF6_261-358_(LCR-FG-LCR) samples (**a**, top). This additional density was also evident in 2D class averages (**a**, middle, yellow arrow heads) and in 1D density profiles (**a**, bottom). In contrast, no additional density was apparent after addition of GST-CPSF6_261-358_(ΔFG) (**b**) or GST-CPSF6/AD(FG-nonLCR) (**c**) to CA nanotubes. The experiments were repeated 3 times independently with similar results. Front (**d**) and end (**e**) views of a cryo-EM map (~7.9 Å overall resolution) of a CA(A92E) nanotube in the presence of GST-CPSF6_261-358_(LCR-FG-LCR), filtered by local resolution and colored by radial distance from the center of the nanotube (CA in gray, CPSF6 in orange, GST dimers in semi-transparent magenta to help visualize CA and CPSF6 density). Front views of the same cryo-EM map, sliced at the two planes indicated in **e**, show continuous CPSF6 density arising from LCR interactions (**f**), as well as CPSF6 discrete contact points with the CA hexamer lattice (**g**) and their correspondence to continuous LCR density (**h**) (the doted circles mark the positions of CPSF6-CA contact points shown in **g**).

To obtain structural insight into how CPSF6 LCRs contribute to binding avidity we used cryo-EM to image GST-CPSF6_261-358_(LCR-FG-LCR) bound to IP6-stabilized CA nanotubes (Fig. 5 and Supplementary Figs 9–11, Supplementary Table 3**)**. Images of nanotubes incubated with GST-CPSF6(LCR-FG-LCR), and corresponding class averages, consistently showed lines of non-CA density extending alongside the nanotubes (Fig. 5a). In contrast, no additional density was observed when pre-formed, IP6-stabilized CA nanotubes were incubated with GST-CPSF6(ΔFG) (Fig. 5b) or with GST-CPSF6(FG)/nonLCR in which the 15-mer FG peptide was preserved but flanking LCRs were replaced by nonLCR sequences (Fig. 5c). Biochemical results in Supplementary Fig. 8 explain why GST-CPSF6 density was not observed in cryo-EM class averages upon addition of GST-CPSF6(ΔFG) or GST-CPSF6(FG)/non-LCR construct to CA nanotubes.

In agreement with what was observed in class averages, a cryo-EM map of GST-CPSF6(LCR-FG-LCR) bound to IP6-stabilized CA nanotubes (overall resolution 7.9 Å) (Fig. 5 and Supplementary Table 3), filtered according to local resolution, showed a well-ordered CA nanotube with more flexible CPSF6 density (Supplementary Fig. 9). The CPSF6 density is connected to the CA hexamers at discrete positions (Fig. 5d–h and Supplementary Fig. 10), while GST tag density is distal from the helical nanotube. CPSF6 contacts to adjoining CA hexamers most likely correspond to FG peptide binding to the cognate CA hydrophobic pockets seen in published^6,28^ and our crystal structures of the CA hexamer + IP6 + CPSF6_313–327_ peptide complex (Supplementary Fig. 12 and Supplementary Table 4). Importantly, we note that short FG peptide binding to isolated CA hexamers seen in our X-ray structure (Supplementary Fig. 12), and CPSF6(LCR-FG-LCR) binding to the mature CA lattice in our cryo-EM map (Fig. 5g) exhibit different stoichiometries. This is discussed below in the context of LEN’s mode of action.

CPSF6 contact points on CA were connected by continuous CPSF6 density running along the flattest helical direction of the CA nanotube between adjoining rows of CA hexamers (Fig. 5f, h). Both GST and CPSF6 densities were absent from a cryo-EM map when GST-CPSF6(ΔFG) was added to CA nanotubes (Supplementary Fig. 10b, c). The pattern of CPSF6 density observed in cryo-EM maps of GST-CPSF6(LCR-FG-LCR) bound to IP6-stabilized CA tubes calculated from images of tubes with different helical symmetries is conserved, with the only significant difference being a change in the orientation of connected CPSF6 density corresponding to the change in the orientation of rows of CA hexamers in the helical tubes (Supplementary Fig. 11a).

Overall, the cryo-EM map of GST-CPSF6(LCR-FG-LCR) bound to IP6-stabilized CA tubes defined the distribution of CPSF6 density including its contact points to CA hexamers as well as the position of the N-terminal GST tag. Based on these constraints, we were able to derive an initial model for interaction of CPSF6 with the CA hexamer lattice (Supplementary Fig. 11b), which suggests that the LCR sequence N-terminal of the FG peptide primarily accounts for the bulk of CPSF6 density apparent in the cryo-EM map. This interpretation is supported by additional independent results (see the next section). While the limited resolution of the cryo-EM map did not allow us to delineate different LCR conformations or specific details of LCR-LCR interactions, our principal cryo-EM findings (Fig. 5g, h) strongly point to a bimodal assembly of CPSF6 onto CA nanotubes: i) anchoring CPSF6 FG peptides (Supplementary Fig. 11b, green ellipses) bind to adjoining CA hexamers by directly engaging a cognate hydrophobic pocket from each hexamer; ii) LCR-LCR interactions (LCRs from adjacent CPSF6 molecules are represented by yellow and red lines in Supplementary Fig. 11b) enable polyvalent CPSF6 assembly on the CA hexamer lattice and drive enhanced CPSF6 binding affinity.

In the cryo-EM map of GST-CPSF6(LCR-FG-LCR) bound to IP6-stabilized CA nanotubes, density corresponding to GST tags (necessary for preparation of sufficient quantities of recombinant CPSF6 proteins for structural studies) extends outwards and away from the CA tubes + CPSF6(LCR-FG-LCR) complex (Fig. 5e and Supplementary Fig. 10c). These GST-tags do not contribute to either CPSF6-CA or CPSF6-CPSF6 interactions. This is consistent with biochemical data comparing GST-CPSF6(LCR-FG-LCR) vs GST-CPSF6(FG)/nonLCR constructs (Supplementary Fig. 8), which clearly pointed to a critical role of the LCRs for high affinity binding and argue against GST contributions to these interactions. Furthermore, our extensive virology experiments (Figs. 2–4), which did not employ GST-tag constructs, also highlighted the importance of the LCRs for functional virus-host interactions. It is worth noting that GST positioning in the cryo-EM map suggests that the similarly-sized CPSF6_1-261_ fragment, which is absent in our construct due to solubility issues, could similarly extend outwards from CA capsids in the context of full length CPSF6, allowing polyvalent LCR-FG-LCR interactions like the ones observed with the CPSF6 construct used in our cryo-EM studies.

### Conformational analysis of GST-CPSF6_261-358_(LCR-FG-LCR) bound to CA-hexamer lattice from all-atom (AA) molecular dynamics (MD) simulations

In cryo-EM maps, CPSF6 density was observed along the interface formed by rows of adjoining CA hexamers, illustrating polyvalent assembly of CPSF6 templated by the CA lattice. To characterize the CPSF6 conformations and the LCR-LCR interactions driving the polyvalent assembly of CPSF6 we performed AA MD of GST-CPSF6_261-358_ bound to the CA hexamer lattice. Because performing all-atom simulations of CPSF6 assembly templated by a CA nanotube for a large array of CA hexamers would be prohibitively expensive, we simulated a minimal system of three GST-CPSF6_261-358_ copies bound to a CA lattice consisting of 4 hexamers. The following integrative modeling approach was employed. First, we positioned the GST dimers based on the location of the respective cryo-EM density. FG peptides bound to CA hexamer complexes were generated based on the X-ray crystal structure of the CA hexamer+ IP6 + CPSF6_313–327_ complex (Supplementary Fig. 12) and placed onto adjoining CA hexamers in accordance with the cryo-EM maps (Fig. 5). Finally, the N-terminal LCR for each CPSF6 chain was modeled based on the density observed in the cryo-EM maps. The C-terminal LCR segment for each CPSF6 chain was appended to the FG peptide to avoid overlapping contacts with N-terminal LCR segments of the adjoining CPSF6 chains and the CA hexamers. This initial arrangement of three GST-CPSF6_261-358_ chains bound to the 4 CA hexamer lattice fragment was used as the starting point for performing three independent 1 µs-long simulations (Supplementary Fig. 13).

To characterize the conformational ensembles of the tri-CPSF6 complex we clustered the conformations sampled in the AA MD trajectories based on a root-mean-square deviation-based criteria (see details in Materials and Methods). In all major clusters (Supplementary Fig. 13d), the CPSF6 chains remained inter-associated and formed a highly interactive network spanning multiple CA hexamers. In the mutually associated state, the tri-CPSF6 complex did not adopt a specific dominant conformation, but dynamically sampled multiple conformations that cumulatively contribute to the overall spatial density of CPSF6. To facilitate direct comparison with the cryo-EM maps we calculated the time-averaged 3D density of C_a_ atoms in CPSF6_261-358_ (Supplementary Fig. 13d). The resulting density map of the tri-CPSF6 complex revealed its spatial distribution relative to the CA hexamers. During the simulations, CPSF6 FG peptides remain localized at the initially modeled hydrophobic CA pockets. In contrast, the density of the N-terminal and C-terminal LCR regions was distributed along the interface formed by the CA hexamer rows and extended outwards from the CA lattice surface.

Taken together, the results from our AA MD simulations demonstrate the following. The 15-mer FG peptide directly binds in the hydrophobic CA pocket in agreement with prior and current biochemical and X-ray crystallography data. The flanking N-terminal and C-terminal LCRs populate the region between the rows of CA hexamers above the CA lattice. The N-terminal LCRs primarily contribute to the CPSF6-CPSF6 assembly forming a network of interacting CPSF6 chains templated by the CA lattice. Finally, unlike what one would expect for interactions in an array of proteins with well-defined secondary structures, an ensemble of N-terminal LCR conformations mediate the formation of a highly interactive network of primarily unstructured CPSF6 chains. The conformational ensembles of the tri-CPSF6 complex observed in our AA MD simulations can be interpreted as the minimal LCR-mediated motifs for large-scale assembly of CPSF6 templated by a mature CA lattice (Fig. 5h).

### HDX-MS analysis of GST-CPSF6(LCR-FG-LCR) interactions with IP6-stabilized CA tubes

**Fig. 6:**
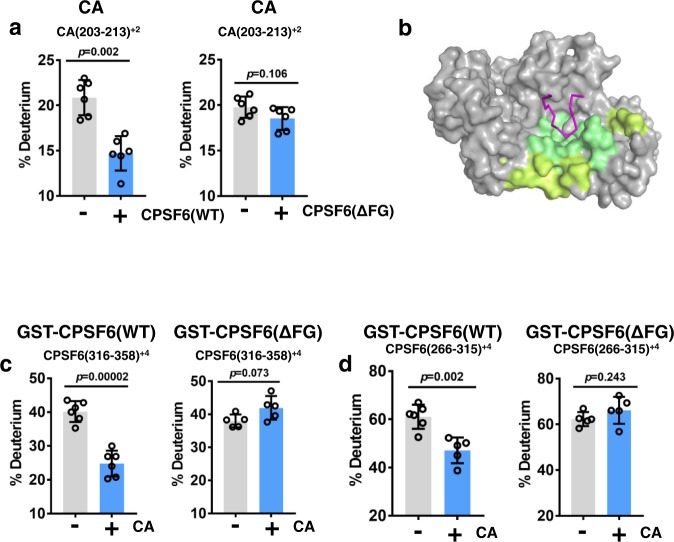
HDX-MS analysis of CPSF6(LCR-FG-LCR) binding to IP6-stabilized CA tubes. **a** The 10 s HDX results for the CA peptide (aa 203-213) that is specifically protected upon addition of WT GST-CPSF6_261-358_ but not GST-CPSF6_261-358_(ΔFG). Gray and cyan bars show HDX values for the indicated CA peptide in the absence vs presence of added GST-CPSF6. Consolidated results from differential HDX-MS for protection in CA are shown in Supplementary Fig. 14a). The averaged data (+/− SD) from six independent experiments are shown. Statistical analysis of samples was carried out with Student’s two-sample, two-tailed *t*-test. Source data are provided as a Source Data file (**b**) Our X-ray structure of the CA_hex_ + IP6 + CPSF6_313-327_ with protected CA regions colored according to the scale bar in Supplementary Fig. 14a and the regions showing no significant protection colored gray. For clarity, only one hydrophobic pocket formed by two adjoining CA monomers (colored gray) is shown. CPSF6 FG peptide is in magenta. The 10 s HDX results for CPSF6 peptides aa 316-358 containing the FG peptide + C-terminal LCR (**c**) and aa 266-315 containing N-terminal LCR (**d**) that are specifically protected in WT GST-CPSF6_261-358_ but not in GST-CPSF6_261-358_(ΔFG) upon incubation with IP6-stabilized CA(A92E) tubes. Gray and cyan bars show HDX values for the indicated CPSF6 peptides in the presence vs absence of IP6-stabilized CA(A92E) tubes, respectively. Consolidated results from differential HDX-MS comparing WT GST-CPSF6_261-358_ vs GST-CPSF6_261-358_(ΔFG) in the presence of IP6-stabilized CA(A92E) tubes are shown in Supplementary Fig. 14b. The averaged data (+/− SD) from six independent experiments are shown. Statistical analysis of samples was carried out with Student’s two-sample, two-tailed *t*-test Source data are provided as a Source Data file.

To further test the scenario suggested by our cryo-EM and AA MD simulation findings, we analyzed GST-CPSF6_261-358_(LCR-FG-LCR) interactions with IP6-stabilized CA tubes by HDX-MS (Fig. 6, Supplementary Fig. 14 and Supplementary Table 5), which is a powerful tool for probing protein-protein interactions^29^. Protections in CA are summarized in Supplementary Fig. 14a. The CA peptide fragments that exhibited statistically significant protection mapped to the FG peptide binding hydrophobic CA pocket and its immediate vicinity (Fig. 6a, b and Supplementary Fig. 14a). These protections are shown in the context of our X-ray structure of CA_hex_ + IP6 + CPSF6_313-327_ (Fig. 6b). No additional protections were observed at other CA regions. In control experiments with GST-CPSF6(ΔFG), no protection was observed in CA (Fig. 6a, Supplementary Fig. 14a). These findings support our cryo-EM and AA MD simulation studies indicating that GST-CPSF6_261-358_(LCR-FG-LCR) contacts to tubular CA lattices are limited to the FG peptide binding to the cognate hydrophobic CA pocket.

Protections in GST-CPSF6 are summarized in Supplementary Fig. 14b. Because of the unusual amino acid sequence of the CPSF6 LCR, pepsin hydrolysis yielded a limited number of CPSF6 peptides, albeit two large peptides, CPSF6(266-315) and CPSF6(316-358)_,_ provided near complete coverage of the CPSF6 LCR. Both CPSF6(266-315) and CPSF6(316-358) peptides exhibited statistically significant protection in the context of GST-CPSF6_261-358_(LCR-FG-LCR) + CA nanotubes but not with GST-CPSF6_261-358_(ΔFG) + CA nanotubes (Fig. 6c, d). Protection of the CPSF6(316-358) peptide (Fig. 6c), which includes the FG anchoring peptide and the C-terminal LCR, displayed the complementarity to protection seen in CA (Fig. 6a) consistent with the 15-mer FG peptide binding to the hydrophobic CA pocket (Supplementary Fig. 12)^6^. However, our HDX results did not allow us to discern whether the C-terminal LCR also contributed to the observed protection of the CPSF6(316-358) peptide.

Importantly, protection of the CPSF6(266-315) peptide in the context of GST-CPSF6_261-358_(LCR-FG-LCR) + CA nanotubes but not with GST-CPSF6_261-358_(ΔFG) + CA nanotubes (Fig. 6d) suggest a role of the N-terminal LCR in CPSF6-CPSF6 interactions. Specifically, these HDX-MS results are in excellent agreement with our cryo-EM and AA MD simulation findings showing that the N-terminal LCRs primarily mediate CPSF6-CPSF6 interactions (Supplementary Figs. 11b and 13d). The lack of HDX-MS protections in the GST fragment is also consistent with our cryo-EM and biochemistry experiments, which indicated that GST does not contribute to CPSF6-CA or CPSF6-CPSF6 interactions (Supplementary Fig. 14b).

Taken together, HDX-MS results reinforce our interpretation of the cryo-EM and AA MD simulation data indicating bimodal binding of CPSF6 to CA hexameric lattices: i) the CPSF6 FG peptide directly engages the hydrophobic CA pocket; and ii) the hexameric CA lattice promotes LCR-mediated CPSF6-CPSF6 interactions.

### Validation of the structural results

**Fig. 7:**
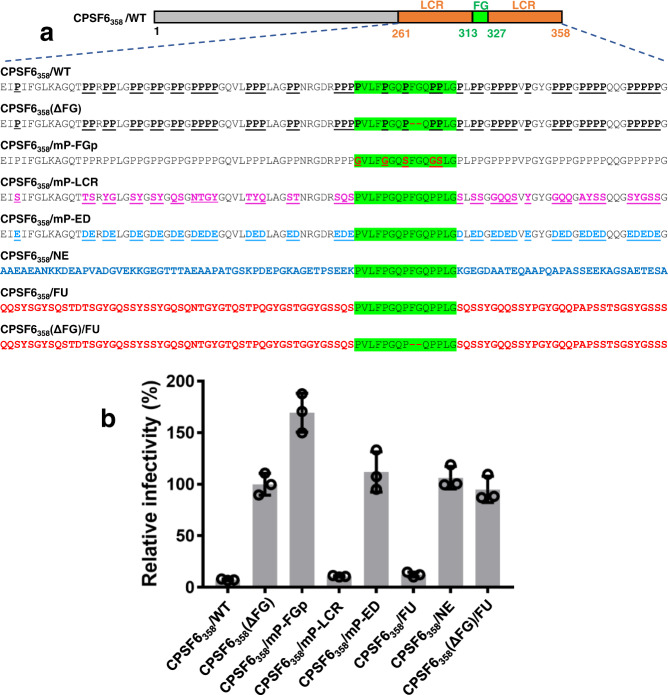
HIV-1 infectivity assays probing a role of Pro residues in the context of CPSF6(LCR-FG-LCR). **a** Schematic of WT and mutant CPSF6_1-358_ constructs. Amino acid sequences of the CPSF6(LCR-FG-LCR) segment targeted by mutagenesis are shown. CPSF6_358_/WT: native Pro residues are in bold and underlined. The FG containing peptide is highlighted in green. CPSF6_358_(ΔFG): FG residues were deleted. CPSF6_358_/mP-FGp: Pro residues in the FG-containing peptide were substituted with Gly or Ser. CPSF6_358_/mP-LCR: Pro residues in the LCR segments were substituted with non-charged residues. CPSF6_358_/mP-ED: Pro residues in the LCR segments were substituted with charged residues Glu or Asp. CPSF6_358_/NE: the LCR segments of CPSF6_358_ were replaced with non-LCR counterparts from NEURM_97-179_. CPSF6_358_/FU: the LCR segments of CPSF6_358_ were replaced with alternative LCR from FUS_35-117_. CPSF6_358_(ΔFG)/FU: FG residues were deleted from CPSF6_358_/FU. **b** Relative infectivity of VSV-G pseudotyped HIV-1 in the Hela cells stably overexpressing proteins shown in **a**. Infectivity was normalized to CPSF6_358_(ΔFG). The averaged data (+/− SD) from three independent experiments are shown. Source data are provided as a Source Data file.

**Fig. 8:**
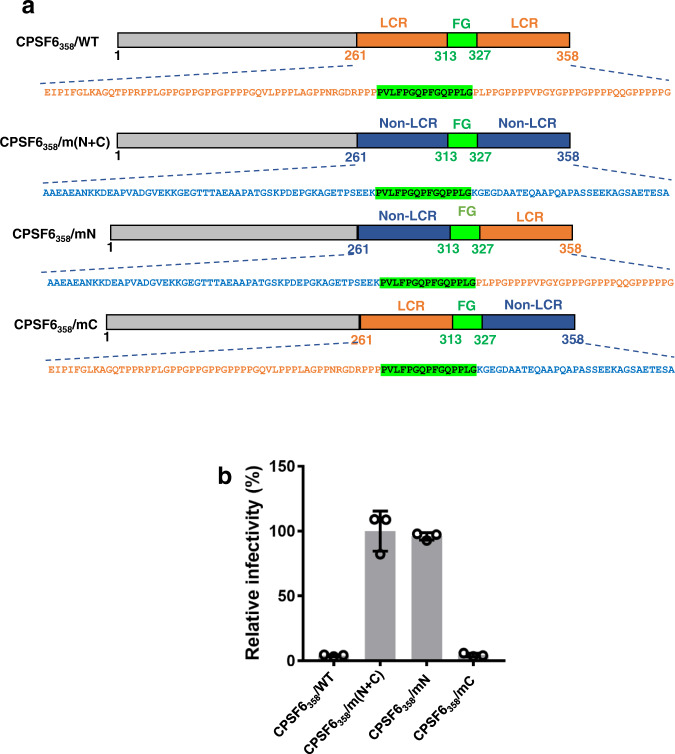
HIV-1 infectivity assays probing roles of N- and C-termini CPSF6 LCRs. **a** Schematic of WT CPSF6_358_ and corresponding mutant proteins stably overexpressed in Hela cells. CPSF6_358_/m(N + C): both N- and C-termini CPSF6 LCRs were replaced with non-LCR sequences from NEURM_97-179_. CPSF6_358_/mN: the N-terminal LCR was replaced with non-LCR sequences from NEURM_97-179_. CPSF6_358_/mC: the C-terminal LCR was replaced with non-LCR sequences from NEURM_97-179_. **b** Relative infectivity of VSV-G pseudotyped HIV-1 in the Hela cells stably overexpressing proteins shown in **a**. Infectivity was normalized to CPSF6_358_/m(N + C). The averaged data (+/− SD) from three independent experiments are shown. Source data are provided as a Source Data file. LCR low complexity region.

While the above biochemistry and virology results (Figs. 1–4) are in complete agreement with the structural findings (Figs. 5–6), we extended our virology experiments to further examine the significance of LCR-LCR interactions (Figs. 7 and 8, Supplementary Fig. 15). For this, we utilized C-terminal truncated CPSF6_1-358_ constructs. Due to the lack of the RS domain, considerable amounts of CPSF6_1-358_ are present in the cytoplasm and potently restrict HIV-1 nuclear import and infection^27,30,31^. This approach coupled with mutagenesis experiments enabled identification of CPSF6 FG residues essential for binding to HIV-1 CA^31^. However, roles of the CPSF6 regions implicated in LCR-LCR interactions by our cryo-EM and HDX-MS have not been analyzed previously.

We specifically asked whether LCR flexibility per se was sufficient or whether its ability to self-assemble was also required for functional CPSF6 interactions with HIV-1 cores. CPSF6(LCR-FG-LCR) contains ~49% Pro residues (Supplementary Fig. 1b). The structural results (Supplementary Fig. 12)^6^ suggest a key role of Pro residues for the 15-mer anchoring FG peptide to adopt a U-shape needed to effectively dock into the hydrophobic CA pocket. Indeed, the substitution of Pro residues within the 15-mer anchoring peptide was detrimental for CPSF6_1-358_’s ability to restrict HIV-1 infection (Fig. 7). However, contributions of Pro residues in the context of flanking LCRs are unknown. Accordingly, we substituted Pro residues in flanking LCRs with either charged (Glu and Asp, termed CPSF6_358_/mED) or uncharged, LCR-defining (Ala, Gly, Asn, Ser, Thr, Gln and Tyr, termed CPSF6_358_/mLCR) amino acids (Fig. 7a). While both constructs are expected to be unstructured or highly flexible, only CPSF6_358_/mED would preclude CPSF6-CPSF6 self-assembly seen in cryo-EM due to the charge repulsions. Strikingly, CPSF6_358_/mED completely failed to restrict HIV-1, whereas CPSF6_358_/mLCR retained WT CPSF6_1-358_ levels of HIV-1 restriction (Fig. 7b).

Consistent with these results, the following additional experiments with the chimeric proteins also indicated that LCR-LCR interactions rather than a high Pro content per se is important for functionally essential avid binding of CPSF6 to mature CA lattices (Fig. 7a, b). We substituted the LCRs with non-LCRs from NEURM (CPSF6_358_/NE) or alternative prion-like LCRs from FUS (CPSF6_358_/FU), which is known to self-assemble when an appropriate template is provided^32^. Both NEURM and FUS segments are highly flexible and contain usual levels of Pro (Supplementary Fig. 4a). Yet, the CPSF6_358_/NE chimeric protein was inactive, whereas CPSF6_358_/FU effectively restricted HIV-1 (Fig. 7b). Consistent with the requirement of hexameric CA lattices as a template for LCR-LCR assembly, deletion of the FG dipeptide from the 15-mer anchoring peptide CPSF6_358_(ΔFG)/FU failed to restrict HIV-1 infection. Collectively, these results further validate our structural findings of bimodal binding of CPSF6 to curved hexameric CA lattices with the U-shaped 15-mer FG peptide directly engaging with the hydrophobic CA pocket and flanking LCRs promoting CPSF6-CPSF6 interactions.

The next set of experiments focused on delineating the roles of the N- and C-terminal LCR sequences by substituting these regions with usual (nonLCR) amino acid sequences (Fig. 8). Consistent with the virology experiments with full-length CPSF6 constructs (Figs. 2–4), substitutions of both N- and C-terminal LCRs impaired the ability of CPSF6_1-358_ to restrict HIV-1 infection (Fig. 8). The substitution of the N-terminal LCR was detrimental, whereas the C-terminal LCR was dispensable for CPSF6_1-358_ interactions with HIV-1 in infected cells. These virology results are fully consistent with the cryo-EM and AA MD simulation findings, which revealed crucial roles of both the N-terminal LCR and 15-mer FG peptide for bimodal binding of CPSF6 to hexameric CA lattices.

### LEN interacts with HIV-1 cores at unoccupied hydrophobic CA pockets without displacing pre-bound CPSF6

While published studies^7,8^ indicated a multi-step, dose dependent inhibition of early steps of HIV-1 infection by LEN, the primary antiviral mode of action of the inhibitor is not clear. Two distinct mechanisms have been proposed for the ability of LEN and its very close analog GS-CA1 to potently inhibit HIV-1 ingress: i) outcompete the FG containing cellular cofactors from HIV-1 cores, and ii) compromise functional flexibility of the HIV-1 CA lattice^7,8,33,34^. Link et al. suggested that high potency of the inhibitor is due to the block of the nuclear import of viral cDNA via direct competition with host-cell nuclear import cofactors NUP153 and CPSF6^7^. However, we noticed that effective block of nuclear import required 5 nM LEN, whereas 0.5 nM inhibitor only partly impaired nuclear import of viral cDNA, yet fully inhibited integration^8^ suggesting that LEN could be most active post-nuclear entry. These discrepancies between published reports^7,8^ prompted us to delineate the primary mode of LEN antiviral action.

**Fig. 9:**
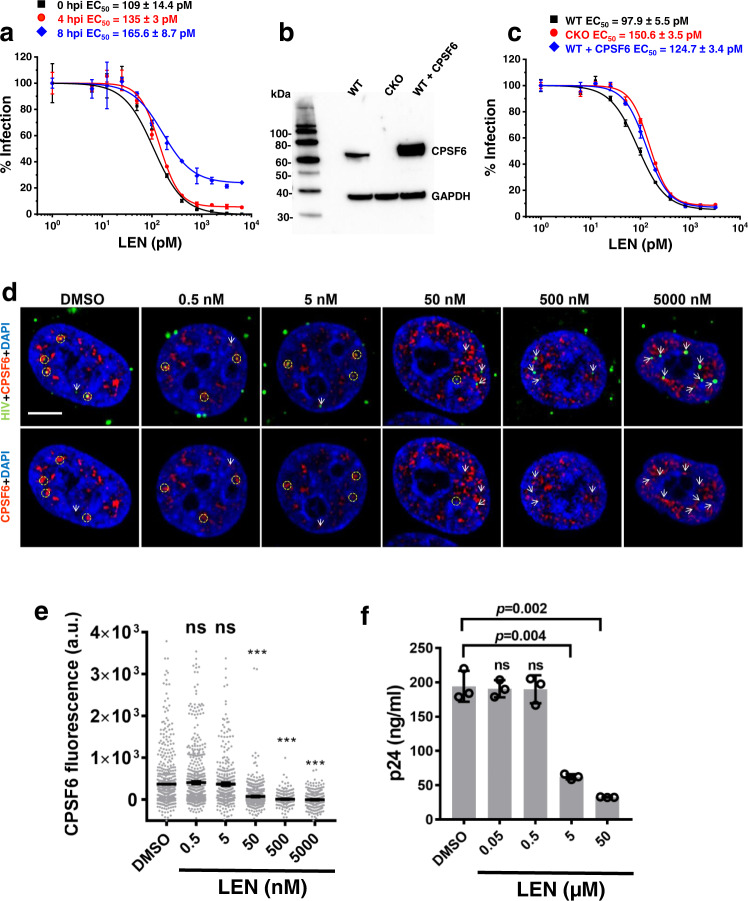
Interplay between LEN, CPSF6 and HIV-1. **a** Antiviral activities after LEN addition to HEK293T cells at indicated time points post-infection. The normalized and averaged data (+/− SD) from three independent experiments are shown. Source data are provided as a Source Data file. **b** Representative immunoblotting to show the expression levels of CPSF6 in parental, CPSF6 KO (CKO) and CPSF6 overexpressing HEK293T cells. The experiment was repeated 3 times independently with similar results. **c** Antiviral activities of LEN in indicated cells with varying levels of CPSF6. VSV-G pseudotyped HIV-scarlet viruses (MOI = 0.5) were used to infect indicated cells. The infection levels in different cell lines without LEN treatment were 43.9 ± 2% (WT), 38.5 ± 0.9% (CKO) and 38.4 ± 1.6% (WT + CPSF6). The normalized and averaged data (+/− SD) from three independent experiments are shown in **c**. Source data are provided as a Source Data file. **d** TZM-bl cells were infected with INmNG-labeled fluorescent HIV-1 pseudoviruses for 8 h to allow virus nuclear import^40^. At 8 hpi, cells were treated with DMSO or indicated concentrations of LEN for 30 min, fixed with PFA and immuno-stained for endogenous CPSF6. Single Z-slice images show nuclear IN-puncta (green) and endogenous CPSF6 (red). Yellow dashed circles and white arrows point to nuclear IN-puncta colocalized or not-colocalized with CPSF6, respectively. Insignificant nuclear import occurs within 30 min of LEN treatment. Scale bar is 5 μm. **e** Analysis of background subtracted CPSF6 fluorescence associated with nuclear IN puncta in **d**. All data points (*n* = 553 for DMSO; *n* = 740 for 0.5 nM LEN; *n* = 389 for 5 nM LEN; *n* = 746 for 50 nM LEN; *n* = 309 for 500 nM LEN; *n* = 412 for 5000 nM LEN) obtained from a total of three independent experiments are overlayed. Statistical significance of comparison of indicated concentration of LEN versus DMSO control was determined by non-parametric Mann-Whitney two-tailed rank sum test. *P*-values for 0.5, 5, 50, 500 and 5000 nM LEN were 0.21, 0.66, 1.3E-24, 1.7E-22, 2.5E-28, respectively. ***p < 0.0001. ns: not significant. Source data are provided as a Source Data file. **f** Isolated HIV-1 cores were incubated with 2 μM GST-CPSF6_261-358_ for 20 min. DMSO control or indicated concentrations of LEN were added to the mixture and incubated for 20 min. The HIV-1 cores pulled-down by GST-CPSF6_261-358_ through glutathione sepharose beads were detected by ELISA. The averaged data (+/− SD) from three independent experiments are shown. Source data are provided as a Source Data file. LEN lenacapavir, a.u. arbitrary units.

To test the ability of LEN to block HIV-1 within the nucleus, we compared antiviral activities upon addition of the inhibitor prior to infection (0 h post-infection; hpi) vs after HIV-1 nuclear import (4 and 8 hpi). Similar, pM levels of EC_50_ values observed for these different time points (Fig. 9a) indicated that LEN is highly potent post-nuclear entry where it encounters CPSF6-HIV-1 core complexes. Therefore, follow up experiments investigated the interplay between LEN, CPSF6 and HIV-1 in the nucleus.

We checked how varying cellular levels of CPSF6 affected LEN EC_50_ values by comparing inhibitor activities in WT, CPSF6 KO, and CPSF6-overexpressing HEK293T cells (Fig. 9b, c). The results in Fig. 9c indicated that LEN exhibited comparable EC_50_ values across these cell lines, which suggested the following possibilities: i) LEN readily displaces CPSF6 from HIV-1 cores; or ii) LEN can interact with HIV-1 cores at unoccupied hydrophobic CA pockets without displacing pre-bound CPSF6 and thereby inhibit infection. To test these possibilities, we allowed CPSF6 to engage with HIV-1 in the nucleus during 8 h of infection, and then added varying concentrations of LEN (Fig. 9d, e).

Strikingly, LEN concentrations that markedly (>1,000-fold) exceeded its antiviral EC_50_ values (~165 pM) were needed to disrupt CPSF6-HIV-1 complexes in the nucleus of infected cells (compare Fig. 9a, e). Complementary biochemical assays yielded similar results (Fig. 9f). While LEN bound to CA hexamers with *K*_*d*_ of ~ 240 pM^7^, much (>1,000-fold) higher inhibitor concentrations were required to displace GST-CPSF6_261-358_ from isolated HIV-1 cores (Fig. 9f). Additional biochemical experiments examined interactions of LEN and GST-CPSF6_261-358_ with preformed CA nanotubes (Supplementary Fig. 16c, d). In the presence of 1.5 M NaCl, where CA nanotubes were formed and remained stable, addition of LEN did not displace pre-bound GST-CPSF6_261-358_ (compare lanes 4 with 2 in Supplementary Fig. 16c). Upon subsequent exposure of these complexes, which were pre-formed in the high ionic strength buffer (Supplementary Fig. 16c), to 0.15 mM NaCl-containing buffer lacking IP6 resulted in marked dissociation of CA nanotubes in the absence and presence of GST-CPSF6_261-358_ (lanes 1 and 2, Supplementary Fig. 16d). In sharp contrast, LEN strikingly stabilized CA nanotubes as well as the CA + GST-CPSF6_261-358_ complex without displacing the cellular cofactor (lanes 3 and 4, Supplementary Fig. 16d). Taken together, these findings indicate that the primary antiviral activity of LEN is not through displacing CPSF6 from HIV-1 cores. Instead, the inhibitor hyper-stabilizes the CA lattice despite the presence of bound CPSF6. The requirement for very high LEN concentrations to outcompete CPSF6 from HIV-1 cores supports our principal findings that CPSF6 is tightly bound to the mature CA lattice in the nucleus.

How can pharmacologically relevant concentrations of LEN gain access to cognate hydrophobic CA pockets in the context of CPSF6 bound to HIV-1 cores? The answer to this question is provided by our cryo-EM and biochemical studies (Fig. 5 and Supplementary Fig. 16). In contrast to our X-ray crystal structure (Supplementary Fig. 12a), which shows six CPSF6 FG peptides bound to six hydrophobic pockets in an isolated CA hexamer, our cryo-EM studies reveal the differential, sub-stoichiometric binding of CPSF6(LCR-FG-LCR) molecules to extended CA lattices (Fig. 5). Furthermore, our biochemical assays determined a stoichiometry of ~2.4 GST-CPSF6(LCR-FG-LCR) molecules bound to each CA hexamer in the context of tubular CA assemblies (Supplementary Fig. 16a, b). Taken together, our results indicate that LCR-LCR interactions (Fig. 5h, Supplementary Figs. 11 and 13), which are lacking from the substantially shorter CPSF6 FG peptide used in the X-ray crystallography experiments (Supplementary Fig. 12), dictate the observed pattern and stoichiometry of CPSF6(LCR-FG-LCR) binding to extended hexameric CA lattices. In turn, our cryo-EM findings reveal that even upon avid, multivalent assembly of CPSF6 on hexameric CA lattices, four hydrophobic pockets per each CA hexamer remain readily accessible for LEN binding. Thus, LEN does not have to outcompete tightly bound CPSF6 from HIV-1 cores. Instead, the inhibitor effectively engages with unoccupied hydrophobic CA sites and allosterically modulates the mature CA lattice. These structural observations are fully consistent with the virology results (Fig. 9a–e), which clearly delineate that LEN can potently and effectively inhibit HIV-1 inside the nucleus of infected cells without outcompeting CPSF6 from HIV-1 cores.

## Discussion

Our virology, biochemistry and structural biology studies have collectively elucidated the previously unknown mechanism for prion-like LCR mediated avid virus-host interactions during HIV-1 infection. We show that, unlike low affinity interactions of the respective FG peptides to isolated CA hexamers^6^, CPSF6, NUP153 and SEC24C avidly bind to the biologically relevant mature CA lattice (Fig. 1). These interactions require not only the FG peptide but prion-like LCRs that are present in each of these proteins. Our cryo-EM and HDX-MS studies reveal that prion-like LCR-LCR interactions enable polyvalent assembly of CPSF6 onto hexameric CA lattices. In turn, CPSF6-CPSF6 interactions are templated by binding of the embedded FG peptides to a subset of cognate hydrophobic CA pockets positioned along adjoining hexamers (Fig. 5 and Supplementary Fig. 11). This notion is supported by the observations that CPSF6(ΔFG) failed to induce CPSF6-CPSF6 interactions in vitro in the presence of the mature CA lattice (Fig. 6 and Supplementary Fig. 14) or in infected cells^4^.

The bimodal mechanism of CPSF6 binding to HIV-1 cores is conceptually reminiscent of TRIM-5α binding to hexameric CA lattices, which is mediated by i) a very low binding affinity of the SPRY domain to CA, and ii) TRIM5α-TRIM5α interactions^35^ that markedly enhance avidity for these virus-host interactions. Clearly, the structural details of CPSF6 and TRIM5-α interactions with HIV-1 cores diverge substantially because i) CPSF6 FG peptide and as yet to be identified TRIM5a SPRY binding sites on CA are likely to be different; ii) TRIM5α-TRIM5α assemblies are mediated by B-box 2 and coiled-coil interactions resulting in a TRIM5α hexagonal cage surrounding the hexameric CA lattices^35^, whereas LCR-LCR interactions enable multivalent CPSF6 assembly in zig-zagging lines extending between adjoining CA hexamers (Fig. 5f, h and Supplementary Fig. 11b). While our cryo-EM experiments have been performed with tubular CA assemblies and GST-CPSF6_261-358_, our virology studies have validated an essential role of the LCR in the context of full-length CPSF6 interactions with HIV-1 cores in infected cells (Figs. 2–4 and Supplementary Fig. 7). We also note close structural similarities between curved hexameric CA lattices in the context of tubular assemblies versus native conical capsid^9,10^. Therefore, it is logical to propose that the LCR-LCR interactions, which exhibit high conformational flexibility (Supplementary Fig. 13), mediate avid, polyvalent assembly of CPSF6 molecules onto the conical capsid during early steps of HIV-1 replication.

While previous reports referred to CPSF6_261-358_ as a Pro rich region, Pro content varies substantially in CPSF6_261-358_ (~49 %), NUP153_1306-1450_ (~14 %) and SEC24C_196-314_ (~25 %). Instead, we noticed that the common feature of these three CA binding cellular proteins is that the FG peptides are embedded in prion-like LCRs, which are characterized by strong bias for uncharged residues and respective scarcity of charged residues (Supplementary Fig. 1). All three CPSF6_261-358_, NUP153_1306-1450_ and SEC24C_196-314_ protein segments required respective LCRs for high affinity binding to HIV-1 cores (Fig. 1). Furthermore, our detailed structural and mechanistic studies with CPSF6 clarified the importance of LCR-LCR interactions rather than the Pro content per se for the functional virus-host interactions. Since SEC24C, NUP153 and CPSF6 are predominantly present in the cytoplasm, the NPC and the nucleus, respectively, we suggest that the avid prion-like LCR mediated virus-host interactions are utilized throughout early steps of infection to enable HIV-1 to effectively traffic across the cytoplasm, transit the NPC, and complete the journey inside the nucleus to integrate into chromatin.

Although roles of prion-like LCRs have been extensively studied in the context of neurodegenerative diseases^14^, it is increasingly clear that such interactions are needed for normal functions of LCR containing proteins. For example, prion-like properties enable NUPs, including NUP153, to self-assemble within the nuclear pore-complex (NPC)^13,15^. Unlike NUPs, CPSF6 is broadly distributed within the nucleus of uninfected cells (Supplementary Fig. 7). Instead, CPSF6 strongly accumulates around HIV-1 cores in the nuclei of infected cells (Supplementary Fig. 7)^4,26,27^. In excellent agreement with these observations, our cryo-EM and HDX-MS studies revealed that the curved hexameric CA lattice templated multivalent assembly of GST-CPSF6_261-358_(LCR-FG-LCR), whereas no self-assembly was observed with purified GST-CPSF6_261-358_(LCR-FG-LCR) alone. Furthermore, our structural and virology findings are in agreement with recent reports^36,37^ indicating that viral cores are imported inside the nucleus. The experiments with chimeric proteins demonstrate a crucial role of CPSF6 LCR for binding to and co-trafficking with HIV-1 cores within the nuclear interior to preferred sites of integration into SPADs (Figs. 2–4). To accomplish these functions, the CPSF6 LCR would require a template consisting of large hexameric CA lattices rather than isolated CA hexamers (Figs. 1 and 5).

Our characterization of CPSF6 interactions with HIV-1 CA lattices provided unexpected, important insight into the highly potent antiviral activity of LEN. While the crystal structures have suggested mutually exclusive binding of short FG peptides vs LENs to each of six hydrophobic pockets in an isolated CA hexamer^7,8^ (Supplementary Fig. 12), our cryo-EM and biochemistry experiments reveal that CPSF6(LCR-FG-LCR) molecules engage only a subset of hydrophobic sites in the context of biologically relevant, extended HIV-1 CA lattices (Fig. 5g). Accordingly, LEN could readily access the unoccupied hydrophobic CA pockets in the presence of bound CPSF6 and hyper-stabilize the CA lattice (Supplementary Fig. 16). Consistent with these structural and biochemical observations, the virology experiments in Fig. 9 show that the inhibitor can potently and effectively impair HIV-1 inside the nucleus without outcompeting tightly bound CPSF6 from virus cores. Conversely, LEN is markedly (>1,000-fold) less effective at displacing CPSF6 from HIV-1 cores (Fig. 9). Taken together, our virology, biochemistry and structural biology experiments delineate that the primary antiviral activity of LEN does not rely on displacing CPSF6 from HIV-1 cores and instead, the inhibitor functionally compromises necessary pliability of the CA lattice (Supplementary Fig. 16)^8,34^. Follow up studies are warranted to characterize the interplay between LEN and other FG motif containing proteins such as NUP153 and SEC24C with HIV-1 cores to elucidate additional, albeit less potent antiviral activities of this multimodal inhibitor during nuclear import and cytoplasmic trafficking. In addition, our findings reported here about the primary mode of action of LEN will inform future efforts of rationally developing improved LEN analogs.

## Methods

### Cells

Parental HEK293T (ATCC, CRL-3216), CPSF6 knockout HEK293T (CKO)^5^, PhoenixAMPHO (ATCC, CRL-3213), TZM-BL cells (NIH AIDS Reagent Program, 8129) and HeLa cells (ATCC, CCL-2) were cultured in Dulbecco’s modified eagle medium (DMEM, Gibco) complemented with 10% fetal bovine serum (FBS, Sigma-Aldrich) and 1% penicillin-Streptomycin (PS, Gibco) at 37 °C and 5% CO_2_. HeLa cells stably expressing WT CPSF6_1-358_ and corresponding chimeric proteins were transduced by lentiviral vectors. CKO cells stably expressing full-length CPSF6 and corresponding chimeric proteins were transduced with gammaretroviral vectors. Transduced cell lines were selected and cultured in DMEM complemented with 10% FBS, 1% PS and 2 μg/mL puromycin (Millipore Sigma). All cell lines used in the present study were tested monthly using Mycoplasma polymerase chain reaction (PCR) detection kit (Genlantis) and there has been no evidence of Mycoplasma contamination.

### Plasmids

For ectopic expression of CPSF6_1-358_ and corresponding chimeric proteins the coding sequences were engineered in the Tsin-IRESpuro plasmid, a gift from Dr. Eric Poeschla^38^. The pLPCX and pMX plasmids^31^ were used for production of gammaretroviral vectors. For ectopic expression of full length CPSF6 and corresponding chimeric proteins, the coding sequences were inserted into pLPCX plasmid. The pHIVeGFP and Vpr-INsfGFP plasmids^39,40^ were used for immuno-fluorescence experiments. Sequences of primers used for cloning are shown in Supplementary Table 6. Nanodrop-1000 (v3.8.1) was used to analyze DNA concentrations.

### Virus production

The luciferase-encoding pseudotyped HIV-1 viruses were produced by co-transfecting VSV-G and pNL4-3-E-R-Luc plasmids in HEK293T cells. The lentiviruses for transduction were produced by co-transfecting VSV-G, Δ8.2 and Tsin-IRESpuro based plasmids in HEK293T cells. The gammaretroviral vectors were produced by co-transfecting VSV-G, PJK3, pL-VSVG and pCMV-tat in PhoenixAMPHO cells^1^. Fluorescently labeled pseudotyped viruses were produced by transfection with HIV-1 pR9ΔEnv, VSV-G and Vpr-INmNG in HEK293T cells as described^4^.

### Immunoblotting

Cells were lysed with RIPA buffer, separated with Bolt™ 4-12% Bis-Tris Plus Gels (Invitrogen) and transferred to nitrocellulose membranes. The membranes were blocked with 5% blotting-Grade Blocker (Cat # 1706404, Bio-RAD) using 1xTBST buffer (Cat # T9511, Teknova) for 1 h and incubated overnight at 4 °C with following primary antibodies: anti-HA antibody (1:1000, ab236632, Abcam), anti-GST antibody (1:1000, Cat # 8-326, Thermofisher), anti-CPSF6 antibody (1:5000, ab175237, Abcam), anti-Sec24C antibody (1:1000, ab122633, Abcam), anti-Nup153 antibody (1:2000, NB100-93329, Novus), anti-GAPDH antibody (1:3000, sc-47724, Santa Cruz). The membranes were subsequently incubated with a goat anti-rabbit IgG (H + L) secondary antibody (1:3000, 65-6120, Invitrogen) or goat anti-mouse IgG (H + L) secondary antibody conjugated (1:3000, 65-6520, Invitrogen) to horseradish peroxidase and visualized by enhanced chemi-luminescence (RPN2232, Cytiva).

### Immunofluorescence

For imaging of uninfected HeLa or HEK293T cells, they were fixed with 4% paraformaldehyde and permeabilized with 0.1% Triton X-100 in PBS for 15 min. Unspecific binding was blocked with 3% bovine serum albumin (BSA) for 30 min followed by incubation with primary antibodies: anti HA antibody (1:1000, ab236632, Abcam). The cells were washed with 0.1% Tween-20 in PBS and incubated with secondary goat anti-rabbit Alexa Fluor 594 antibodies (1:1000, Cat # A32740, Invitrogen). Nuclei were counter-stained with 4,6-dia-midino-2-phenylindole (DAPI) (1:5000, Cat # 62248, Thermofisher). Cells were observed using an Olympus FV1000 laser scanning confocal microscope in the Advanced Light Microscopy Core part of NeuroTechnology Center at University of Colorado Anschutz Medical Campus.

For imaging of the cells infected by GFP-labelled HIV-1, 5 × 10^4^ CKO-cells expressing HA-tagged CPSF6 proteins were infected in an 8-well chambered coverslip at MOI 0.5 with INsfGFP labeled fluorescent HIV-1 pseudoviruses by spinoculation. After spinning at 1,450 g for 30 min at 16 °C, cells were washed once with dPBS (Ca + /Mg + ) and incubated at 37 °C in 5% CO_2_ for 4 h. Cells were fixed with 2% PFA (Electron Microscopy Sciences, #1570-S) for 7 min at room temperature, permeabilized with 0.5% Triton X-100 and immune-stained in blocking solution (3% BSA with 0.1% Tween-20 in PBS). Primary anti-SON antibody (1:1000, HPA031755, Atlas antibodies) and anti-HA antibody (1:1000, ab236632, Abcam) diluted in a blocking solution were allowed to bind for 1 h at room temperature or overnight at 4 °C. Cells were washed 5 times with PBST and incubated with secondary goat anti-mouse Cy5-conjugated secondary antibodies (1:1000), washed five times, and incubated with goat anti-rabbit-AlexaFluor405 (1:1000), each for 1 h at room temperature. Following five washes in PBST the cells were stained for the nuclei by SiR-Hoechst (Cytoskeleton, Inc., cat#: SC007) (1:1000) for 5 min, washed three times with PBST, and imaged in dPBS. Confocal imaging was performed using a 63x/1.4NA oil objective on a Zeiss LSM880 confocal microscope. Z-stack images spaced at 0.5 µm were collected by using Carl Zeiss MicroImaging Zen software Black Version (2.3 SP1) from four fields of view using the 405, 488, 561, and 633 nm laser lines, and respective emissions for AF405 (415-470 nm), sfGFP (502-550 nm), AF561 (572-640 nm) and SiR-Hoechst (640-720 nm) were collected using GasP-detectors. Images were analyzed offline using ICY image analysis software (http://icy.bioimageanalysis.org/). Nuclear INsfGFP puncta colocalized with the SON-nuclear speckles was determined in 3D datasets as described previously^4^. For detection of HIV-1 infection-induced CPSF6 aggregation, the target cells were plated on 12 mm coverslips (72230-01, Electron Microscopy Sciences) in 24 well tissue culture plates. Samples were fixed with 4% paraformaldehyde (PFA, Boston Bioproducts) at room temperature for 10 min, permeabilized by 0.5% Triton X-100 at room temperature for 10 min, and then blocked with 3% BSA at room temperature for 30 min. Cells were incubated with primary antibodies at room temperature for 1 h, followed by Alexa-Fluor-conjugated secondary antibodies and Hoechst 33342 (Thermo Fisher Scientific) at room temperature for 30 min. Coverslips were mounted on glass slides with fluorogel (17985-10, Electron Microscopy Sciences). Primary antibodies against HA-tag (NB600-362, Novus bio) (1:200) and Flag-tag (F1804, Sigma) (1:1000) were used. Goat anti-rabbit Alexa Fluor 488 (A11070, Invitrogen) and goat anti-mouse Alexa Fluor 647 (A21235, Invitrogen) were used as secondary antibodies.

### Fluorescence in situ hybridization (FISH)

To detect HIV-1 DNA a pool of 48 probes labeled with Atto 565 dye (Biomers) were used for targeting the HIV-1 integrase coding region. HeLa cells were infected with HIV-1 at MOI 50. After 6 h post synchronized infection the cells were fixed by 4% paraformaldehyde (PFA) and permeabilizated by 0.5% Triton X-100. Then samples were treated with RNase A (0.1 mg/ml, Qiagen) at 37 °C for 1 hour. For deproteinization, samples were treated with 0.1 N HCl at room temperature (RT) for 10 min. After deproteinization, samples were equilibrated using 2× Saline-Sodium Citrate buffer (SSC) and incubated with pre-warmed 2×SSC at 70 °C for 30 min. Samples were then gradually cool down to 37 °C from 70 °C. After removal of the 2×SSC, the samples were incubated with 0.1×SSC at room temperature for 1 min followed by incubation in 0.07 N NaOH at room temperature for 1 min. After removal of 0.07 N NaOH, the samples were incubated with ice-cold 0.1×SSC at 4 °C for 1 min and ice-cold 2×SSC at 4 °C for 1 min. Then dehydration steps were performed using 70%, 85% and 100% ethanol at room temperature for 1 min. HIV-1 DNA probe with 20 mM/coverslip (in hybridization buffer) was added and incubated at 37 °C for 16 h. Subsequently the samples were washed with 2×SSC at 37 °C for 30 min, 2×SSC at room temperature for 30 min and then 1×SSC at room temperature for 30 min. The samples were rinsed with PBS and then washed 3 times with PBS. Then Hoechst staining was performed at room temperature for 5 min. Following washes with PBS, the samples were mounted with fluorogel (Electron Microscopy Sciences).

### PLA

PLA experiments were conducted as described^1^. HEK293T cells were seeded on coverslips in a 24-well dish. The cells were challenged with VSV-G pseudotyped HIV-1 (500 ng of p24), washed and supplied with fresh medium at 1 hpi. The cells were fixed with 4% paraformaldehyde for 15 min at 6 hpi and permeabilized with 0.1% Triton X-100 for 15 min at room temperature. After washing with a blocking buffer supplied by Duolink In Situ Red kit (DUO92101, Sigma–Aldrich) for 1 h at 37 °C, the cells were incubated with anti-HIV-1 p24 antibody AG 3.0 (ARP-4121, NIH AIDS Reagent Program) at 1:100 dilution and anti-HA antibody (ab236632, Abcam) (1:1000) for 1 h at room temperature. Samples were processed further using the Duolink In Situ Red kit according to the manufacturer’s instructions. Signals were detected by using an Olympus FV1000 laser scanning confocal microscope.

### Single-cycle infection assay

5 × 10^4^ cells/well were seeded in a 24-well dish and infected with 10 ng of p24 VSV-G pseudotyped HIV-1 virions. Supernatants were removed and cells were washed with PBS once after 1 hpi. Fresh complete medium was added and the cells were cultured further for 48 h. The cells were lysed with the reporter lysis buffer (Cat.# E1531, Promega) and centrifuged to remove the cell debris. Luciferase activity in cellar extracts were determined using Luciferase Assay System (Cat.# E1531, Promega).

### Sequencing of HIV-1 integration sites

Integration libraries were prepared using ligation-mediated PCR (LM-PCR) as described^41,42^. Genomic DNA (2-10 µg) from HIV-1 infected cells was digested with restriction enzymes Mse I and Bgl II overnight at 37 °C. Digested and purified DNA fragments were ligated to double-stranded DNA linkers containing 5’-TA overhangs overnight at 12 °C. Purified ligation products were used in nested PCRs to amplify viral-host integration junctions for downstream sequencing. First round PCR primers were designed to amplify sequences between the U5 end of HIV-1 DNA and the linker. Second round PCRs contained a nested U5-specific primer with the same linker-specific primer; both of these were megaprimers containing sequences required for clustering during Illumina sequencing. Purified PCR products were subjected to 150 bp paired-end Illumina sequencing at Genewiz.

Illumina raw reads were processed, and integration sites were determined, as per previously described methodologies^25,43^. U5 and linker specific sequences were trimmed from Illumina read1 and read2, respectively. Trimmed reads, which contained host DNA, were aligned to human genome build hg19 by BWA-MEM aligner with paired-end option^44^. The hg19 genome was obtained from the UCSC (http://genome.ucsc.edu). Aligned reads were filtered to remove unmapped and low quality score mapped reads, as well as reads that mapped to more than one region of hg19, using SAMtools^45^ as described^43^. Reads with <900 bp between integration and linker ligation sites were selected and converted into BED format as described^25^. Integration sites were analyzed by BEDtools (commands intersect and window) to assess the distribution of integration sites with various genomic features^46^. The coordinates of speckle-associated domains (SPADs), lamina-associated domains (LADs) and random integration control (RIC) were used from published studies^4,24,25^. Statistical significance was determined by Student’s two-sample, two-tailed *t*-test or Fisher’s exact test except in the case of gene-density, where we used the Wilcoxon rank sum test. Illumina raw sequences for integration sites are available at the National Center for Biotechnology Sequences Read Archive with accession number PRJNA787708.

### Preparation of purified recombinant proteins

WT CA, CA(A92E), CA_A14C/E45C/W184A/M185A_ and 6-His-CA_A14C/E45C/W184A/M185A_ were expressed from pET3a in BL21-DE3 cells and purified as previously described^47–49^ through two column chromatography using HiTrap SP-Sepharose High Performance and HiTrap Q-Sepharose High Performance 5 ml columns (GE Healthcare) for hexamer formation. The inter-subunit disulfide-stabilized CA hexamers (CA_hex_) were assembled as previously published^48^. Assembled hexamers were purified further through size exclusion chromatography using a GE Healthcare HiLoad 16/600 Superdex 200 pg column with a buffer consisting of 20 mM Tris-HCl, pH 8.0 and 150 mM NaCl. CA_hex_ and His-CA_hex_ were detected using non-reducing SDS–PAGE. CA_hex_ was concentrated to ~16 mg/ml with a 50 kDa cutoff Amicon Ultra-15 Centrifugal concentrator for utilization in crystallization. 6His-CA_hex_ was concentrated to 2-7 mg/ml with a 50 kDa cutoff Amicon Ultra-15 Centrifugal concentrator for utilization in surface plasmon resonance experiments.

For expression of recombinant WT and mutant GST-CPSF6, GST-NUP153 and GST-SEC24C protein segments, the coding sequences of indicated protein constructs were engineered in the pEX plasmid^1^. The recombinant proteins were expressed in *E. coli* and purified through two column chromatography steps using 5 ml GSTrap 4B and HiTrap Q High Performance 5 ml columns (GE Healthcare). Quickchange II XL site-directed mutagenesis kit (Agilent) for preparation of mutant proteins. Protein concentrations were analyzed by using Enspire manager.

### Interactions of the recombinant CPSF6, NUP153 and SEC24C protein segments with isolated, native HIV-1 cores

Native HIV-1 cores were isolated as described^50^. Briefly, the luciferase-encoding VSV-G pseudotyped HIV-1 viruses were produced in HEK293T cells using nine 15 cm-dishes. Virions were pelleted through 20% sucrose at 32,000 rpm for 2 h at 4 °C. The pellet was incubated with 1xSTE buffer (10 mM Tris-HCl [pH 7.4], 100 mM NaCl, 1 mM EDTA) at 4 °C for 3 h and separated through a 12 ml linear 30%-70% sucrose gradient at 32,000 rpm overnight at 4 °C. The fraction containing isolated HIV-1 cores were identified through immunoblotting using recombinant anti-HIV-1 p24 antibody (ab32352, Abcam). HIV-1 cores were quantified by p24 ELISA (Cat # 0801111 Zeptometrix) and stored at −80 °C until use.

Binding of recombinant proteins to isolated HIV-1 cores were monitored as described^1^. The GST-tagged recombinant proteins and isolated HIV-1 cores were incubated for 20 min at 4 °C. The complexes were captured with 20 μl pre-equilibrated glutathione sepharose 4B beads (Cat # 17075601 GE Healthcare). Unbound proteins were washed away from the beads with 0.1% tween in the STE buffer. The bound proteins were boiled in 1% SDS for 5 min and analyzed by p24 ELISA. Origin 2019 (v.9.6) software was used to determine the K_d_ values based on p24 levels in the bound fractions.

### Interactions of recombinant CPSF6, NUP153 and SEC24C protein segments with CA nanotubes

WT CA nanotubes were assembled by incubating 78 μM CA in a buffer containing 25 mM Tris-HCl, pH 7.5 and 2 M NaCl at room temperature. Indicated amounts of tested recombinant proteins or cellular lysates were added to the preformed CA tubes and incubated for 30 min at 4 °C. The mixtures were centrifuged at 21,000 *g* for 5 min at 4 °C. Supernatants were discarded, pellets were washed three times, analyzed by SDS-PAGE and visualized by AcquaStain.

### Surface plasmon resonance

Surface plasmon resonance biosensor binding experiments were performed using the Reichert 4-SPR. A nitrilotriacetic acid (NTA) sensor chip was conditioned with 40 mM NiSO_4_ at a flow rate of 25 μl/min for 3 min. Cross-linked His-CA_hex_ was immobilized on the NTA sensor chip via the C-terminal His-tag. The running buffer contained 0.01 M HEPES pH 7.4, 0.15 M NaCl, 0.05% v/v Surfactant P20. CPSF6_313–327_, NUP153_1409-1423_, and SEC24C_228–242_ peptides were synthesized by Biomatik. All three peptides were prepared by serial dilution in the running buffer for concentrations ranging between 3.9 μM and 2 mM. The sensor chip was regenerated with 350 mM EDTA and 50 mM NaOH. For each interaction, background binding and drift were subtracted via a reference surface. Data was analyzed using Scrubber 2.0 and fit with a simple kinetic model with a term for mass transport when necessary.

### Cryo-EM

For analysis of HIV-1 CA tubes + GST-CPSF6_261-358_, we used CA(A92E) (3 mg/ml, corresponding to ~117 µM) to assemble tubes at room temperature overnight in 50 mM Tris, pH 7.5 containing 1 M NaCl and 200 µM IP6. The tubes were dialyzed for 1 h into 50 mM Tris, pH 7.5 containing 150 mM NaCl and 200 µM IP6. Recombinant GST-CPSF6_261-358_ was added by gentle mixing to the assembled tubes to a ~1:1 CA:GST-CPSF6_261-358_ molar ratio. The mixture was diluted 3-fold and immediately used to prepare cryo-EM samples. A 2.5 µl volume of sample was applied onto each lacey carbon grid (Ted Pella) that had previously been plasma-cleaned for 6 s on a Solarus plasma cleaner (Gatan) using an Ar/O_2_ gas mixture. The samples were vitrified in liquid ethane using a Vitrobot Mark IV (ThermoFisher) blotting for 6 s with −6 blotting force at 4 °C and 100% relative humidity. After vitrification, samples were stored in liquid nitrogen until needed for cryo-EM imaging. A total of 4706 direct electron detector (DED) frame stacks were collected using a ThermoFisher Talos Arctica transmission electron microscope equipped with a K3 Summit direct electron detector (Gatan) and operating at 200 kV and a magnification of 28,000 (corresponding to a pixel size of 1.4 Å). Defocus values for the images ranged from 1.5 to 2.5 μm. Further details of data collection/processing are listed in Supplementary Table 3.

Each DED frame stack was motion corrected using MotionCor2^51^ and CTF parameters were determined using Gctf^52^ in Relion 3.1.2. Well-preserved helical CA tubes were picked manually in Relion to obtain a total of 236,974 tube segments that were extracted into 600 × 600 pixel boxes with an overlap of ~90% between consecutive segments (13 asymmetric units). Extracted tube segments were subjected to many iterative rounds of 2D reference-free classification and classes with specific symmetries were selected for further processing. Two-fold binned images (2.8 Å/px) of tube segments in the selected 2D class were re-extracted into 300 px box size and analyzed with Relion 3D auto-refine, without enforcing helical symmetry and using a featureless cylindrical shell as initial reference. Helical “bubble” models with various helical parameters were generated using the “simulate_helix” command in Relion’s relion_helix_tool_box, and compared (using Chimera (UCSF) to the CA tube 3D volume obtained without application of helical symmetry. This resulted in estimation of preliminary helical parameters that were further refined in Relion to obtain final optimal values.

Refinement of the primary CA tube + GST-CPSF6_261-358_ map (shown in Fig. 5) involved calculation of a preliminary helical map (twist = −55.2731°, rise = 7.11505 Å) from 2,279 tube segments. A total of 530 micrographs from which these segments were extracted were identified and 17,618 segments were extracted from tubes contributing segments in each micrograph. This larger set of selected segments were 3D auto-refined again to obtain a map with a resolution of 9.6 Å. A single round of 3D classification (without realignment) was performed resulting in 3 classes. Each class was individually auto-refined and the best class, including 5,894 segments, was selected for further refinement. Final helical parameters were determined (twist = −55.2834°, rise = 7.12159 Å) and used to calculate a final map with a resolution of 7.9 Å (estimated using a Fourier Shell Correlation (FSC) cutoff of 0.143), which was post-processed, filtered by local resolution and had helical symmetry imposed.

Refinement of a second CA tube + GST-CPSF6_261-358_ map with a different helical symmetry (shown in Supplementary Fig. 11a) involved calculation of a preliminary helical map (twist = 138.178°, rise = 7.0475 Å) from 1400 tube segments. A total of 569 micrographs from which these segments were extracted were identified and 18,404 segments were extracted from tubes contributing segments in each micrograph. This large set of selected segments were 3D auto-refined again to obtain a map with a resolution of 8.4 Å. A single round of 3D classification (without realignment) was performed resulting in 3 classes. Each class was individually auto-refined and the best class, including 6212 segments, was selected for further refinement. Final helical parameters were determined (twist = 138.157°, rise = 7.06355 Å) and used to calculate a final map with a resolution of 7.4 Å (estimated by FSC cutoff of 0.143), which was post-processed, filtered by local resolution and had helical symmetry imposed.

For analysis of HIV-1 CA + GST-CPSF6_261-358_(ΔFG), cryo-EM samples were prepared as described above. The total number of initial segments extracted was 109,696 and initial helical parameters for images in the single best 2D class (1,426 tube segments) were determined (twist = 82.6362°, rise = 6.96812 Å). Extraction of all segments (17,462) from relevant tubes in all micrographs (491) contributing to the best initial 2D class and subsequent 3D refinement resulted in a map with a resolution of 8.4 Å. As described above, a single round of 3D classification was used to identify 3,023 tube segments contributing to the best 3D class, which led to a map with a resolution of 7.0 Å (twist = 82.5822°, rise = 6.95455 Å), which was post-processed, filtered by local resolution and had helical symmetry imposed. Further details of data collection and image processing are included in Supplementary Table 3.

### X-ray crystallography

CA_hex_ was mixed with 4 mM CPSF6_313–327_ peptide and then 1 mM IP6 was added in equal volume to the protein: peptide mixture to create the tripartite complex. The co-crystals were grown by hanging drop vapor diffusion at 18 °C with an equal volume of crystallization buffer. The crystallization buffer contained 8% PEG8000, 0.1 M Tris, pH 8.2, and 5% glycerol. The crystals appeared within one week. The cryogenic solution for the crystals consisted of crystallization buffer with additional PEG8000 and glycerol to reach 20 and 10%, respectively. Crystals were flash cooled in liquid nitrogen. The data was collected at the Advanced Light Source, Beamline 4.2.2 (Macromolecular Crystallography; MBC) at 100 K and a wavelength of 1.00003 Å. The data was processed and scaled with XDS^53^. PHASER^54^ in the PHENIX suite^55^ was used for molecular replacement using PDB 4U0B as a search model. The structure was refined using repetitive cycles of model building and refinement by COOT^56^ and phenix.refine^55^, respectively. TLS Motion Determination (TLSMD) was used to analyze the flexibility of the structures, which provided TLS parameters in phenix.refine that help in refining the structures for anisotropic displacements^55^. The IP6 molecules were independently positioned into the structure based on the Fo - Fc omit map density at 3 σ and refined with phenix.refine subsequently to confirm they fit the 2Fo - Fc density at 1 σ^55^. Find Water COOT program was originally used to add water molecules, but each water was individually assessed to ensure they fit the 2Fo - Fc density at 1σ^57^. Molprobity was utilized to evaluate the final model of the structure and ensure its quality^58^. The coordinates are deposited in the Protein Data Bank under accession code: 7SNQ [10.2210/pdb7SNQ/pdb]. The data collection and refinement statistics are given in Supplementary Table 4.

### AA MD simulation

The initial model of GST-CPSF6_261-358_ bound to the hexameric CA lattice was generated in Chimera. The available X-ray structure of the GST dimer (PDB:1GTA)^59^ was positioned in the respective cryo-EM density. The CPSF6 FG peptide bound to CA hexamer was generated based on our X-ray crystal structure of the CA_hex_ + IP6 + CPSF6_313–327_complex (Supplementary Fig. 12). The N-terminal LCR for each CPSF6 chain was then engineered within the density observed in the cryo-EM maps. The structure of the linker (SDIIPTTENLYFQGAIA) to connect the GST dimer to the N-terminal LCR was generated using MODELLER interfaced in chimera^60^. The C-terminal LCR fragment of the CPSF6 (residues 328-358) was engineered in RoseTTAfold^61^. The composite atomic system consisting of four CA hexamers and three GST-CPSF6_261-358_ has 54144 atoms. The system was then solvated with TIP3P water and neutralized by adding Na^+^ and Cl^−^ ions to the bulk solution. The final ionic composition of the system mimics physiologically relevant 150 mM NaCl. The final solvated and ionized system contains 1596719 atoms. Periodic boundary conditions were imposed on an orthorhombic unit cell of dimensions 24.5 nm × 28.6 nm × 22.4 nm. The composite CA hexamer-CPSF6 system was then minimized and equilibrated by applying harmonic positional restraints (spring constant value of 239 kcal/mol/nm^2^) on protein-heavy atoms for 500 ps. The system was then further equilibrated for 300 ps using the identical settings, and configurations were saved every 100 ps to be used for the three independent production runs. The restrained simulations were performed under constant volume and temperature (NVT) conditions and 310 K with a 2 fs time step. In the restrained simulations, temperature of the system was maintained using a stochastic velocity rescaling thermostat with a time constant of 1 ps. The production run simulations were carried out for 1000 ns in constant pressure and temperature (NPT) ensemble at 310 K and 1 bar. The temperature was maintained using Nose-Hoover chain thermostat with a 2 ps time constant, and pressure with isotropic (Parrinello-Rahman barostat with a 10 ps time constant. In the production simulations, positional restraints were used on 12 out of 24 CA monomers. The CA monomer directly complexed to the FG peptide of CPSF6, and the two neighboring CA monomers were not positionally restrained. The protein was modeled with CHARMM36m model^62^, and solvated with TIP3P water^63^. To constrain the bonds between heavy and hydrogen atoms we used the LINCS algorithm^64^. Electrostatic interactions were computed using the particle mesh Ewald method^65^, and van der Waals force was truncated smoothly to zero between 1.0 and 1.2 nm. AA MD simulations were performed using Gromacs 2019 package^66^. To calculate the spatial density and conformational cluster of the CPSF6 chains we first aggregated the last 800 ns of the simulation trajectory from all three production run replicates. The coordinates of the C_a_ atoms of the CPSF6_261-358_ were then used to calculate the time-averaged density in the VMD volmap plugin with a grid spacing of 2 Å×2 Å×2 Å^67^. The conformational clusters of the tri-CPSF6 complex were calculated in the gromacs “cluster” postprocessing analysis tool with RMSD cutoff of 5 Å^68^. The ﻿ensemble-averaged intrachain and interchain contact maps were calculated from the coordinates of the C_a_ atoms. Two residues were considered to be in contact in a trajectory frame if the distance between the C_a_ atoms were less than 10 Å. AA MD simulations were performed in the Beagle-3 GPU nodes provided by the University of Chicago Research Computing Center (RCC).

### AlphaFold

To predict the folding of CPSF6_261-358_/WT and corresponding CPSF6/FU, CPSF6/CD, CPSF6/AD and CPSF6/NE constructs the primary structures of these proteins were submitted to AlphaFold2^69^. The top results were visualized with ChimeraX^70^. The FG peptides from these proteins were aligned to the crystallographic structure of CA_hex_ bound to IP6 and CPSF6_313-327_ (Supplementary Fig. 12), using MatchMaker, with Needleman_Wunsch algorithm.

### HDX-MS

Solution-phase amide HDX experiments were carried out with a fully automated system (CTC HTS PAL, LEAP Technologies, Carrboro, NC; housed inside a 4 °C cabinet) as described^71^ with slight modifications. Peptides were identified using tandem MS (MS/MS) experiments performed on a QExactive (Thermo Fisher Scientific, San Jose, CA) over a 70 min gradient. Product ion spectra were acquired in a data-dependent mode and the five most abundant ions were selected for the product ion analysis per scan event. The MS/MS *.raw data files were converted to *.mgf files and then submitted to MASCOT (version 2.3 Matrix Science, London, UK) for peptide identification. The maximum number of missed cleavages was set at 4 with the mass tolerance for precursor ions ± 0.6 Da and for fragment ions ± 8 ppm. Pepsin was used for digestion and no specific enzyme was selected in MASCOT during the search. Peptides included in the peptide set used for HDX detection had a MASCOT score of 20 or greater. The MS/MS MASCOT search was also performed against a decoy (reverse) sequence and false positives were ruled out if they did not pass a 1% false discovery rate.

CA(A92E) tubes were initially formed overnight at 25 °C and then incubated with GST-CPSF6_261-358_ or GST-CPSF6_261-358_(ΔFG) for 2 min at 25 °C. For the differential HDX experiments, we compared; 1) GST-CPSF6_261-358_ ± CA(A92E) at a final CPSF6 to CA ratio of 1:12; 2) GST-CPSF6_261-358,ΔFG_ ± CA(A92E) at a final CPSF6 to CA ratio of 1:12; 3) CA(A92E) ± GST-CPSF6_261-358_ at a final CPSF6 to CA ratio of 0.8:1; 4) CA(A92E) ± GST-CPSF6_261-358_(ΔFG) at a final CPSF6 to CA ratio of 0.8:1. The reactions (5 μl) were mixed with 20 μl of D_2_O-containing HDX buffer (100 mM MOPS pD 6.9, 1 M NaCl, 200 μM IP6) and incubated at 4 °C for indicated times (Supplementary Table 5). Following on-exchange, unwanted forward- or back-exchange was minimized, and the protein was denatured by the addition of 25 μl of a quench solution (0.1 M Na Phosphate, 50 mM TCEP, pH 2).

Samples for Experiments 1 and 2 were then passed through an immobilized pepsin column (prepared in house) at 50 μl min^−1^ (0.1% v/v TFA, 4 °C) and the resulting peptides were trapped and desalted on a 2 mm × 10 mm C_8_ trap column (Hypersil Gold, ThermoFisher). The bound peptides were then gradient-eluted (4-40% CH3CN v/v and 0.3% v/v formic acid) across a 2.1 mm × 50 mm C_18_ separation column (Hypersil Gold, ThermoFisher) for 5 min. Sample handling and peptide separation were conducted at 4 °C. The eluted peptides were then subjected to electrospray ionization directly coupled to an Orbitrap mass spectrometer (QExactive, ThermoFisher).

Samples for Experiments 3 and 4 were then immediately passed through an immobilized pepsin column (prepared in house) at 150 μl min^−1^ (0.1% v/v TFA, 4 °C) and the resulting peptides were trapped and desalted on a 2 mm × 1 cm C_8_ trap column (Hypersil Gold, ThermoFisher). The bound peptides were then gradient-eluted (4-40% CH3CN v/v and 0.3% v/v formic acid) across a 2.1 mm × 5 cm C_18_ separation column (Hypersil Gold, ThermoFisher) for 5 min. Sample handling and peptide separation were conducted at 4 °C. The eluted peptides were then subjected to electrospray ionization directly coupled to an Orbitrap mass spectrometer (Exactive, ThermoFisher).

Each differential HDX experiment was performed with two biological replicate each with three technical replicates. The intensity weighted mean m/z centroid value of each peptide envelope was calculated and subsequently converted into a percentage of deuterium incorporation. This is accomplished by determining the observed averages of the undeuterated and fully deuterated spectra using the conventional formula described elsewhere^72^. The fully deuterated control, 100% deuterium incorporation, was calculated theoretically, and corrections for back-exchange were made on the basis of an estimated 70% deuterium recovery and accounting for 80% final deuterium concentration in the sample (1:5 dilution in D_2_O HDX buffer). Statistical significance for the differential HDX data is determined by an unpaired *t*-test for each time point, a procedure that is integrated into the HDX Workbench software^73^.

The HDX data from all overlapping peptides were consolidated to individual amino acid values using a residue averaging approach. Briefly, for each residue, the deuterium incorporation values and peptide lengths from all overlapping peptides were assembled. A weighting function was applied in which shorter peptides were weighted more heavily and longer peptides were weighted less. Each of the weighted deuterium incorporation values were then averaged incorporating this weighting function to produce a single value for each amino acid. The initial two residues of each peptide, as well as prolines, were omitted from the calculations. This approach is similar to that previously described^74^.

Deuterium uptake for each peptide was calculated as the average of %D for all on-exchange time points and the difference in average %D values between the unbound and bound samples was presented as a heat map with a color code given at the bottom of the figure (warm colors for deprotection and cool colors for protection). Peptides were colored by the software automatically to display significant differences, determined either by a >5% difference (less or more protection) in average deuterium uptake between the two states, or by using the results of unpaired *t*-tests at each time point (*p*-value < 0.05 for any two time points or a *p*-value < 0.01 for any single time point). Peptides with non-significant changes between the two states were colored grey. The exchange at the first two residues for any given peptide was not colored. Each peptide bar in the heat map view displays the average Δ %D values, associated standard deviation, and the charge state. Additionally, overlapping peptides with a similar protection trend covering the same region were used to rule out data ambiguity. The data have been deposited to the ProteomeXchange Consortium via the PRIDE^75^ partner repository with the data set identifier PXD030332.

### Statistical analysis

Statistical analysis of samples was carried out with Student’s two-sample, two-tailed *t*-test except sequencing of HIV-1 integration sites which is performed by Fisher’s exact text or Wilcoxon-Mann-Whitney rank sum test (for gene density).

### Reporting summary

Further information on research design is available in the Nature Research Reporting Summary linked to this article.

## Supplementary information

Supplementary Information

Reporting Summary

## Data Availability

All processed data is available in the manuscript or the supplementary materials. All raw data is available in the file of source data. Raw integration site sequencing results are deposited at the National Center for Biotechnology Sequences Read Archive with accession number PRJNA787708. The coordinates of the crystal structure of CA_hex_ + IP_6_ + CPSF6_313-327_ are deposited in the Protein Data Bank under accession code: 7SNQ. Cryo-EM maps of CA + IP6 + GST-CPSF6_261-358_ and CA + IP6 + GST-CPSF6_261-358_(ΔFG) are deposited to the Electron Microscopy Data Bank with EMDB codes EMD-27617, EMD-27619 and EMD-27625. HDX MS results are deposited to the ProteomeXchange Consortium via the PRIDE partner repository with the data set identifier PXD030332. Source data are provided with this paper.

## Source data

Peer Review
Source Data
