# Supplementary material for: Prion-like low complexity regions enable avid virus-host interactions during HIV-1 infection
Source: Nat Commun. 2022 Oct 6;13:5879. doi: 10.1038/s41467-022-33662-6 (PMC9537594; doi:10.1038/s41467-022-33662-6)
Supplement: Supplementary file 1 — Supplementary Information [file 41467_2022_33662_MOESM1_ESM.pdf]

Prion-like low complexity regions enable avid virus-host interactions during HIV-1 infection

Guochao Wei, Naseer Iqbal, Valentine V. Courouble, Ashwanth C. Francis, Parmit K. Singh, Arpa Hudait, Arun S. Annamalai, Stephanie Bester, Szu-Wei Huang, Nikoloz Shkriabai, Lorenzo Briganti, Reed Haney, Vineet N. KewalRamani, Gregory A. Voth, Alan N. Engelman, Gregory B. Melikyan, Patrick R. Griffin, Francisco Asturias, Mamuka Kvaratskhelia

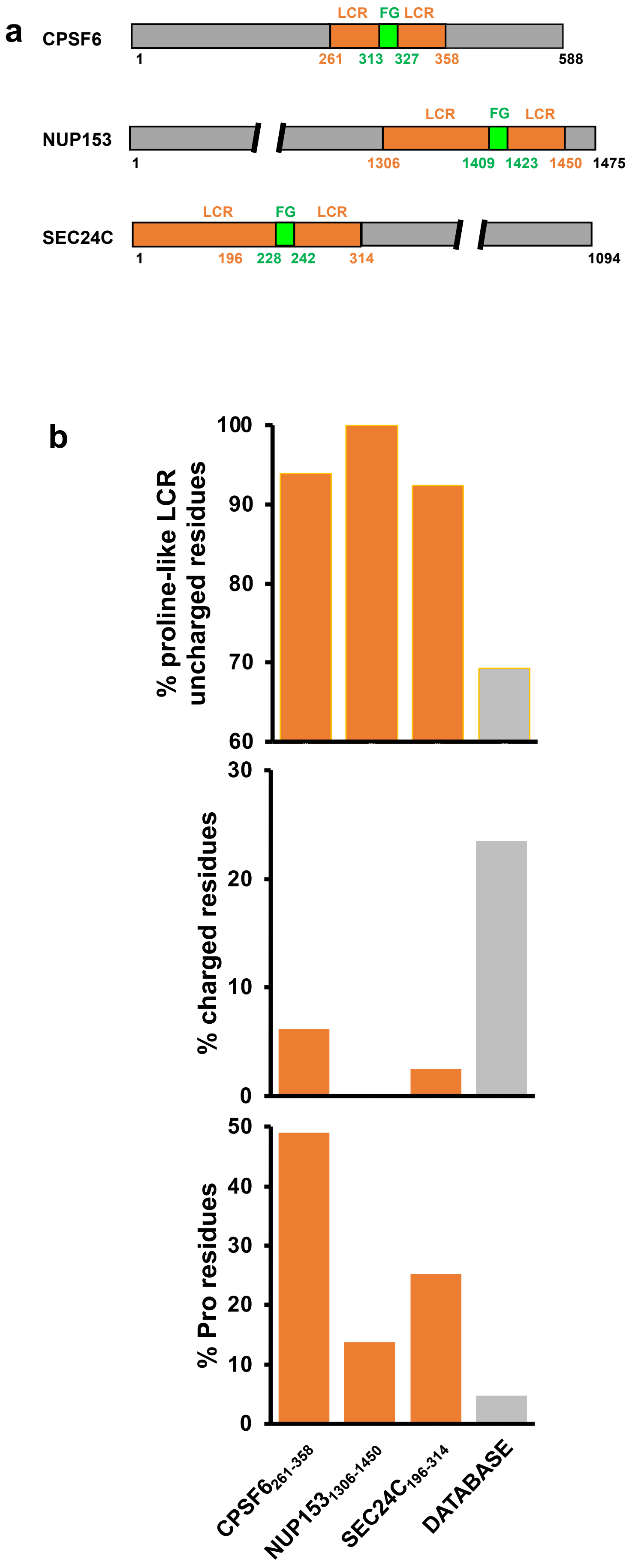
a
CPSF6
LCR FG LCR
1 261 313 327 358 588
NUP153
LCR FG LCR
1 1306 1409 1423 1450 1475
SEC24C
LCR FG LCR
1 196 228 242 314 1094
b
% proline-like LCR uncharged residues
100
90
80
70
60
% charged residues
30
20
10
0
% Pro residues
50
40
30
20
10
0
CPSF6261-358
NUP1531306-1450
SEC24C196-314
DATABASE

Supplementary Figure 1. Prion-like LCRs in CPSF6, NUP153 and SEC24C. (**a**) Schematic of CPSF6, NUP153 and SEC24C to show the prion-like LCR (orange) and FG containing peptides (green). (**b**) Relative abundance of prion-like LCR defining uncharged (top), charged (middle) and Pro (bottom) residues in indicated protein segments. The values were calculated by dividing numbers of prion-like LCR defining uncharged (Ala, Gly, Val, Phe, Tyr, Leu, Ile, Ser, Thr, Pro, Asn, Gln), charged (Asp, Glu, Lys, Arg), and Pro residues by total number of amino acids in respective protein fragments. For the database values an average of all protein sequences deposited in the UniProtKB/Swiss-Prot database were utilized. The database consisted of 565928 sequence entries from 281485 unique references comprising 204173280 amino acids at the time of analysis (https://web.expasy.org/docs/relnotes/relstat.html). The database breaks down each amino acid into a percentage of the total number of amino acids. Source data are provided as a Source Data file. Abbreviation: LCR: low complexity region.

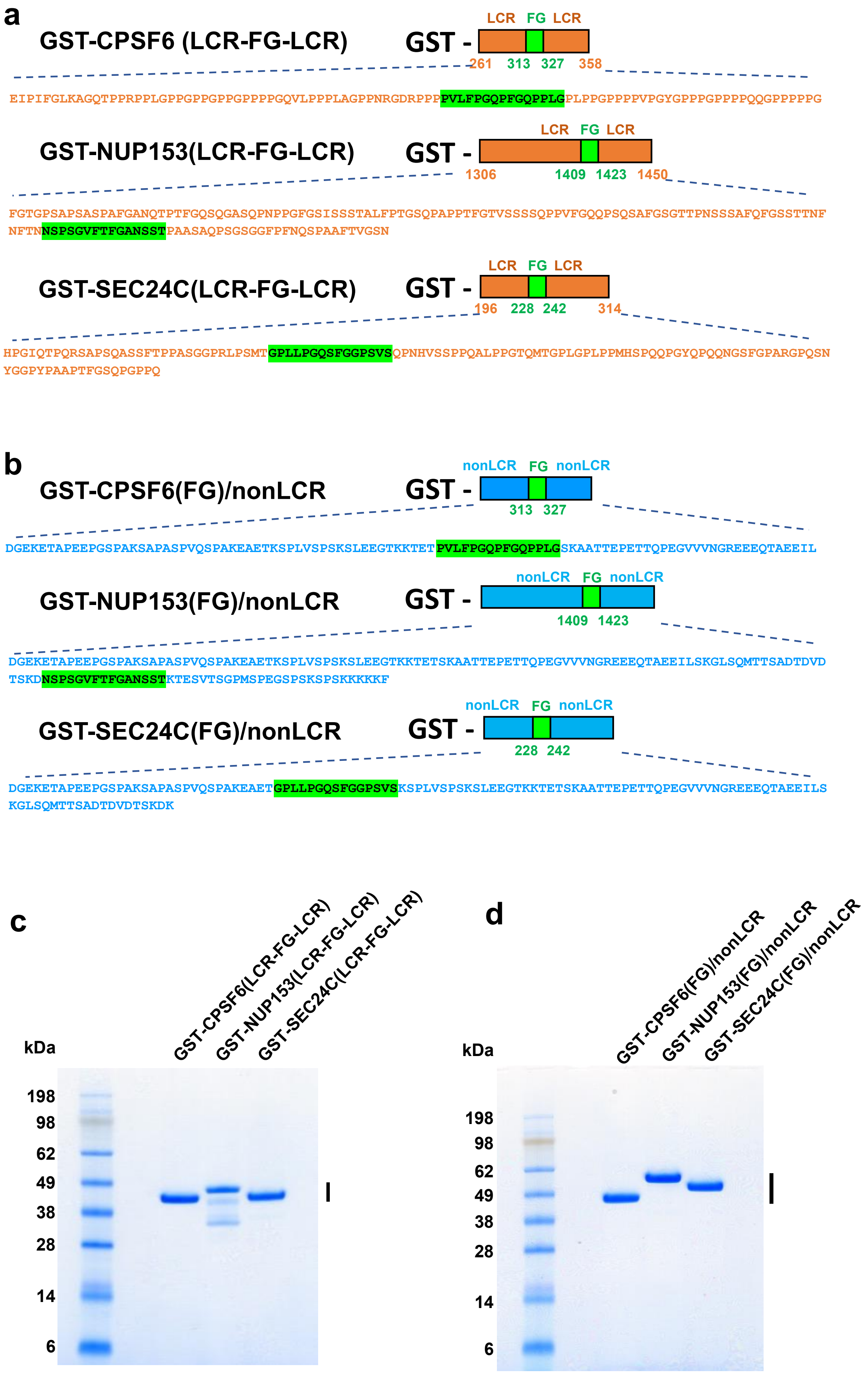

a
GST-CPSF6 (LCR-FG-LCR)
GST -
LCR FG LCR
261 313 327 358
EIPIFGLKAGQTPPRPPLGPPGPPGPPGPPPPGQVLPPPLAGPPNRGDRPPPPVLFPGQPFGQPPLGPLPPGPPPPVPGYGPPPGPPPPQQGPPPPPG
GST-NUP153(LCR-FG-LCR)
GST -
LCR FG LCR
1306 1409 1423 1450
FGTGPSAPSASPAFGANQTPTFGQSQGASQPNPPGFGSISSSTALFPTGSQPAPPTFGTVSSSSQPPVFGQQPSQSAFGSGTTPNSSSAFQFGSSTTNF
NFTNNSPSGVFTFGANSSTPAASAQPSGSGGFPFNQSPAAFTVGSN
GST-SEC24C(LCR-FG-LCR)
GST -
LCR FG LCR
196 228 242 314
HPGIQTPQRSAPSQASSFTPPASGGPRLPSMTGPLLPGQSFGGPSVSQPNHVSSPPQALPPGTQMTGPLGPLPPMHSPQQPGYQPQQNGSFGPARGPQSN
YGGPYPAAPTFGSQPGPPQ
b
GST-CPSF6(FG)/nonLCR
GST -
nonLCR FG nonLCR
313 327
DGEKETAPEEPGSPAKSAPASPVQSPAKEAETKSPLVSPSKSLEEGTKKTETPVLFPGQPFGQPPLGSKAATTEPETTQPEGVVVNGREEEQTAEEIL
GST-NUP153(FG)/nonLCR
GST -
nonLCR FG nonLCR
1409 1423
DGEKETAPEEPGSPAKSAPASPVQSPAKEAETKSPLVSPSKSLEEGTKKTETSKAATTEPETTQPEGVVVNGREEEQTAEEILSKGLSQMTTSADTDVD
TSKDNSPSGVFTFGANSSTKTESVTSGPMSPEGSPSKSPSKKKKKF
GST-SEC24C(FG)/nonLCR
GST -
nonLCR FG nonLCR
228 242
DGEKETAPEEPGSPAKSAPASPVQSPAKEAETGPLLPGQSFGGPSVSKSPLVSPSKSLEEGTKKTETSKAATTEPETTQPEGVVVNGREEEQTAEEILS
KGLSQMTTSADTDVDTSKDK
c
kDa
198
98
62
49
38
28
14
6
GST-CPSF6(LCR-FG-LCR)
GST-NUP153(LCR-FG-LCR)
GST-SEC24C(LCR-FG-LCR)
d
kDa
198
98
62
49
38
28
14
6
GST-CPSF6(FG)/nonLCR
GST-NUP153(FG)/nonLCR
GST-SEC24C(FG)/nonLCR

Supplementary Figure 2. Preparation of recombinant proteins. Schematic of WT (**a**) and chimeric (**b**) CPSF6, NUP153 and SEC24C proteins. Representative SDS-PAGE images of WT (**c**) and chimeric (**d**) purified recombinant proteins are shown. Expression of target proteins are indicated by a side bar. The experiment was repeated 3 times independently with similar results. Abbreviation: LCR: low complexity region.

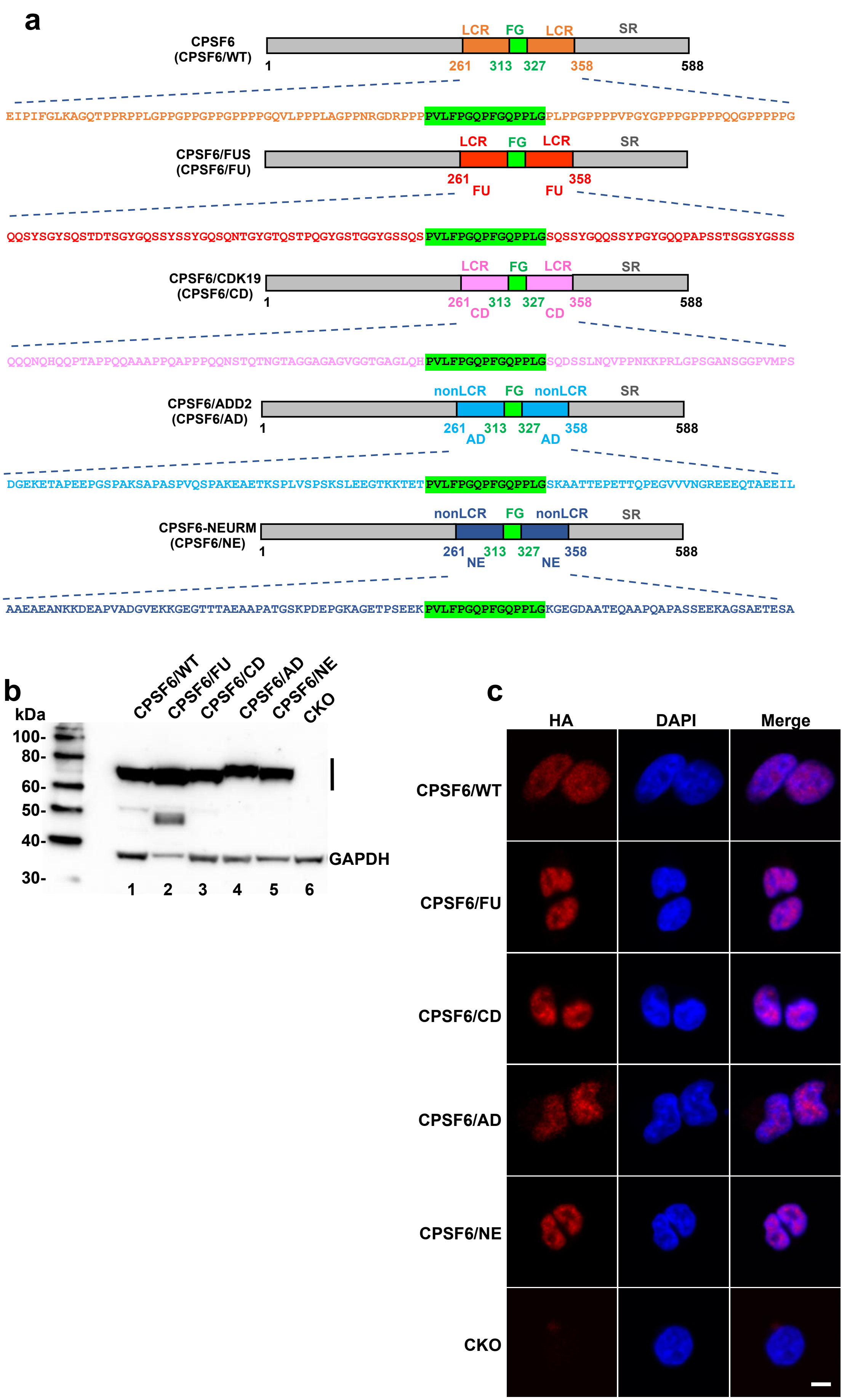
a
LCR
FG
LCR
SR
CPSF6
(CPSF6/WT)
1
261
313
327
358
588
EIPIFGLKAGQTPPRPPLGPPGPPGPPGPPPPGQVLPPPLAGPPNRGDRPPPPVLFPGQPFGQPPLGPLPPGPPPPVPGYGPPPGPPPPQQGPPPPPG
CPSF6/FUS
(CPSF6/FU)
261
FU
358
FU
QQSYSGYSQSTDTSGYGQSSYSSYGQSQNTGYGTQSTPQGYGSTGGYGSSQSPVLFPGQPFGQPPLGSQSSYGQQSSYPGYGQQPAPSSTSGSYGSSS
CPSF6/CDK19
(CPSF6/CD)
CD
CD
QQQNQHQQPTAPPQQAAAPPQAPPPQQNSTQTNGTAGGAGAGVGGTGAGLQHPVLFPGQPFGQPPLGSQDSSLNQVPPNKKPRLGPSGANSGGPVMPS
nonLCR
nonLCR
CPSF6/ADD2
(CPSF6/AD)
AD
AD
DGEKETAPEEPGSPAKSAPASPVQSPAKEAETKSPLVSPSKSLEEGTKKTETPVLFPGQPFGQPPLGSKAATTEPETTQPEGVVVNGREEEQTAEEIL
CPSF6-NEURM
(CPSF6/NE)
NE
NE
AAEAEANKKDEAPVADGVEKKGEGTTTAEAAPATGSKPDEPGKAGETPSEEKPVLFPGQPFGQPPLGKGEGDAATEQAAPQAPASSEEKAGSAETESA
b
CPSF6/WT
CPSF6/FU
CPSF6/CD
CPSF6/AD
CPSF6/NE
CKO
kDa
100-
80-
60-
50-
40-
30-
GAPDH
1
2
3
4
5
6
c
HA
DAPI
Merge
CPSF6/WT
CPSF6/FU
CPSF6/CD
CPSF6/AD
CPSF6/NE
CKO

Supplementary Figure 3. Overexpression of WT and chimeric full-length CPSF6 proteins in CKO cells. (**a**) Schematic of WT and chimeric CPSF6 proteins. CPSF6/FU: CPSF6 LCR segments flanking $CPSF6_{313-327}$(FG) (green) were replaced with alternative LCR segments from FUS_{35-117} (red). CPSF6/CD: CPSF6 LCR segments flanking $CPSF6_{313-327}$(FG) (green) were replaced with alternative LCR segments from $CDK19_{374-456}$ (magenta). CPSF6/AD: CPSF6 LCR segments flanking $CPSF6_{313-327}$(FG) (green) were replaced with non-LCR, flexible segments from $ADD2_{580-662}$ (cyan). CPSF6/NE: CPSF6 LCR segments flanking $CPSF6_{313-327}$(FG) (green) were replaced with non-LCR, flexible segments from $NEURM_{97-179}$ (dark blue). (**b**) Representative immunoblotting to show expression of WT and chimeric CPSF6 proteins in CKO cells. Briefly, CPSF6-KO 293T cells were transduced with VSV-G pseudotyped MLV-based retroviral vector encoding HA-tagged WT and chimeric CPSF6 proteins. Transduced cells were selected with 2μg/ml puromycin. Expression of target proteins are indicated by a side bar. The experiment was repeated 3 times independently with similar results. (**c**) Immunofluorescence to detect the expression of WT and chimeric CPSF6 proteins in CKO cells. Cells were immunostained for HA-tagged protein (red) and nuclei DAPI (blue). Scale bar is 5 μm. The experiment was repeated 3 times independently with similar results. Abbreviations: AD: ADD2; NE: NEURM; FU: FUS; CD: CDK19; CKO: CPSF6 knock-out; LCR: low complexity region.

a

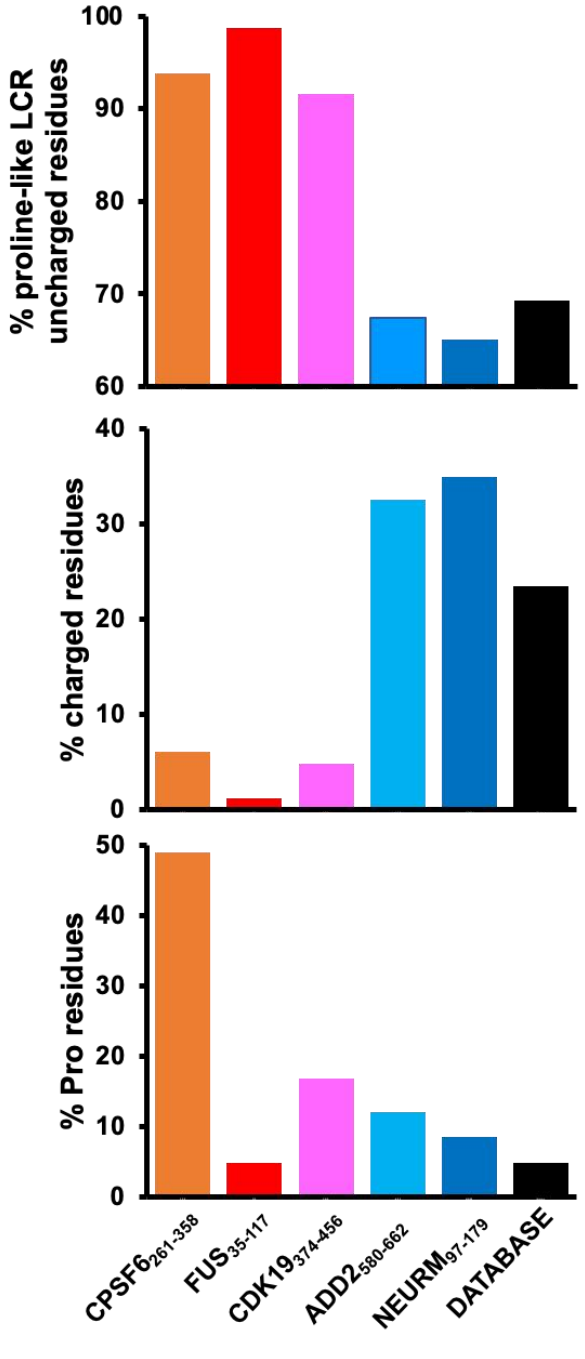

% proline-like LCR uncharged residues
100
90
80
70
60
% charged residues
40
30
20
10
0
% Pro residues
50
40
30
20
10
0
CPSF6261-358
FUS35-117
CDK19374-456
ADD2580-662
NEURM97-179
DATABASE

b

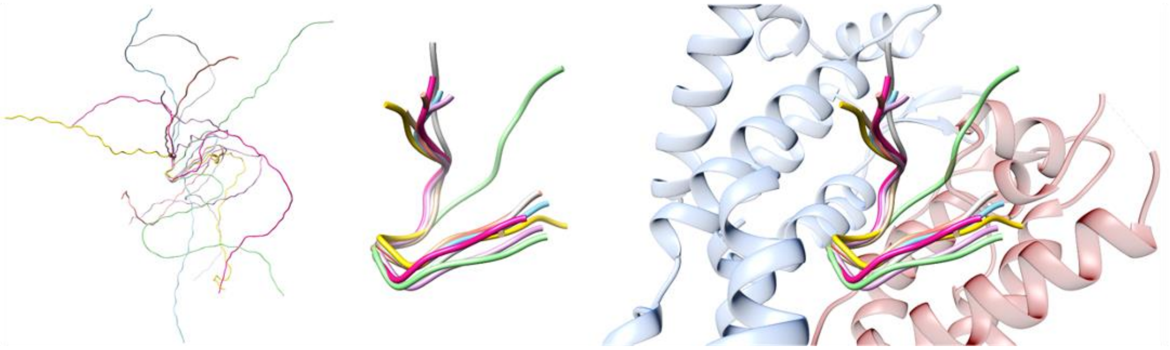

Supplementary Figure 4. Amino acid composition and AlphaFold structural predictions of chimeric protein segments. (**a**) Relative abundance of prion-like LCR defining uncharged (top), charged (middle) and Pro (bottom) residues in indicated protein segments. The values were derived as explained in the legend of Supplementary Figure 1. Source data are provided as a Source Data file (**b**) AlphaFold results from WT $CPSF6_{261-358}$ and corresponding chimeric protein segments. Left panel: WT and chimeric protein segments aligned through the conserved 15-mer CPSF6 FG peptide. Middle panel: a zoomed in view of the 15-mer CPSF6 peptide in the context of WT CPSF6 (yellow), CPSF6(FG)/FU (green), CPSF6(FG)/CD (purple), CPSF6(FG)/AD (light blue) and CPSF6(FG)/NE (pink). Right panel: The 15-mer CPSF6 peptides from chimeric proteins were aligned with crystallographic WT CPSF6 FG peptide bound to CA_{hex} (Supplementary Fig 12, for clarity only one binding site formed by two CA subunits colored in light cyan and light magenta is shown). Note, only noticeable difference seen for the FG peptides is in the context of CPSF6(FG)/FU (where native LCRs were replaced with alternative LCRs from FUS). The 15-mer FG peptide adopts a slightly more closed "U" shaped conformation in CPSF6(FG)/FU (green) compared with its WT counterpart (yellow). Importantly, LCR containing full-length CPSF6/FU and GST-CPSF6(FG)/FU proteins were fully functional in virology and biochemistry assays. No noticeable differences in the "U" shape conformation of the FG peptides or their predicted interactions with the hydrophobic CA pocket were observed in the context of nonLCR chimeric proteins CPSF6(FG)/AD (light blue) and CPSF6(FG)/NE (pink), or LCR containing CPSF6(FG)/CD (purple).

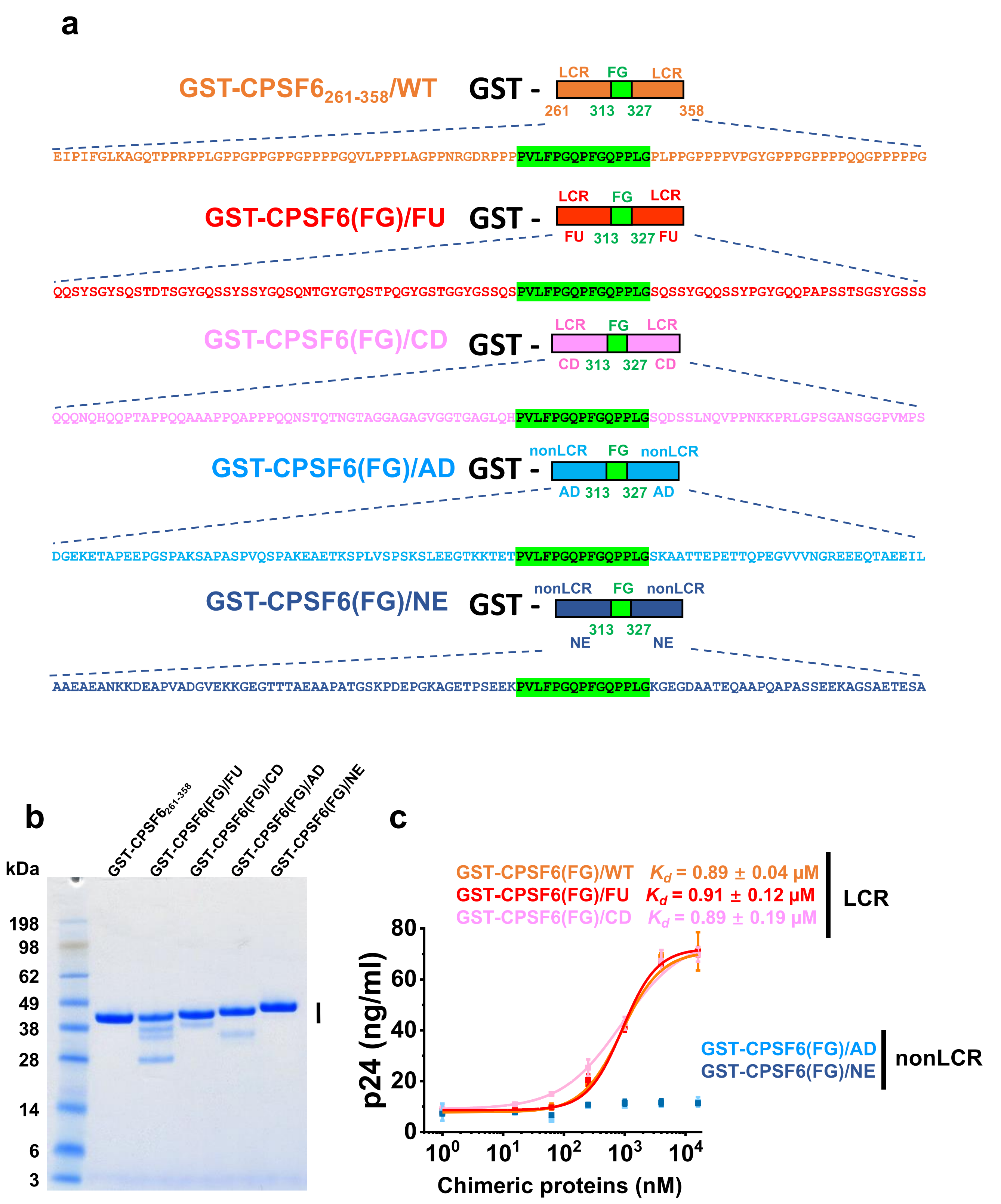

Supplementary Figure 5. Interactions of recombinant WT GST-CPSF6$_{261\text{-}358}$ and corresponding chimeric proteins with isolated native HIV-1 cores. (a) Schematic of WT and chimeric proteins. (b) Representative SDS-PAGE image of indicated purified recombinant proteins used for binding assays. (c) Quantification of GST-mediated affinity pull-down of native HIV-1 cores with indicated concentrations of purified recombinant proteins: GST-CPSF6261-358/WT; GST-CPSF6(FG)/FU; GST-CPSF6(FG)/CD; GST-CPSF6(FG)/AD; GST-

CPSF6(FG)/NE. The data (mean values +/− SD) from three independent experiments are shown. The results were analyzed by Origin 2019 (v.9.6) software to determine binding Kd values. Source data are provided as a Source Data file. Abbreviations: AD: ADD2; NE: NEURM; FU: FUS; CD: CDK19; LCR: low complexity region.

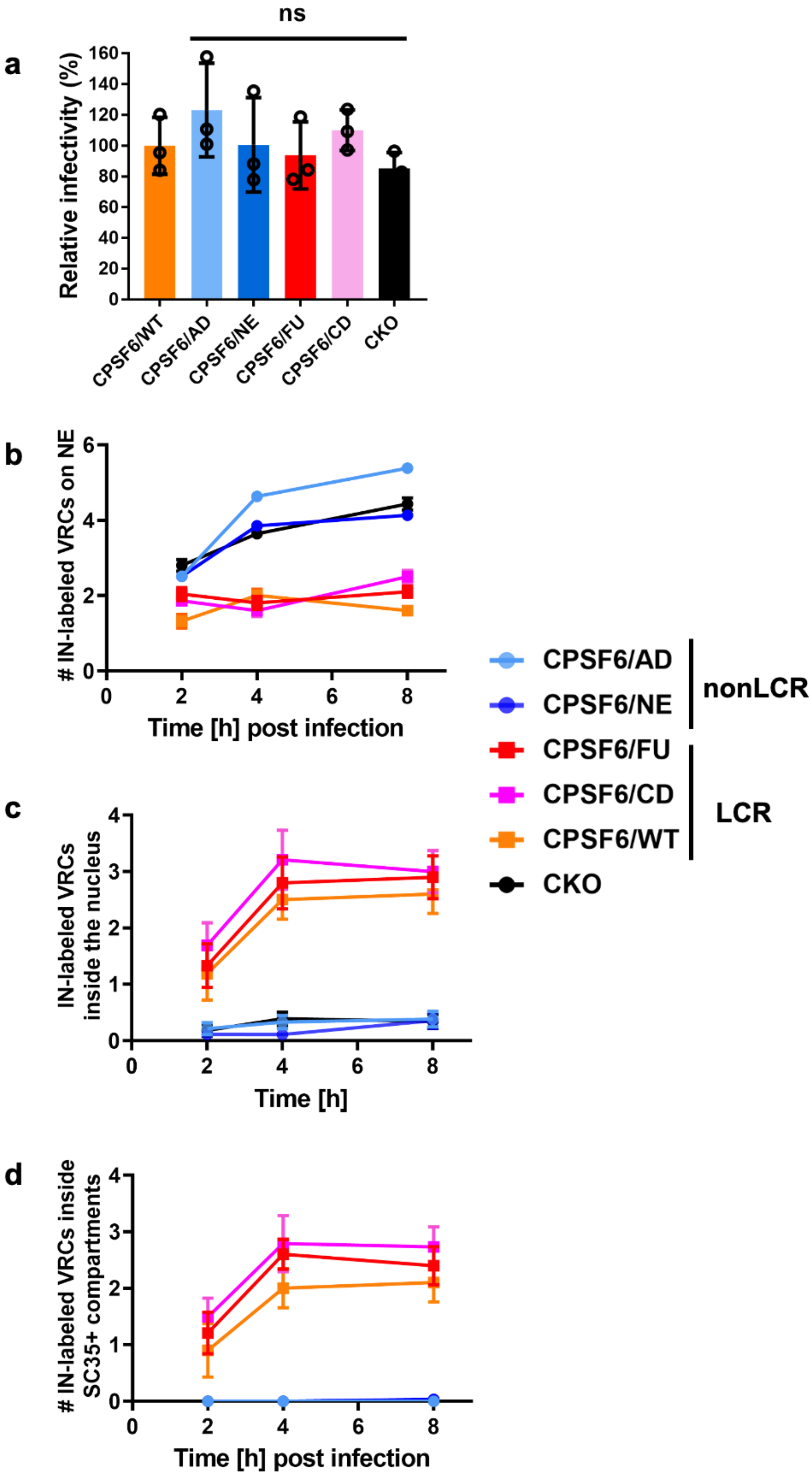
a
ns
Relative infectivity (%)
160
140
120
100
80
60
40
20
0
CPSF6/WT
CPSF6/AD
CPSF6/NE
CPSF6/FU
CPSF6/CD
CKO
b
IN-labeled VRCs on NE
6
4
2
0
0
2
4
6
8
Time [h] post infection
CPSF6/AD
CPSF6/NE
nonLCR
CPSF6/FU
CPSF6/CD
CPSF6/WT
LCR
CKO
c
IN-labeled VRCs inside the nucleus
4
3
2
1
0
0
2
4
6
8
Time [h]
d
IN-labeled VRCs inside SC35+ compartments
4
3
2
1
0
0
2
4
6
8
Time [h] post infection

Supplementary Figure 6. Infectivity and kinetics of nuclear import of HIV-1 VRCs in cells expressing the chimeric proteins. (a) Relative infectivity of VSV-G pseudotyped viruses in CKO cells stably expressing indicated CPSF6 chimeric proteins. The data (mean ± s.d.) from three independent experiments are shown. Statistical analysis was carried out with Student's two-sample, two-tailed t-test (b-d) CKO cells stably expressing the indicated CPSF6 chimeric proteins were infected with INsfGFP labeled HIV pseudovirus. Cells were fixed at indicated time points, immuno-stained for nuclear speckles (SC35+) and the number of IN-labeled viral replication complexes (VRCs) in the nucleus was determined by confocal imaging. (b) Quantification of IN-VRCs on nuclear envelop. (c) The fraction of nuclei with IN-VRCs inside 0.5 µm of the nucleoplasm. (d) Quantification of IN-VRCs colocalized with NSs. Data shows mean and SEM from >30 nuclei (n=31 at 2 h, n=36 at 4 h and n=32 at 8 h for each sample) analyzed in 2 independent experiment. Statistical significance of comparison for WT versus chimeric CPSF6 proteins was determined by Student t-test. $P > 0.05$ was considered not significant (ns). Source data are provided as a Source Data file. Abbreviations: AD: ADD2; NE: NEURM; FU: FUS; CD: CDK19; CKO: CPSF6 knock-out; LCR: low complexity region.

a

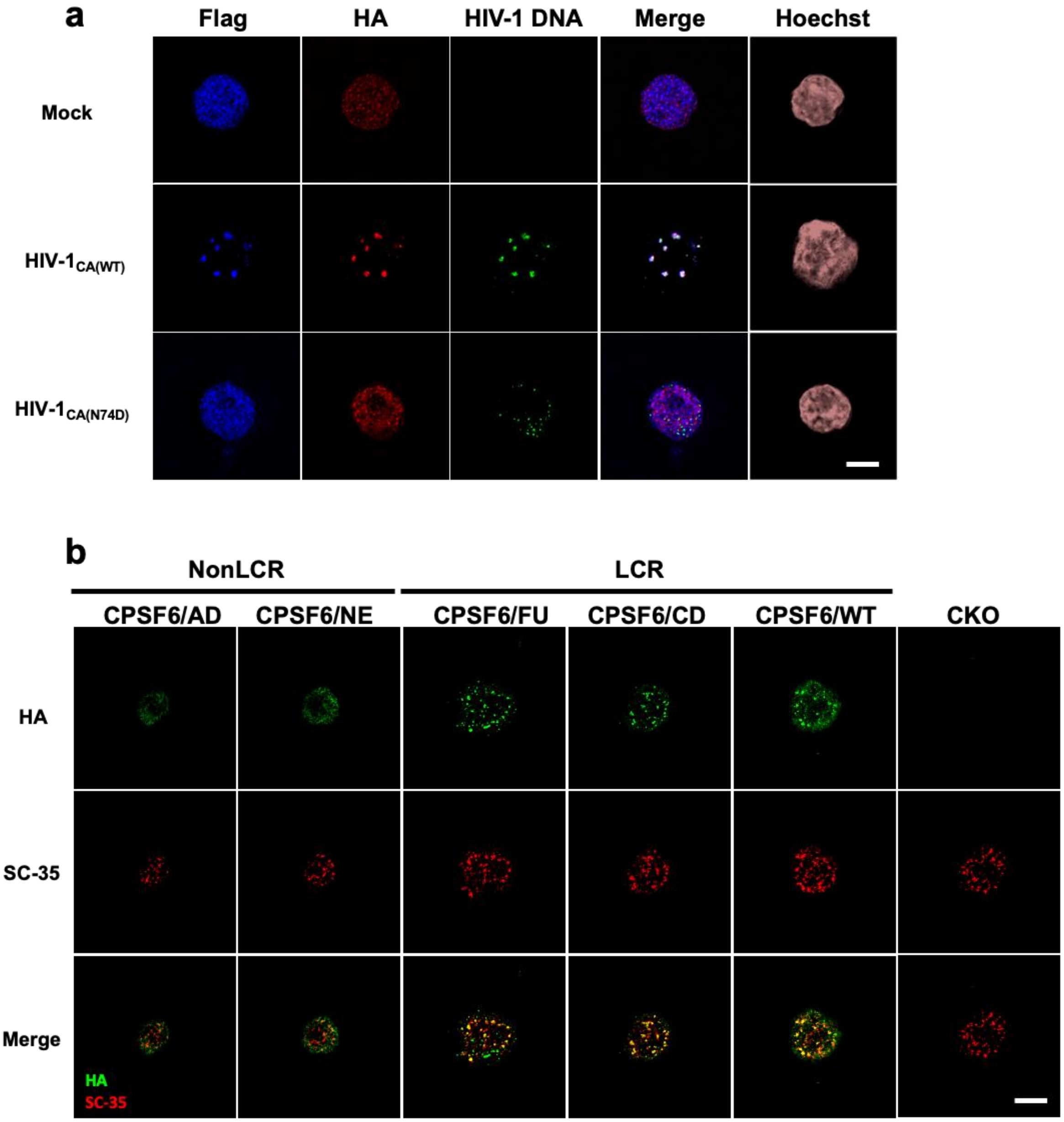

c

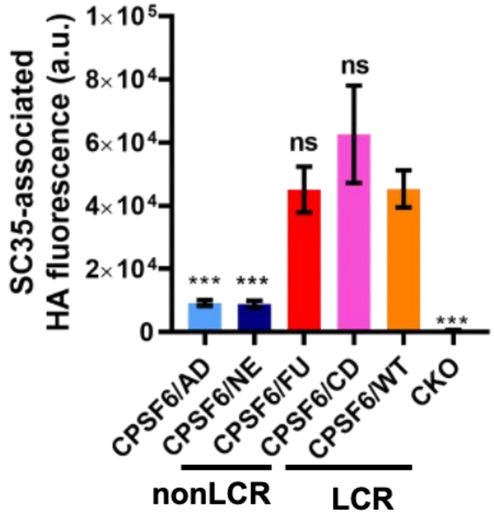

CPSF6/AD	1.43×10^{-25}
CPSF6/NE	1.87×10^{-24}
CPSF6/FU	0.98
CPSF6/CD	0.34
CKO	9.5×10^{-107}

Supplementary Figure 7. Prion-like LCR containing WT and chimeric CPSF6 proteins accumulate around HIV-1 cores in the nuclei of infected cells. (a) WT CPSF6 accumulates around HIV-1 cores in the nuclei of infected cells. Parental HEK293T and CKO cells expressing both HA- and flag-tagged full-length CPSF6 were infected with WT and N74D HIV-1 at MOI 50. After 6 hpi the samples were collected for immuno-fluorescence in situ hybridization (immuno-FISH). HIV-1 DNA was detected using a pool of probes targeting HIV-1 integrase region. Green indicates HIV-1 DNA. Red indicates HA-tagged CPSF6. Blue indicates Flag-tagged CPSF6. A single z section is presented. Scale bar is 5 µm. The experiment was repeated 3 times independently with similar results. (b) Immunofluorescence to detect the accumulation of CPSF6 and chimeric proteins in HIV-1 infected HEK293T CKO cells. CKO 293T cells overexpressing HA tagged WT and chimeric CPSF6 proteins were infected with WT HIV-1 (MOI 50). After 6 hpi, cells were fixed and immunostained with anti-HA antibody (ab236632, Abcam) and anti-SC-35 antibody (ab11826, abcam). Green indicates HA-tagged WT and chimeric CPSF6 proteins. Red indicates nuclear speckles marker SC-35. A single z section is presented. Scale bar is 5 µm. (c) Fluorescent intensity of HA tagged WT and chimeric CPSF6 proteins associated with SC35 in infected cells. Data was collected and analyzed from >6 cells (n=20 for CPSF6/WT, CPSF6/FU, CPSF6/AD and CPSF6/NE; n=8 for CPSF6/CD) each by using ICY image analysis software (http://icy.bioimageanalysis.org/) and shown with mean and SEM. Statistical significances of comparison of CPSF6/WT versus CPSF6/AD, CPSF6/NE, CPSF6/FU, CPSF6/CD and CKO were determined by Student's two-sample, two-tailed t-test. $P > 0.05$ was considered not significant (ns) and $p < 0.0001$ was considered highly significant (***). P values of comparison for WT versus chimeric CPSF6 proteins were shown in the table on the right. Source data are provided as a Source Data file. Abbreviations: AD: ADD2; NE: NEURM; FU: FUS; CD: CDK19; CKO: CPSF6 knock-out; LCR: low complexity region; a.u.: arbitrary units.

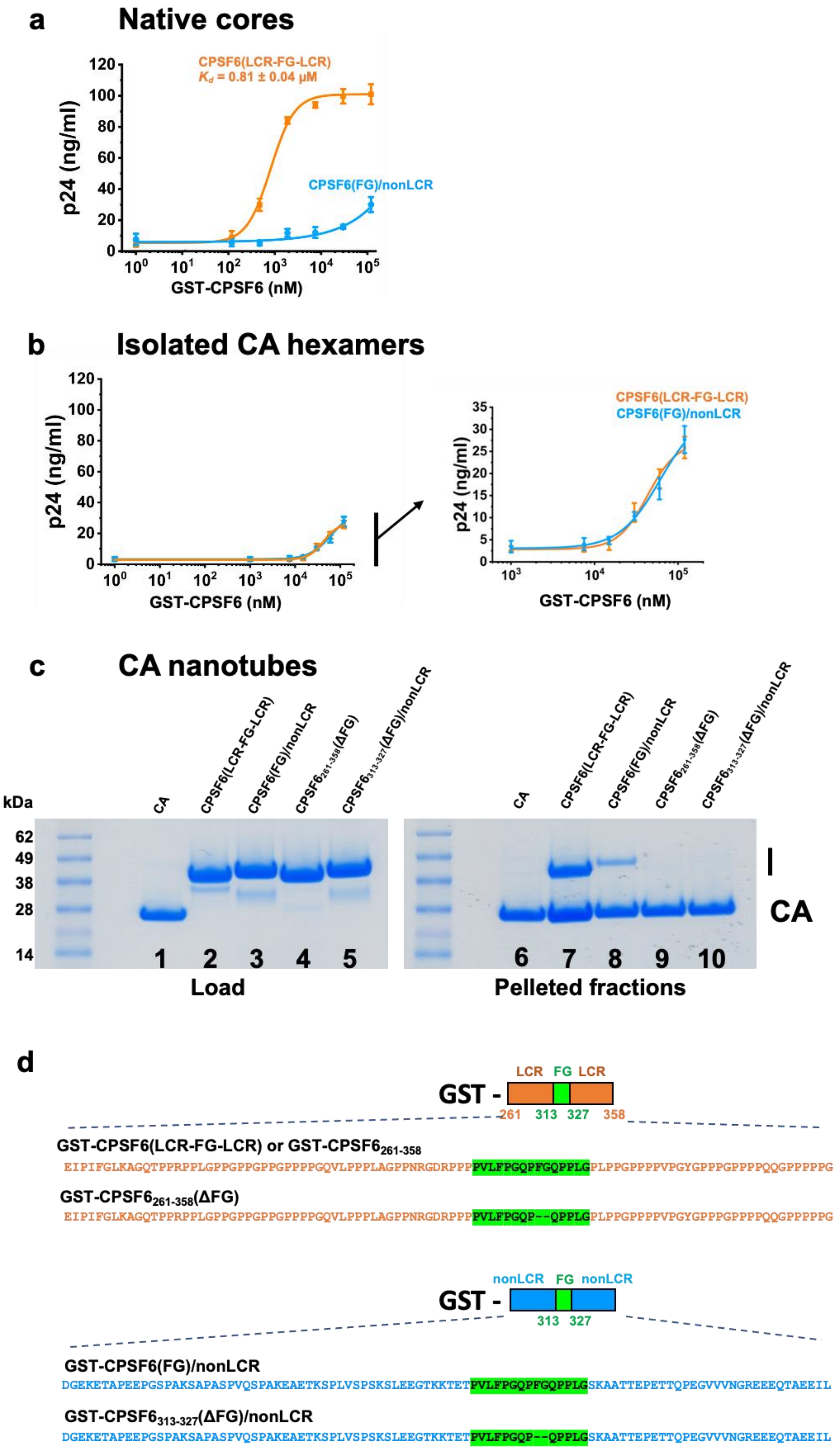
a
Native cores
CPSF6(LCR-FG-LCR)
K_d = 0.81 ± 0.04 μM
CPSF6(FG)/nonLCR
p24 (ng/ml)
GST-CPSF6 (nM)
b
Isolated CA hexamers
CPSF6(LCR-FG-LCR)
CPSF6(FG)/nonLCR
p24 (ng/ml)
GST-CPSF6 (nM)
c
CA nanotubes
kDa
CA
CPSF6(LCR-FG-LCR)
CPSF6(FG)/nonLCR
CPSF6261-358(ΔFG)
CPSF6313-327(ΔFG)/nonLCR
62
49
38
28
14
1 2 3 4 5
Load
6 7 8 9 10
Pelleted fractions
CA
d
LCR FG LCR
GST -
261 313 327 358
GST-CPSF6(LCR-FG-LCR) or GST-CPSF6261-358
EIPIFGLKAGQTPPRPPLGPPGPPGPPGPPPPGQVLPPPLAGPPNRGDRPPPPVLFPGQPFGQPPLGPLPPGPPPPVPGYGPPPGPPPPQQGPPPPPG
GST-CPSF6261-358(ΔFG)
EIPIFGLKAGQTPPRPPLGPPGPPGPPGPPPPGQVLPPPLAGPPNRGDRPPPPVLFPGQP--QPPLGPLPPGPPPPVPGYGPPPGPPPPQQGPPPPPG
nonLCR FG nonLCR
GST -
313 327
GST-CPSF6(FG)/nonLCR
DGEKETAPEEPGSPAKSAPASPVQSPAKEAETKSPLVSPSKSLEEGTKKTETPVLFPGQPFGQPPLGSKAATTEPETTQPEGVVVNGREEEQTAEEIL
GST-CPSF6313-327(ΔFG)/nonLCR
DGEKETAPEEPGSPAKSAPASPVQSPAKEAETKSPLVSPSKSLEEGTKKTETPVLFPGQP--QPPLGSKAATTEPETTQPEGVVVNGREEEQTAEEIL

Supplementary Figure 8. Interactions of recombinant GST-CPSF6(LCR-FG-LCR) and GST-CPSF6(FG)/nonLCR proteins with CA assemblies. (a) Quantitation of GST-mediated affinity pull-down of native HIV-1 cores bound to indicated concentrations of GST-CPSF6(LCR-FG-LCR) and GST-CPSF6(FG)/nonLCR proteins. Protein solubility capped the tested concentration range at 120 µM for GST-CPSF6(LCR-FG-LCR) and GST-CPSF6(FG)/nonLCR. GST-CPSF6(LCR-FG-LCR) bound to native cores with high affinity, whereas GST-CPSF6(FG)/nonLCR displayed low affinity binding. The averaged data (+/− SD) from three independent experiments are shown. Source data are provided as a Source Data file (**b**) Quantification of GST-mediated affinity pull-down of isolated, cross-linked CA hexamers bound to indicated concentrations of GST-CPSF6(LCR-FG-LCR) and GST-CPSF6(FG)/nonLCR; Both GST-CPSF6(LCR-FG-LCR) and GST-CPSF6(FG)/nonLCR displayed similarly low affinity binding to isolated CA hexamers. Collectively, results in (**a**) and (**b**) indicate the importance of both CPSF6 LCR and the mature CA lattice for high affinity binding. The averaged data (+/− SD) from three independent experiments are shown. Source data are provided as a Source Data file (**c**) Representative SDS-PAGE images of co-pelleting of indicated proteins with IP6 stabilized CA nanotubes. The protein concentrations and experimental conditions used in these assays closely mimic cryo-EM sample preparations. GST-CPSF6(LCR-FG-LCR) robustly bound to CA tubular assemblies, whereas consistent with a low affinity binding of the FG peptide in the absence of the flanking LCRs, only residual binding of GST-CPSF6(FG)/nonLCR to CA nanotubes was observed (compare lane 8 to lane 9). The analysis of corresponding GST-$CPSF6_{261-358}$(ΔFG) and GST-$CPSF6_{313-327}$(ΔFG)/nonLCR control proteins indicated that both high and low affinity interactions require the FG peptide. The experiment was repeated 3 times independently with similar results. (**d**) Schematics and primary sequences of GST-CPSF6(LCR-FG-LCR) (also referred here to as GST-$CPSF6_{261-358}$), GST-CPSF6(FG)/nonLCR, GST-$CPSF6_{261-358}$(ΔFG) and GST-$CPSF6_{313-327}$(ΔFG)/nonLCR constructs. Abbreviation: LCR: low complexity region.

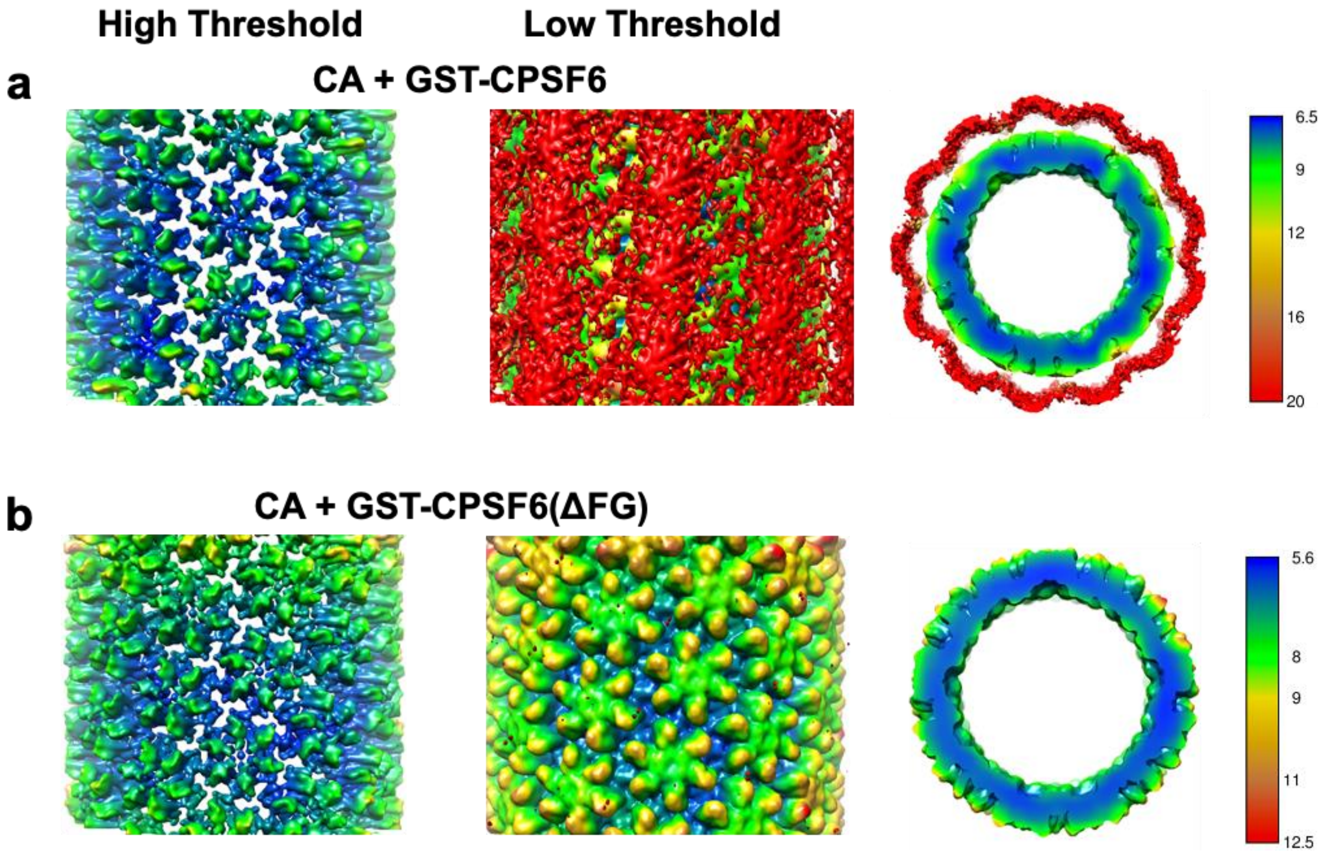

Supplementary Figure 9. Cryo-EM maps of IP6-stabilized CA nanotubes in the presence of GST-CPSF6$_{261-358}$ or GST-CPSF6$_{261-358}$(ΔFG). (**a**) The GST-CPSF6$_{261-358}$ cryo-EM map colored according to local resolution. (**b**) The GST-CPSF6$_{261-358}$(ΔFG) cryo-EM map colored according to local resolution.

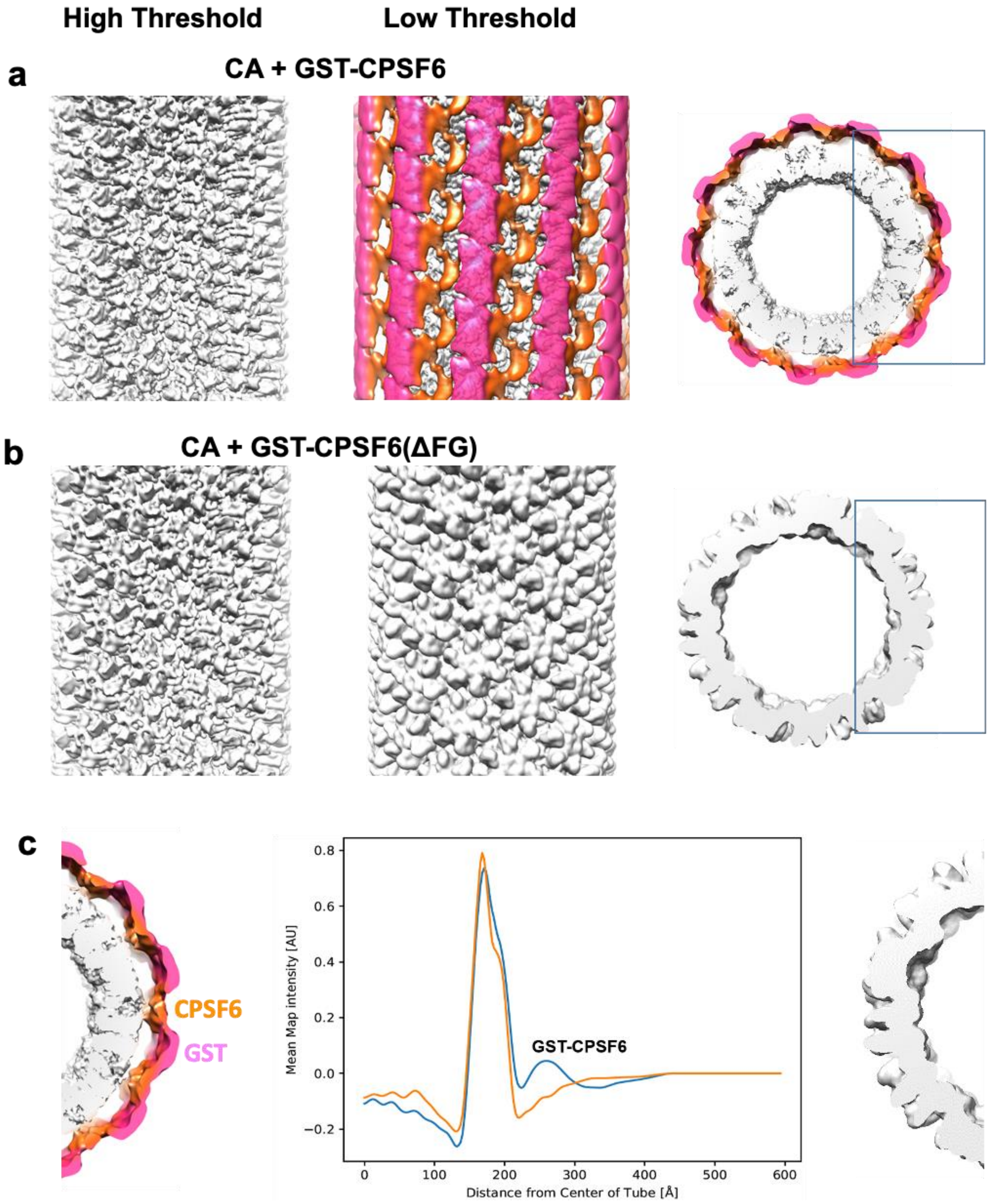

Supplementary Figure 10. Cryo-EM maps of IP6-stabilized CA tubes in the presence of GST-CPSF6$_{261\text{-}358}$ or GST-CPSF6$_{261\text{-}358}$(ΔFG). (a) Cryo-EM map at overall resolution of ~7.9 Å of a CA(A92E) tube in the presence of GST-CPSF6$_{261\text{-}358}$, displayed at a threshold where CA hexamer features are clearly discernible (High Threshold), and a lower threshold (Low Threshold) that allows for visualization of partially ordered density evident in cryo-images and 2D class averages. The map is colored by radius to facilitate interpretation. Additional density (in orange) is apparent along the interfaces between rows of hexamers in the CA nanotubes. Also apparent is density

(in magenta) that matches the size and overall shape to the X-ray structure of a GST dimer. (b) Cryo-EM map at overall resolution ~7.0 Å of a CA(A92E) nanotube following incubation with GST-CPSF6$_{261\text{-}358}$(ΔFG), displayed at a threshold where CA hexamer features are clearly discernible (High Threshold), and a much lower threshold (Low Threshold). The map is colored by radius as in (a) and the only feature beyond the CA nanotube surface is small random noise, even when the map is displayed at the lowest possible threshold (>0.0001). (c) Overlaying radial distribution density plots for CA + GST-CPSF6$_{261\text{-}358}$ vs CA + GST-CPSF6$_{261\text{-}358}$(ΔFG) shows the positioning and proposed identity of additional density specifically seen only in the complex with GST-CPSF6$_{261\text{-}358}$.

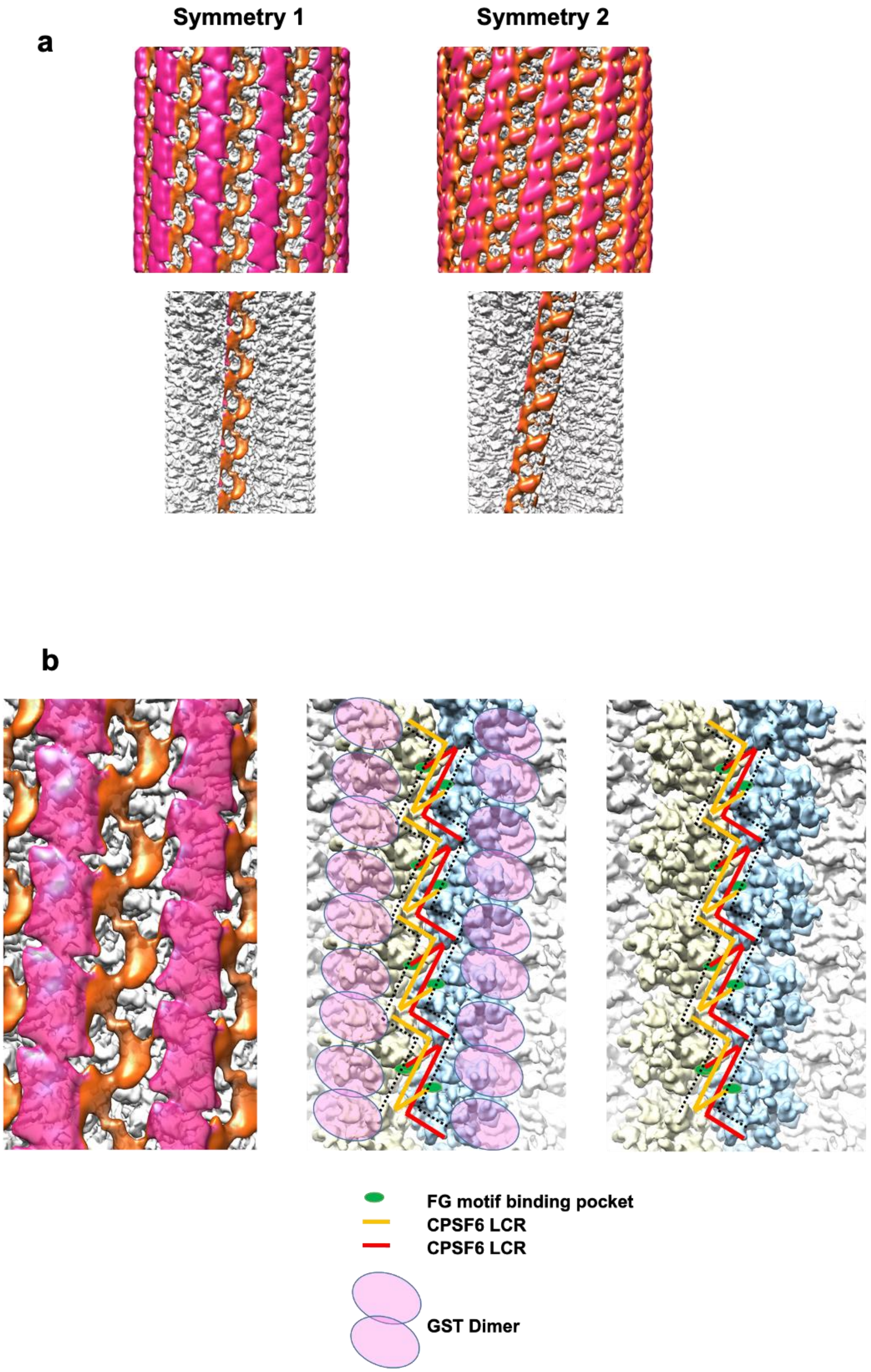
Symmetry 1
Symmetry 2
a
b
FG motif binding pocket
CPSF6 LCR
CPSF6 LCR
GST Dimer

Supplementary Figure 11. Cryo-EM analysis of CA + GST-CPSF6$_{261\text{-}358}$. (a) Comparison between two independent cryo-EM maps of CA + GST-CPSF6$_{261\text{-}358}$ calculated from CA tube with different helical symmetries. Top panels: in each map, zig-zagging CPSF6 densities (orange) extend between adjacent CA hexamer rows and are flanked by GST density (magenta). Bottom panels: slicing the maps to highlight CPSF6 density (orange) shows that the arrangement of CPFS6 is largely conserved in different symmetry maps; the only substantial difference is in the orientation of CPSF6 density due to the corresponding difference in helical symmetry between the CA nanotubes. (**b**) Schematic model to show multivalent assembly of CPSF6 templated by their interactions with the helical CA tube. The FG peptides (in light green) were used to set the register of plausible arrangements of CPSF6 LCRs (red and yellow lines) in the context of the density corresponding to CPSF6.

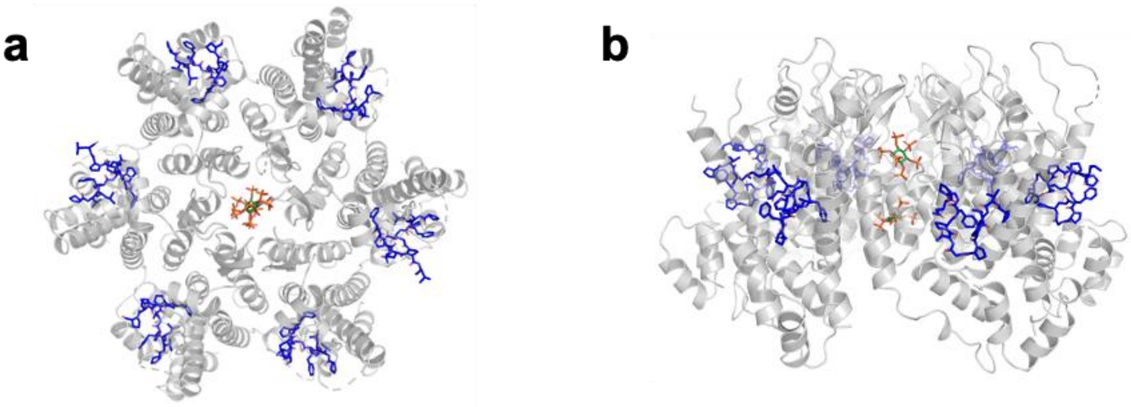

Supplementary Figure 12. The crystal structure of cross-linked CA hexamer in complex with IP6 and CPSF6$_{313–327}$ peptide. Top (**a**) and side (**b**) views of the 2.81 Å resolution crystal structure of cross-linked CA$_{A14C/E45C/W184A/M185A}$ hexamer (grey) in complex with IP6 (green) and CPSF6$_{313–327}$ (blue) (PDB: 7SNQ). Within the structure, the CPSF6$_{313–327}$ peptide engages with two adjacent CA subunits with a stoichiometry of six CPSF6$_{313–327}$ molecules per CA hexamer and two IP6 molecules bound near the ring of Arg18 and Lys25 residues per CA hexamer. Our crystal structure of the tripartite complex exhibits similarities with the published structures of CA hexamer + IP6 and CA hexamer + CPSF6$_{313–327}$ [1-4]. In common with the CA hexamer + IP6 structure [1], we observed that two IP6 molecules per CA hexamer were accommodated at nearly the same locations within the tripartite complex [1,2]. We also observed variations between the published CA hexamer + IP6 and our tripartite complex structures in positioning of CA residues 177-185, likely due to bound CPSF6$_{313–327}$ influencing the latter structure [1]. Similarly to reported CA hexamer + CPSF6$_{313–327}$ peptide structures [4], we observed that in the tripartite complex CPSF6$_{313–327}$ engages two adjacent CA subunits by interacting with the N-terminal hydrophobic pocket of one subunit and C-terminal Gln179 and Lys182 residues of the adjoining subunit.

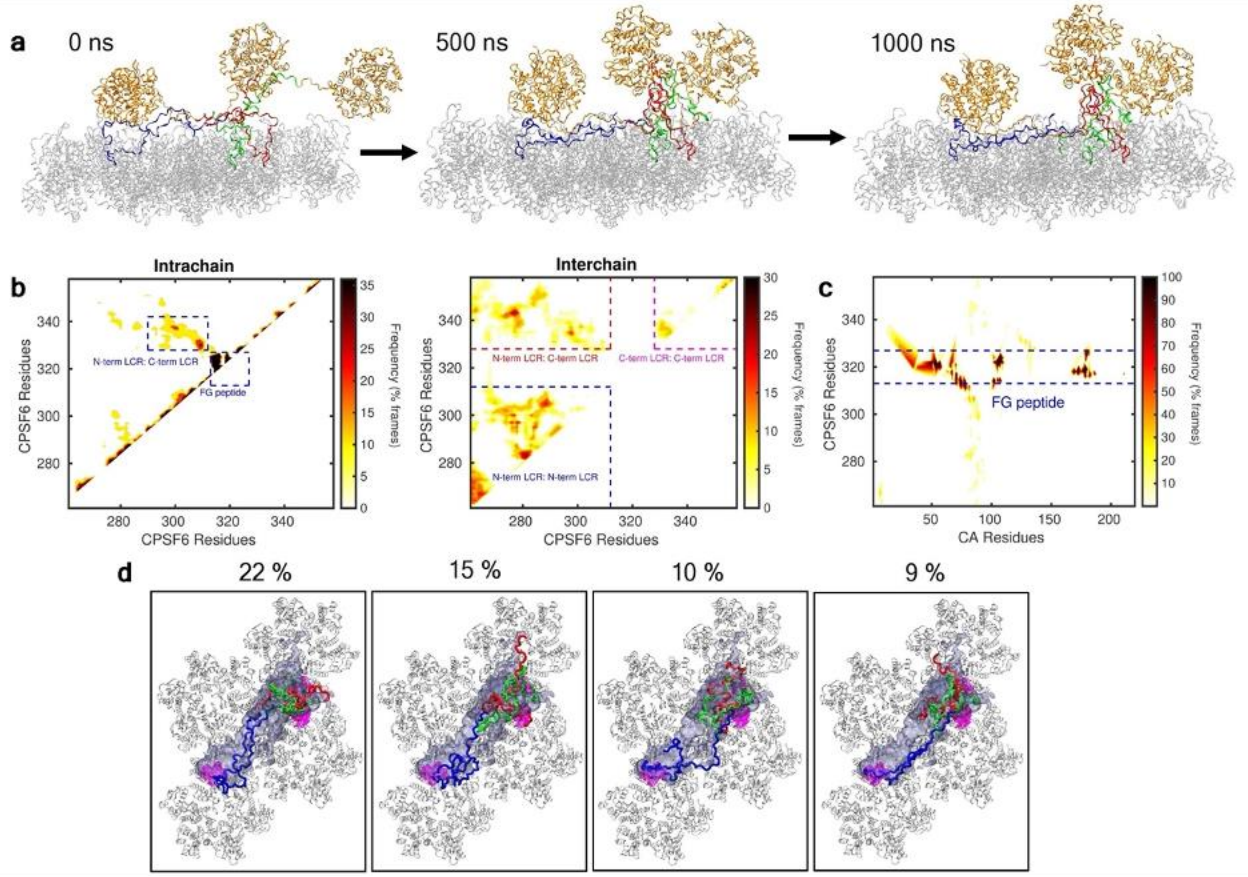

Supplementary Figure 13. AA MD simulations of CPSF6 assembly templated by CA hexamer lattice. (**a**) Initial model (left panel) of the GST-CPSF6$_{261\text{-}358}$ bound to the CA hexamer lattice. The center and right panels show the snapshot of the system at halfway (500 ns) and end (1000 ns) of the simulation trajectory. The CA hexamer and GST dimer is shown in silver and orange ribbons, respectively. The three CPSF6 chains are colored in blue, green, and red ribbons. (**b**) Time-averaged intrachain and interchain residue-residue contact map of the CPSF6 polypeptides. In the intrachain contact map, key contacts between the FG peptide residues, and N-terminal LCR and C-terminal LCR are highlighted. The interactions between the N-terminal LCR, N-terminal and C-terminal LCR, and C-terminal LCR segments are highlighted in the interchain contact maps. (**c**) Time-averaged residue-residue contact map between CPSF6 chains and CA hexamer lattice. The region bound by the dotted line highlights the interactions of the FG peptide with the CA residues. (**d**) The time-averaged density of the LCR fragment of the tri-CPSF6 complex (shown as faded blue surface) is overlaid onto the 4 CA hexamer lattice (shown in silver ribbons). The time-averaged density of the FG peptide for each CPSF6 chain is shown in solid magenta wireframe. The polypeptide densities are contoured at r = 0.01 and 0.005 for LCR and FG, respectively. Finally, the top four conformational clusters of the tri-CPSF6 complex are shown superimposed onto the 4 CA hexamer lattice, LCR, and FG peptide density. The title of each snapshot represents the percentage of simulation frames where the conformational clusters were observed.

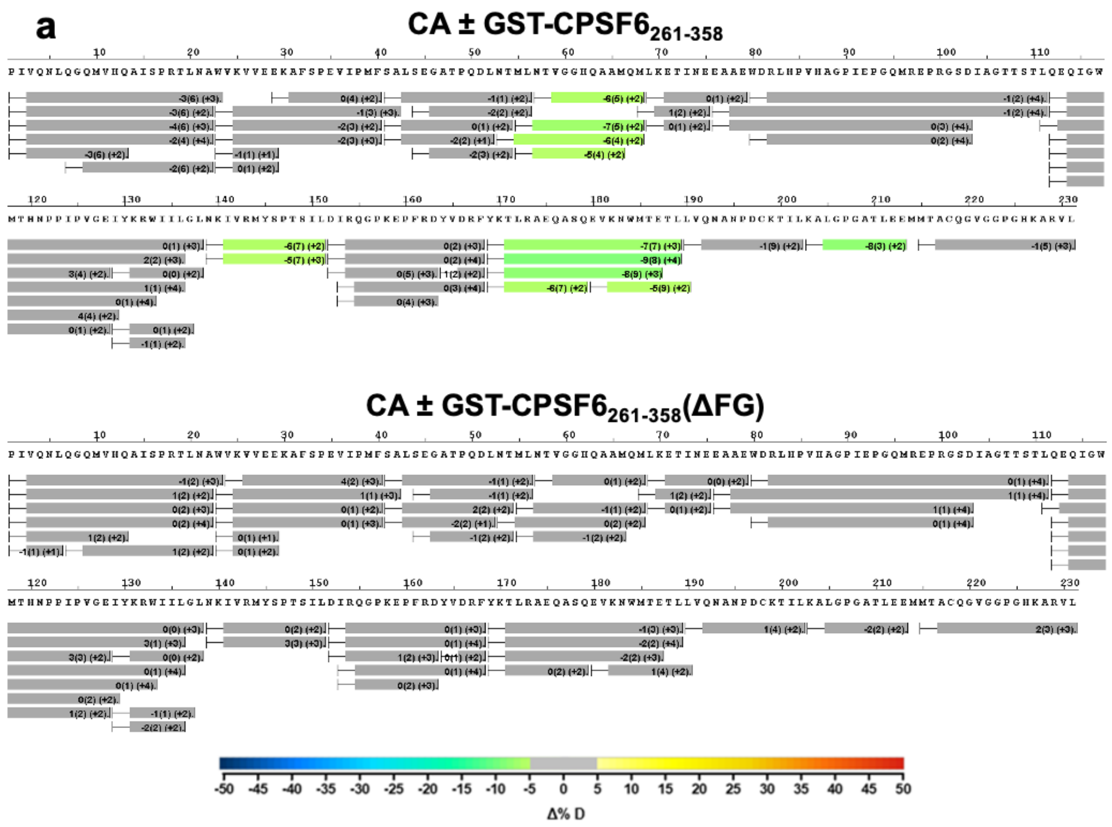
a
CA ± GST-CPSF6261-358
CA ± GST-CPSF6261-358(ΔFG)
Δ% D

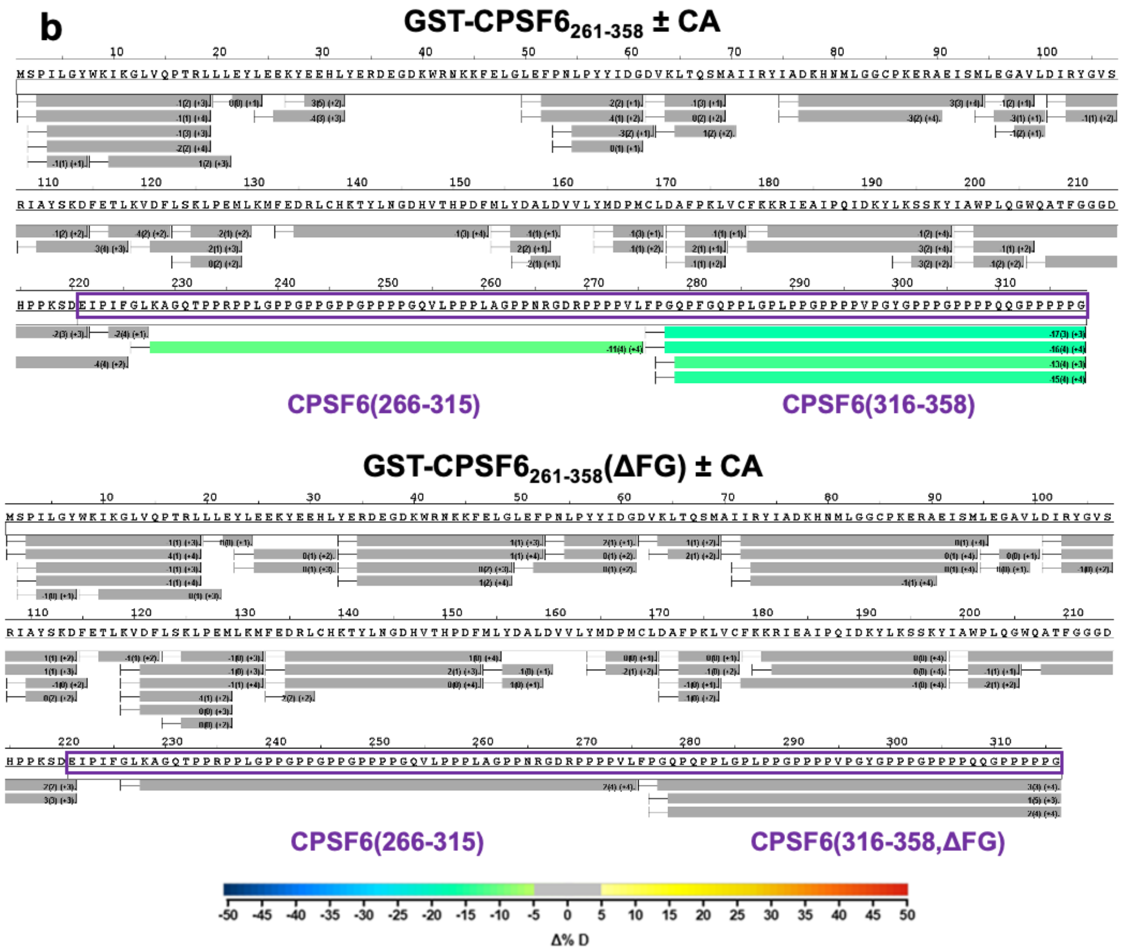
b
GST-CPSF6261-358 ± CA
CPSF6(266-315)
CPSF6(316-358)
GST-CPSF6261-358(ΔFG) ± CA
CPSF6(266-315)
CPSF6(316-358,ΔFG)
Δ% D

Supplementary Figure 14. Consolidated HDX-MS results. (**a**) Comparing protection in IP6 stabilized CA tubes upon addition of GST-CPSF6$_{261\text{-}358}$ vs GST-CPSF6$_{261\text{-}358}$(ΔFG). Detected peptide fragments from HIV-1 CA are shown under respective amino acid sequences. Coloring corresponds to HDX protection levels indicated by the heat map (gray: no protection; cold colors: protection; warm colors: deprotection). (**b**) Comparing protection in GST-CPSF6$_{261\text{-}358}$ vs CPSF6$_{261\text{-}358}$(ΔFG) upon addition of IP6-stabilized CA tubes. Detected peptide fragments from GST-CPSF6$_{261\text{-}358}$ and GST-CPSF6$_{261\text{-}358}$(ΔFG) are shown under respective amino acid sequences. Coloring corresponds to HDX protection levels indicated by the heat map (gray: no protection; cold colors: protection; warm colors: deprotection). The amino acid sequences corresponding to CPSF6$_{261\text{-}358}$ and CPSF6$_{261\text{-}358}$(ΔFG) are indicated by purple boxes. The amino acid numbering above indicated sequences corresponds to their positioning within the recombinant GST-CPSF6$_{261\text{-}358}$. GST-CPSF6$_{261\text{-}358}$ and GST-CPSF6$_{261\text{-}358}$(ΔFG) proteins analyzed. Numbering corresponding to CPSF6 sequences are indicated in purple under the detected peptide fragments.

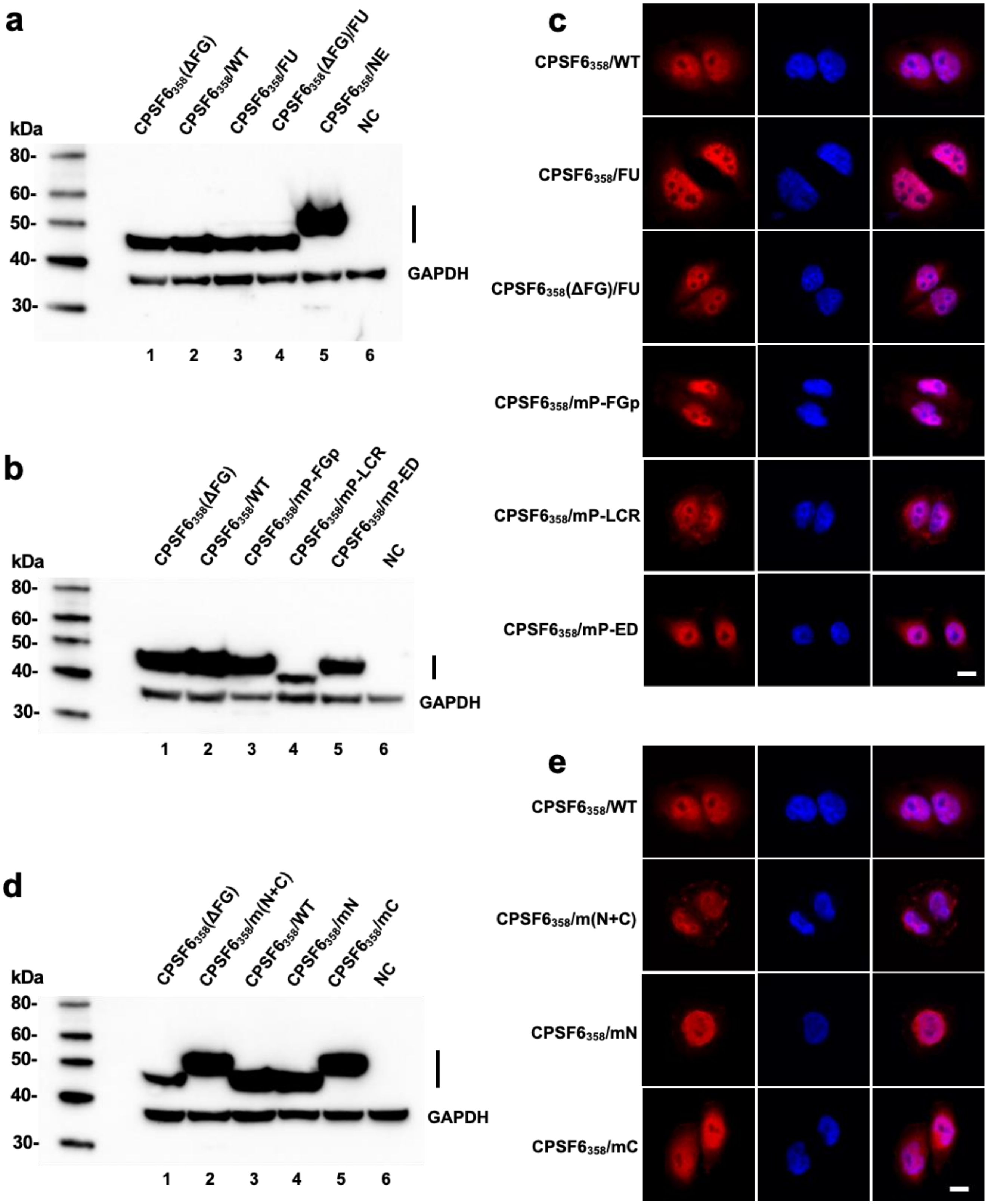

Supplementary Figure 15. Probing roles of CPSF6 LCRs. (**a, b**) Representative immunoblotting to show the expression of CPSF6$_{358}$ and mutant proteins used in **Fig. 7.** (**c**) Immunofluorescence to detect the CPSF6$_{358}$ and mutant proteins used in Fig. 7. HeLa cells were immunostained for HA-tagged protein (red) and nuclei DAPI (blue). Scale bar is 5 µm. (**d**) Representative immunoblotting to show the expression of WT CPSF6$_{358}$ and related mutant proteins used in Fig. 8. Expression of target proteins are indicated by a side bar. (**e**) Immunofluorescence to detect

the WT $CPSF6_{358}$ and related mutant proteins used in Fig. 8. Hela cells were immunostained for HA-tagged protein (red) and nuclei DAPI (blue). Scale bar is 5 µm. All the experiments were repeated 3 times independently with similar results. Abbreviation: FU: FUS, NE: NEURM, CKO: CPSF6 knock-out, LCR: low complexity region, a.u.:arbitrary units

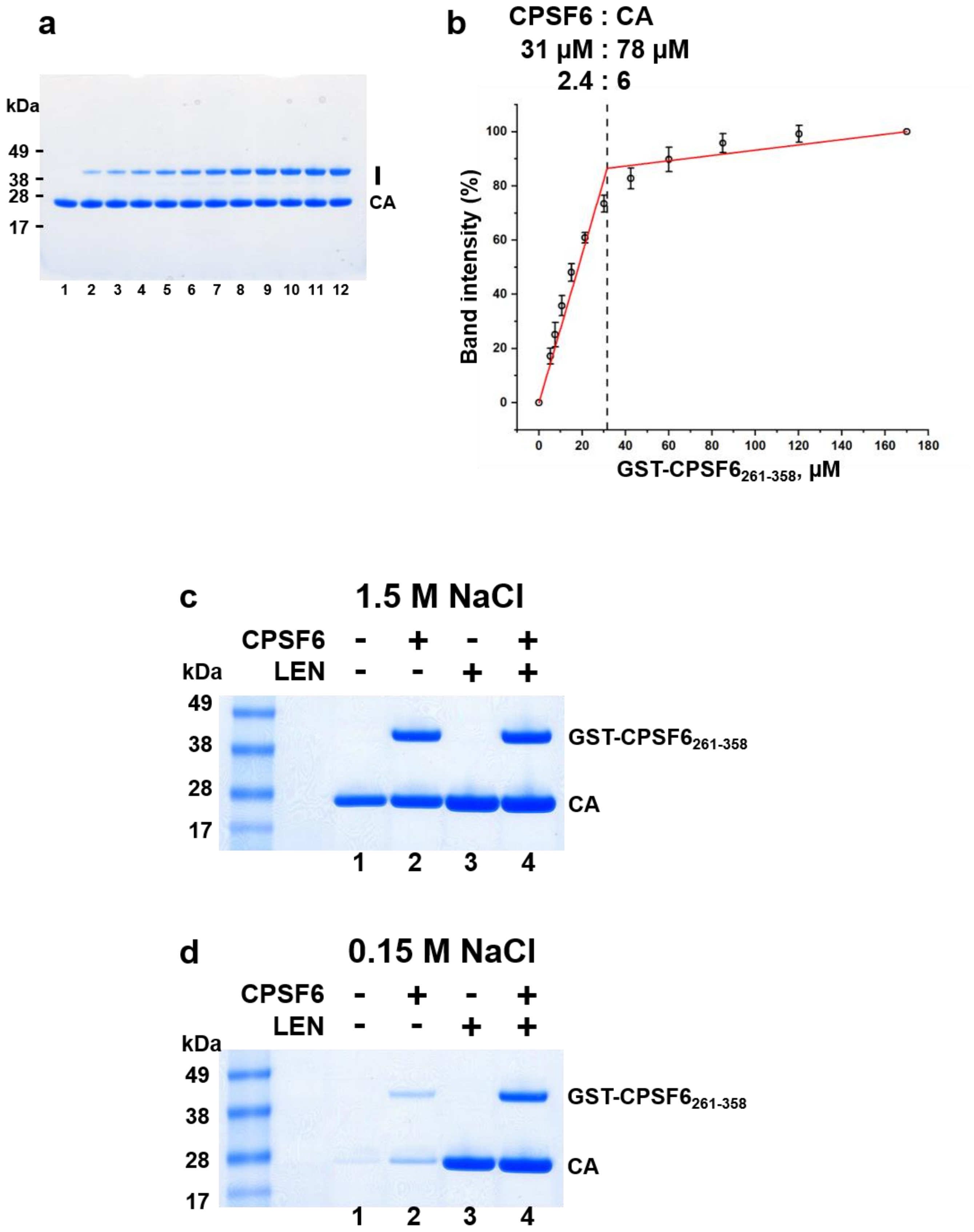

Supplementary Figure 16. Biochemical analysis of CPSF6 and LEN interactions with CA nanotubes. (**a**,**b**) Quantitative analysis of GST-CPSF6$_{261-358}$ binding to CA tubes. (**a**) Representative SDS-PAGE image. Pre-formed

CA nanotubes (78 μM) were incubated with indicated concentrations of GST-CPSF6$_{261\text{-}358}$. (**b**) Quantification of results in (**a**). The averaged data (+/− SD) from three independent experiments are shown. Source data are provided as a Source Data file. (**c**) CA nanotubes were assembled in 1.5 M NaCl, incubated in the absence (lane 1) or presence of GST-CPSF6$_{261\text{-}358}$ (lane 2) and then pelleted. LEN was added to CA nanotubes or CA nanotubes + GST-CPSF6$_{261\text{-}358}$ and then pelleted. (**d**) Lanes 1 and 2: CA nanotubes were assembled in 1.5 M NaCl, incubated in the absence or presence of GST-CPSF6$_{261\text{-}358}$ (similarly to lanes 1 and 2 shown above in (**c**), pelleted, then re-suspended in the 0.15 M NaCl containing buffer followed by pelleting. Lanes 3 and 4: CA nanotubes were assembled in 1.5 M NaCl, incubated in the absence or presence of GST-CPSF6$_{261\text{-}358}$ (similarly to lanes 3 and 4 shown above in (**c**), pelleted, then re-suspended in the 0.15 M NaCl containing buffer followed by pelleting. Equimolar concentrations (50 μM) of CA, GST-CPSF6$_{261\text{-}358}$ and LEN were used in **c** and **d**. Representative SDS-PAGE images of pelleted fractions of five independent experiments are shown. Abbreviation: LEN: lenacapavir.

Supplementary Table 1. Binding affinity of $CPSF6_{313–327}$, $NUP153_{1409–1423}$, and $SEC24C_{228–242}$ peptides to cross-linked CA hexamer.

**Peptides**	***K_d* (μM)**
$CPSF6_{313-327}$	70.9 ± 2.5
$NUP153_{1409-1423}$	131 ± 13
$SEC24C_{228-242}$	1070 ± 101

Supplementary Table 2. Integration site numbers and distributions with respect to SPADs, average gene number/Mb, LADs, and Refseq genes.

Genotype	Total Integration Sites	SPADs (%)	Avg. Gene number/Mb	LAD (%)	RefSeq (%)
CKO	4,191	1.0<br>*p*=1.6E-200*<br>*p*=1.2E-16**	6.6<br>*p*=2.8E-267<br>*p*=9.1E-15	58.9<br>*p*=4.2E-165<br>*p*=9.0E-23	60.3<br>*p*=4.6E-51<br>*p*=2.5E-83
CKO + CPSF6/WT	4,476	18.7<br>*p*<E-300	15.4<br>*p*<E-300	29.9<br>*p*=1.8E-176	75.3<br>*p*<E-300
CKO + CPSF6/AD	5,910	1.3<br>*p*=1.7E-231<br>*p*=6.4E-15	6.5<br>*p*<E-300<br>*p*=2.5E-28	59<br>*p*=2.9E-194<br>*p*=1.0E-31	57.4<br>*p*=6.8E-81<br>*p*=2.1E-77
CKO + CPSF6/NE	51,933	0.5<br>*p*<E-300<br>*p*=1.5E-257	6.4<br>*p*<E-300<br>*p*=1.8E-127	60.2<br>*p*<E-300<br>*p*=4.4E-258	62.0<br>*p*=6.3E-75<br>*p*<E-300
CKO + CPSF6/FU	3,918	21.9<br>*p*=2.9E-04<br>*p*<E-300	17.4<br>*p*=1.8E-15<br>*p*<E-300	22.8<br>*p*=1.2E-13<br>*p*=8.7E-283	79.2<br>*p*=3.5E-05<br>*p*<E-300
CKO + CPSF6/CD	24,140	17.9<br>*p*=4.1E-01<br>*p*<E-300	15.1<br>*p*=6.7E-02<br>*p*<E-300	29<br>*p*=7.1E-01<br>*p*<E-300	77.3<br>*p*=1.9E-06<br>*p*<E-300
RIC	112,183	2.8	7.9	51.2	45.1

P values were calculated by two-sided Fisher's exact text or by two-sided Wilcoxon-Mann-Whitney rank sum test (for gene density). No adjustment was made for multiple comparison.
* *P* values in blue indicate differences compared to CPSF6/WT control.
** *P* values in black indicate differences compared to RIC.

Supplementary Table 3. Cryo-EM data collection statistics.

	CA+IP6+ GST-CPSF6 EMD-27617	CA+IP6+ GST-CPSF6 EMD-27619	CA+IP6+ GST-CPSF6(ΔFG) EMD-27625
Data collection and processing:	Symmetry 1	Symmetry 2	
Microscope	Arctica (ThermoFisher)	Arctica (ThermoFisher)	Arctica (ThermoFisher)
Voltage (keV)	200	200	200
Camera	K3 Summit (Gatan)	K3 Summit (Gatan)	K3 Summit (Gatan)
Magnification	28,000 X	28,000 X	28,000 X
Pixel size at detector (Å/pixel)	1.4	1.4	1.4
Total electron exposure (e^-/Å2)	45.65	45.65	46.15
Exposure rate (e^-/pixel/sec)	29.829	29.829	30.153
Number of frames collected during exposure	40	40	40
Defocus range (µm)	1.5-2.5	1.5-2.5	1.5-2.5
Micrographs collected (no.)	4,706	4,706	2,157
Micrographs used (no.)	530	569	491
Total extracted tube segments (no.)	236,974	236,974	109,696
Reconstruction:			
Refined particles (no.)	5,894	6,212	3,023
Final particles (no.)	5,894	6,212	3,023
Helical symmetry parameters (twist (°), rise (Å))	-55.2834, 7.12159	138.157, 7.06355	82.5822, 6.95455
Resolution (global, Å)	7.9	7.4	7.0
FSC 0.143 (masked)	7.9	7.4	7.0
Map sharpening B factor ($Å^2$) /(B factor Range)	-614	-544	-385
Map sharpening method	Relion postprocess	Relion postprocess	Relion postprocess

Supplementary Table 4. Summary of X-ray data collection and refinement statistics.

	CA_{hex} + IP_6 + $CPSF6_{313-327}$<br><br>PDB ID: 7SNQ
**Data collection**	
X-ray Source	ALS 4.2.2
Software	XDS
Wavelength	1.0000 Å
Space group	P 2_1 2_1 2_1
Unit cell dimensions	
a, *b*, *c* (Å)	133.92, 135.67, 212.88
α, β, γ (°)	90, 90, 90
Resolution (Å)	45.22 – 2.81 (2.86-2.81)
No. total reflections	611,743 (26,623)
No. unique reflections	94,907 (4,661)
R_{merge}	0.083 (1.191)
CC1/2	0.999 (0.576)
$I / \sigma I$	16.5 (1.4)
Completeness (%)	99.9 (100)
Redundancy	6.4 (6.1)
**Refinement**	
Resolution (Å)	44.24 – 2.81 (2.91–2.81)
No. reflections used in refinement	94,235 (9,361)
No. reflections used for R_{free}	4,738 (465)
R_{work} (%)	22.90 (33.03)
R_{free} (%)	28.18 (38.48)
No. non-hydrogen atoms	20,569
Protein	20,252
Ligand/ion	146
Water	171
Wilson B-factor	69.97
Average B-factors	85.16
Protein	84.78
Ligands/ions	163.77
Waters	62.15
R.m.s. deviations	
Bond lengths (Å)	0.009
Bond angles (°)	1.09
Ramachandran	
Favored (%)	97.54
Allowed (%)	2.46
Outliers (%)	0
Rotamer outliers (%)	0.28
Clashscore	7.60

The values in parentheses indicate the highest resolution shell.

Supplementary Table 5. HDX-MS experimental conditions and data analysis parameters from the guidelines of the IC-HDX-MS community.

**Data set for Fig 6 and Supplementary Fig 14**	**GST-CPSF6$_{261\text{-}358}$ in the presence of CA(A92E)**
HDX reaction details	100 mM MOPS, 1 M NaCl, 200 µM IP6, pH = 6.5, 2min, 25 °C
HDX quench reaction details	0.1 M Na Phosphate, 50 mM TCEP, pH = 2.0, 4 °C
HDX time course (sec)	10 and 30
Back-exchange	estimated from input recovery estimate of 0.7 and deuterium solution concentration of 0.8
# of Peptides	50
Sequence coverage	92 %
Average peptide length / Redundancy	5 / 2.414
Replicates (biological or technical)	2 biological replicates with 3 technical replicates each
Repeatability	2.730739 (average STD)
Significant differences in HDX	> 5 %D (unpaired t-tests at each time point, p-value < 0.01)
**Data set for Fig 6 and Supplementary Fig 14**	**GST-CPSF6$_{261\text{-}358}$(ΔFG) in the presence of CA(A92E)**
HDX reaction details	100 mM MOPS, 1 M NaCl, 200 µM IP6, pH = 6.5, 2min, 25 °C
HDX quench reaction details	0.1 M Na Phosphate, 50 mM TCEP, pH = 2.0, 4 °C
HDX time course (sec)	10 and 30
Back-exchange	estimated from input recovery estimate of 0.7 and deuterium solution concentration of 0.8
# of Peptides	59
Sequence coverage	98%
Average peptide length / Redundancy	12 / 2.845
Replicates (biological or technical)	2 biological replicates with 3 technical replicates each
Repeatability	1.298794 (average STD)
Significant differences in HDX	> 5 %D (unpaired t-tests at each time point, p-value < 0.01)
**Data set for Fig 6 and Supplementary Fig 14**	**CA(A92E) in the presence of GST-CPSF6$_{261\text{-}358}$**
HDX reaction details	100 mM MOPS, 1 M NaCl, 200 µM IP6, pH = 6.5, 2min, 25 °C
HDX quench reaction details	0.1 M Na Phosphate, 50 mM TCEP, pH = 2.0, 4 °C
HDX time course (sec)	10, 30, 60, 900, 3600
Back-exchange	estimated from input recovery estimate of 0.7 and deuterium solution concentration of 0.8
# of Peptides	54
Sequence coverage	99%
Average peptide length / Redundancy	8 / 3.796
Replicates (biological or technical)	2 biological replicates with 3 technical replicates each
Repeatability	5.081573 (average STD)
Significant differences in HDX	> 5 %D (unpaired t-tests at each time point, p-value < 0.01)
**Data set for Fig 6 and Supplementary Fig 14**	**CA(A92E) in the presence of GST-CPSF6$_{261\text{-}358}$(ΔFG)**
HDX reaction details	100 mM MOPS, 1 M NaCl, 200 µM IP6, pH = 6.5, 2min, 25 °C
HDX quench reaction details	0.1 M Na Phosphate, 50 mM TCEP, pH = 2.0, 4 °C
HDX time course (sec)	10, 30, 60, 900, 3600
Back-exchange	estimated from input recovery estimate of 0.7 and deuterium solution concentration of 0.8
# of Peptides	54
Sequence coverage	99%
Average peptide length / Redundancy	8 / 3.817
Replicates (biological or technical)	2 biological replicates with 3 technical replicates each
Repeatability	2.222696 (average STD)
Significant differences in HDX	> 5 %D (unpaired t-tests at each time point, p-value < 0.01)

Supplementary Table 6. Sequences of the primers used for cloning.

PRIMER NAME	SEQUENCE (5' TO 3')
TSIN-CPSF6-F	CTCCATAGAAGACACCGACTCTAGAGGATCCATGGCGGACGGCGTGGACCAC
TSIN-CPSF6-R	AGGTACCGGGCCCCCCCTCGAGGTTAAGCGTAATCTGGAACATCGTATGGGTAGCCTGGAGGTGGAGGTGGTCC
TSIN-FUS-R	ACTCTAGAGGTACCGGGCCCCCCCTCGAGGTTAAGCGTAATCTGGAACATCGTATGGGTAAGAACTGCTACCGTAACTTCC
TSIN-ADD2-R	ACTCTAGAGGTACCGGGCCCCCCCTCGAGGTTAAGCGTAATCTGGAACATCGTATGGGTAGAGGATTTCCTCTGCCGTCTG
TSIN-NEURM-R	ACTCTAGAGGTACCGGGCCCCCCCTCGAGGTTAAGCGTAATCTGGAACATCGTATGGGTAGGCACTTTCTGTCTCAGCTGAG
TSIN-C13-1R	GATCTCCTCGATTAGGAGGCCCGGGCTCATCAGGCTTGGAGCC
TSIN-C13-2F	GGCTCCAAGCCTGATGAGCCCGGGCCTCCTAATCGAGGAGATC
TSIN-C14-1R	GAGGAGGCAGAACCTGACCAGGGGCTGCTTCGGCAGTAGTGGTG
TSIN-C14-2F	CACCACTACTGCCGAAGCAGCCCCTGGTCAGGTTCTGCCTCCTC
TSIN-C15-1R	GAGGAGGCAGAACCTGACCAGGCTCTATGCTGTGCCCCATATG
TSIN-C15-2F	CATATGGGGCACAGCATAGAGCCTGGTCAGGTTCTGCCTCCTC
TSIN-C15-2R	ACTCTAGAGGTACCGGGCCCCCCCTCGAGGTTAAGCGTAATCTGGAACATCGTATGGGTAACCCAATGGAGGCTGCCCAAAAG
TSIN-C16-1R	GAGGAGGGGACTGAGTGGCCAAAGGTGGAGGACCTGGTGGACC
TSIN-C16-2F	CCACCTTTGGCCACTCAGTCCCCTCCTCCTCTAGCTGGGCCTC
TSIN-C17-1R	GGAGGCCCGGACTGAGTGGCCAACAGAACCTGACCAGGAGGTGGAG
TSIN-C17-2F	CAGGTTCTGTTGGCCACTCAGTCCGGGCCTCCTAATCGAGGAGATC
TSIN-C18-1R	TCTCCGGACTGAGTGGCCAAAGCTAGAGGAGGAGGCAGAAC
TSIN-C18-2F	TCTAGCTTTGGCCACTCAGTCCGGAGATCGCCCTCCACCACCAG
TSIN-C19-1R	CTGGGTAGGACTGAGTGGCCAATCGATTAGGAGGCCCAGCTAG
TSIN-C19-2F	ATCGATTGGCCACTCAGTCCTACCCAGTTCTTTTTCCTGGACAAC
TSIN-C20-1R	AGAGCTCCCAGCTAGAGAAGAGCTCAGAACCTGACCGCTAGGTGGAGGACCTGGTGGACC
TSIN-C20-2F	GAGCTCTTCTCTAGCTGGGAGCTCTAATCGAGGAGATCGCAGCTCTAGCGTTCTTTTTCCTGGACAACCTTTTG
TSIN-C21-1R	GCTAGGAGGGCTATAGCTAGGAGGAGGATAGCTATAGCTAGGAGGTGGAGGACCTGGTGGAC
TSIN-C21-2F	CCTCCTCCTAGCTATAGCCCTCCTAGCTATAGCTATAGCCCTCCACCACCAGTTCTTTTTCCTGGACAAC
TSIN-DELTAFG-1R	GAAGTGGACCCAATGGAGGCTGAGGTTGTCCAGGAAAAAGAACTG
TSIN-DELTAFG-2F	CAGTTCTTTTTCCTGGACAACCTCAGCCTCCATTGGGTCCACTTCCTC
TSIN-C23-1R	AAATGGATATTTCCTCTATGCTGTGCCCCATATGC
TSIN-C23-2F	GCACAGCATAGAGGAAATATCCATTTTTGGCCTAAAAG
TSIN-C23-2R	CGACTCTAGAGGTACCGGGCCCCCCCTCGAGGTTAAGCGTAATCTGGAACATCGTATG
TSIN-C24-1R	AAAATGTCTATTTCCTCTATGCTGTGCCCCATATGC

Supplementary References:

1 Dick, R. A. *et al.* Inositol phosphates are assembly co-factors for HIV-1. *Nature* **560**, 509-512, doi:10.1038/s41586-018-0396-4 (2018).

2 Mallery, D. L. *et al.* IP6 is an HIV pocket factor that prevents capsid collapse and promotes DNA synthesis. *Elife* **7**, doi:10.7554/eLife.35335 (2018).

3 Bhattacharya, A. *et al.* Structural basis of HIV-1 capsid recognition by PF74 and CPSF6. *Proc Natl Acad Sci U S A* **111**, 18625-18630, doi:10.1073/pnas.1419945112 (2014).

4 Price, A. J. *et al.* Host cofactors and pharmacologic ligands share an essential interface in HIV-1 capsid that is lost upon disassembly. *PLoS Pathog* **10**, e1004459, doi:10.1371/journal.ppat.1004459 (2014).
