# Supplementary material for: Prion-like low complexity regions enable avid virus-host interactions during HIV-1 infection
Source: Nat Commun. 2022 Oct 6;13:5879. doi: 10.1038/s41467-022-33662-6 (PMC9537594; doi:10.1038/s41467-022-33662-6)
Supplement: Supplementary file 2 — Reporting Summary [file 41467_2022_33662_MOESM2_ESM.pdf]

nature portfolio

Corresponding author(s): Dr. Mamuka Kvaratskhelia

Last updated by author(s): Aug 29, 2022

Reporting Summary

Nature Portfolio wishes to improve the reproducibility of the work that we publish. This form provides structure for consistency and transparency in reporting. For further information on Nature Portfolio policies, see our Editorial Policies and the Editorial Policy Checklist.

Statistics

For all statistical analyses, confirm that the following items are present in the figure legend, table legend, main text, or Methods section.

n/a	Confirmed	
☐	☒	The exact sample size (n) for each experimental group/condition, given as a discrete number and unit of measurement
☐	☒	A statement on whether measurements were taken from distinct samples or whether the same sample was measured repeatedly
☐	☒	The statistical test(s) used AND whether they are one- or two-sided<br>*Only common tests should be described solely by name; describe more complex techniques in the Methods section.*
☒	☐	A description of all covariates tested
☐	☒	A description of any assumptions or corrections, such as tests of normality and adjustment for multiple comparisons
☐	☒	A full description of the statistical parameters including central tendency (e.g. means) or other basic estimates (e.g. regression coefficient) AND variation (e.g. standard deviation) or associated estimates of uncertainty (e.g. confidence intervals)
☐	☒	For null hypothesis testing, the test statistic (e.g. F, t, r) with confidence intervals, effect sizes, degrees of freedom and P value noted<br>*Give P values as exact values whenever suitable.*
☒	☐	For Bayesian analysis, information on the choice of priors and Markov chain Monte Carlo settings
☒	☐	For hierarchical and complex designs, identification of the appropriate level for tests and full reporting of outcomes
☒	☐	Estimates of effect sizes (e.g. Cohen's d, Pearson's r), indicating how they were calculated

Our web collection on statistics for biologists contains articles on many of the points above.

Software and code

Policy information about availability of computer code

Data collection	Carl Zeiss MicroImaging Zen software Black Version (2.3 SP1) was used to acquire fluorescent images<br>XDS Suite Build 20190315 was used to collect and process x-ray crystallographic data<br>Relion 3.1.2 was used to collect Cyro-EM data.<br>HDX Data was collected on a Q Exactive mass spectrometer from ThermoFisher using Xcalibur Software (2.2 SP1.48) from ThermoFisher
Data analysis	ICY Image analysis software (2.0.2.0) was used to determine amounts of PLA puncta in cells<br>Enspire manager (3.10.3005.1440) was used to analyze protein concentrations<br>Nanodrop-1000 (v3.8.1) was used to analyze DNA concentrations<br>Phaser and phenix.refine within Phenix Suite build 1.15.2-3472 were utilized for molecular replacement and x-ray crystal structure refinement, respectively.<br>Coot build 0.8.9.2 was used for manual refinement of the x-ray crystal structure<br>Origin 2019 (9.6) software was was used to analyze EC50 value<br>Integration sites were analyzed by BEDtools (2.30.0)<br>Surface Plasmon Resonance data was analyzed using Scrubber 2.0<br>Leginon 3.3 was used to analyze cyro-EM data.<br>Peptide identification completed using Mascot (version 2.3)(https://www.matrixscience.com/)<br>Differential HDX analysis completed using HDX Workbench(4.8.90) (http://hdxworkbench.com/)<br>MatchMaker was used to align FG peptide to crystallogrphic structure (version 1.16)<br>The all-atom (AA) molecular dynamics (MD) were performed with GROMACS software version gromacs/2019.4.<br>Visualization of the AA MD trajectories, figure preparation, and density map calculation of CPSF6 filaments was done with VMD visualization

software version vmd/1.9.3.
DED frame stack was motion corrected using MotionCor2 (1.5.0)

For manuscripts utilizing custom algorithms or software that are central to the research but not yet described in published literature, software must be made available to editors and reviewers. We strongly encourage code deposition in a community repository (e.g. GitHub). See the Nature Portfolio guidelines for submitting code & software for further information.

Data

Policy information about availability of data

All manuscripts must include a data availability statement. This statement should provide the following information, where applicable:

- Accession codes, unique identifiers, or web links for publicly available datasets
- A description of any restrictions on data availability
- For clinical datasets or third party data, please ensure that the statement adheres to our policy

All processed data is available in the manuscript or the supplementary materials. All raw data is available in the file of source data. The Raw integration site sequencing results are deposited at the National Center for Biotechnology Sequences Read Archive with accession number PRJNA787708 [https://www.ncbi.nlm.nih.gov/bioproject/PRJNA787708]. The coordinates of the crystal structure of CAhex + IP6 + CPSF6(313-327) are deposited in the Protein Data Bank under accession code: 7SNQ [https://doi.org/10.2210/pdb7SNQ/pdb]. Cryo-EM maps of CA + IP6 + GST-CPSF6(261-358) and CA + IP6 + GST-CPSF6(261-358, ΔFG) are deposited with EMDB codes EMD-27617 [https://www.ebi.ac.uk/emdb/EMD-27617], EMD-27619 [https://www.ebi.ac.uk/emdb/EMD-27619] and EMD-27625 [https://www.ebi.ac.uk/emdb/EMD-27625]. HDX MS results are deposited to the ProteomeXchange Consortium via the PRIDE partner repository with the data set identifier PXD030332 [https://www.ebi.ac.uk/pride/archive/projects/PXD030332].

Human research participants

Policy information about studies involving human research participants and Sex and Gender in Research.

Reporting on sex and gender	NA
Population characteristics	NA
Recruitment	NA
Ethics oversight	NA

Note that full information on the approval of the study protocol must also be provided in the manuscript.

Field-specific reporting

Please select the one below that is the best fit for your research. If you are not sure, read the appropriate sections before making your selection.

☒ Life sciences ☐ Behavioural & social sciences ☐ Ecological, evolutionary & environmental sciences

For a reference copy of the document with all sections, see nature.com/documents/nr-reporting-summary-flat.pdf

Life sciences study design

All studies must disclose on these points even when the disclosure is negative.

Sample size	All experiments in this study were performed at least in triplicates with reproducible data. Sample sizes were determined based on the authors experience (Bester et al, Science, 2020; Rebensburg et al, Nature microbiology, 2021) and standard generally accepted in the field of virology (Adarsh et al, Nature microbiology, 2020) to generate reliable results.
Data exclusions	Data were not excluded from analysis.
Replication	Each experiment was reproduced at least three times with similar results. All attempts at replication were successful.
Randomization	Bacterial colonies from transformations were selected randomly. The cells for microscopy assay were chosen randomly from different views. Randomization is not relevant to other experiments because samples are allocated to experimental groups based on differences that are intrinsic to the samples
Blinding	None of the data was blinded as data were quantitative and measurements were made using relevant instruments and software.

Reporting for specific materials, systems and methods

We require information from authors about some types of materials, experimental systems and methods used in many studies. Here, indicate whether each material, system or method listed is relevant to your study. If you are not sure if a list item applies to your research, read the appropriate section before selecting a response.

Materials & experimental systems

n/a	Involved in the study
☐	☒ Antibodies
☐	☒ Eukaryotic cell lines
☒	☐ Palaeontology and archaeology
☒	☐ Animals and other organisms
☒	☐ Clinical data
☒	☐ Dual use research of concern

Methods

n/a	Involved in the study
☒	☐ ChIP-seq
☒	☐ Flow cytometry
☒	☐ MRI-based neuroimaging

Antibodies

Antibodies used

anti-HIV-1 p24 monoclonal antibody (AG 3.0)(ARP-4121, NIH AIDS Reagent Program)
anti-HIV-1 p24 antibody (ab32352, Abcam)
anti-HA antibody [EPR22819-101] (ab236632, Abcam)
anti-HA antibody (NB600-362, Novus bio)
anti-Flag antibody (M2)(F1804, Sigma)
anti-GST antibody (8-326) (MA4-004-HRP, Thermofisher)
anti-CPSF6 antibody [EPR12898](ab175237, Abcam)
anti-Sec24C antibody (ab122633, Abcam)
anti-Nup153 antibody (NB100-93329, Novus)
anti-GAPDH antibody (6C5)(sc-47724, Santa Cruz)
goat anti-rabbit IgG (H+L) secondary antibody (65-6120, Invitrogen)
goat anti-mouse IgG (H+L) secondary antibody conjugated (65-6520, Invitrogen)
Anti-SC35 antibody [SC-35] (ab11826, abcam)
Anti-SON Antibody (HPA031755, Atlas antibodies)
Goat anti-rabbit-AlexaFluor 405 (A-31556, ThermoFIsher)

Validation

All antibodies except anti-HIV-1 p24 monoclonal antibody were obtained commercially and were validated by respective vendors.
Anti-HIV-1 p24 monoclonal antibody (AG 3.0) was obtained from NIH AIDS Reagent Program and validated as reported (Francis et al, Plos Pathogen, 2016; Francis and Melikyan, Cell host&microbe 2018; Rebensburg et al, Nature microbiology, 2021).
anti-HIV-1 p55+p24+p17 antibody (ab63917, Abcam): Rabbit polyclonal antibody to HIV1 p55 + p24 + p17 suitable for WB and ELISA https://www.abcam.com/hiv1-p55--p24--p17-antibody-ab63917.html
anti-HA antibody [EPR22819-101] (ab236632, Abcam): Rabbit monoclonal antibody to HA tag suitable for Flow Cyt (Intra), WB, IP, ICC/IF. https://www.abcam.com/ha-tag-antibody-epr22819-101-ab236632.html
anti-HA antibody (NB600-362, Novus bio): Goat polyclonal antibody to HA tag suitable for WB, ELISA, ICC/IF, IHC, IP. https://www.novusbio.com/products/ha-tag-antibody_nb600-362
anti-Flag antibody (M2)(F1804, Sigma):mouse monoclonal antibody to Flag tag suitable for WB, ICC/IF, IHC, IP. https://www.sigmaaldrich.com/US/en/product/sigma/f1804
anti-GST antibody ((8-326) (MA4-004-HRP, Thermofisher): Mouse monoclonal antibody to GST tag suitable for WB and ICC/IF. https://www.thermofisher.com/antibody/product/GST-Tag-Antibody-clone-8-326-Monoclonal/MA4-004-HRP
anti-CPSF6 antibody [EPR12898](ab175237, Abcam): Rabbit monoclonal antibody to mouse, rat and human CPSF6 suitable for ICC/IF, Flow Cytometry, IP, IHC-P, WB. https://www.abcam.com/cpsf6-antibody-epr12898-ab175237.html
anti-Sec24C antibody (ab122633, abcam): Rabbit polyclonal antibody to human Sec24C suitable for IHC-P, ICC/IF, WB. https://www.abcam.com/sec24c-antibody-ab122633.html
anti-Nup153 antibody (NB100-93329, Novus): Rabbit polyclonal antibody to human Nup153 suitable for WB, ICC/IF, IP and KO https://www.novusbio.com/products/nup153-antibody_nb100-93329
anti-GAPDH (6C5)(sc-32233, SantaCruz):mouse monoclonal antibody to GAPDH of mouse, rat, human, rabbit and Xenopus origin suitable for WB, IP and IF. https://www.scbt.com/p/gapdh-antibody-6c5
goat anti-rabbit IgG (H+L) secondary antibody (65-6120, Invitrogen): Goat polyclonal antibody to Rabbit IgG suitable for WB, IHC, ELISA and IP. https://www.thermofisher.com/antibody/product/Goat-anti-Rabbit-IgG-H-L-Secondary-Antibody-Polyclonal/65-6120
goat anti-mouse IgG (H+L) secondary antibody conjugated (65-6520, Invitrogen): Goat polyclonal antibody to mouse IgG suitable for WB, IHC and ELISA. https://www.thermofisher.com/antibody/product/Goat-anti-Mouse-IgG-H-L-Secondary-Antibody-Polyclonal/62-6520
Anti-SC35 antibody [SC-35] (ab11826, abcam): mouse monoclonal to SC35 suitable for ICC/IF. https://www.abcam.com/sc35-antibody-sc-35-nuclear-speckle-marker-ab11826.html
Anti-SON Antibody (HPA031755, Atlas antibodies):Rabbit polyclonal antibody against Human SON suitable for WB, Immunofluorescence in Cell Lines (ICC/IF). https://www.atlasantibodies.com/products/antibodies/primary-antibodies/triple-a-polyclonals/son-antibody-hpa031755/
Goat anti-rabbit-AlexaFluor 405 (A-31556, ThermoFIsher): Goat polyclonal antibody conjugated with Alexa Fluor™ 405 against Rabbit IgG(H+L) suitable for (ICC/IF). https://www.thermofisher.com/antibody/product/Goat-anti-Rabbit-IgG-H-L-Cross-Adsorbed-Secondary-Antibody-Polyclonal/A-31556

Eukaryotic cell lines

Policy information about cell lines and Sex and Gender in Research

Cell line source(s)	HEK293T (ATCC, CRL-3216<br>Phoenix-AMPHO (ATCC, CRL-3213)<br>HeLa (ATCC, CCL-2)<br>TZM-bl (NIH AIDS Reagent Program, 8129)<br>CPSF6 knockout HEK293T (CKO) (Sowd, et al, PNAS, 2016; Li, et al, mbio, 2020)
Authentication	HEK293T, Hela and TZM-bl were authenticated by the ATCC STR profiling. Phoenix-ampho and CPSF6 knockout HEK293T cells were not authenticated.
Mycoplasma contamination	Cell lines were tested monthly for Mycoplasma contamination and no contamination was found
Commonly misidentified lines<br>(See ICLAC register)	No commonly misidentified cell lines were used in the study
