# Supplementary material for: Prion-like low complexity regions enable avid virus-host interactions during HIV-1 infection
Source: Nat Commun. 2022 Oct 6;13:5879. doi: 10.1038/s41467-022-33662-6 (PMC9537594; doi:10.1038/s41467-022-33662-6)
Supplement: Supplementary file 3 — Peer Review [file 41467_2022_33662_MOESM3_ESM.pdf]

nature portfolio

Peer Review File

Prion-like low complexity regions enable avid virus-host interactions during HIV-1 infection

Open Access This file is licensed under a Creative Commons Attribution 4.0 International License, which permits use, sharing, adaptation, distribution and reproduction in any medium or format, as long as you give appropriate credit to the original author(s) and the source, provide a link to the Creative Commons license, and indicate if changes were made. In the cases where the authors are anonymous, such as is the case for the reports of anonymous peer reviewers, author attribution should be to 'Anonymous Referee' followed by a clear attribution to the source work. The images or other third party material in this file are included in the article's Creative Commons license, unless indicated otherwise in a credit line to the material. If material is not included in the article's Creative Commons license and your intended use is not permitted by statutory regulation or exceeds the permitted use, you will need to obtain permission directly from the copyright holder. To view a copy of this license, visit http://creativecommons.org/licenses/by/4.0/.

REVIEWER COMMENTS

Reviewer #1 (Remarks to the Author):

This is a clearly written manuscript describing a careful study revealing the importance of prion-like low complexity regions in binding of CPSF6 to the HIV-1 capsid lattice. This novel finding significantly extends understanding of the molecular details of a virus-host interaction whose exact function remains incompletely defined. New insights on LEN suggests that its mechanism of action may not involve competitive inhibition of CPSF6 binding. The data and analysis are of high-quality; biochemical and structural data are complemented with virology, and there is impressive correspondence in the results supporting the conclusions.

Specific comments:

While the data seem to support simultaneous binding of LEN and CPSF6, the authors do not show any data suggesting that this is generalizable to all FG-motif containing host factors. Please add a sentence or two to clarify this in the text.

Use of “C6” as acronym for CPSF6 is confusing

The phrase “failed to impact HIV-1 integration targeting preferences” can be read to mean that the CPSF6/AD and CPSF6/NE chimeras supports HIV-1’s native preference for gene-dense regions and SPADs; please re-phrase

The cryo-EM map of GST-CPSF6(LCR-FG-LCR) bound to IP6-stabilized CA tubes was detailed enough to allow modeling of the

N-terminal LCR and the FG peptide of the CPSF6269-327 fragment: At 7.4 Å resolution, this could be misleading to non-structural readers. Please explain more clearly what is meant by “detailed enough”. Some brief explanation of how the modeling was done should be included in this section as well (with details in the methods).

GST effects – the lack of contribution from the tag is better inferred from comparing GST-CPSF6(LCR-FG-LCR) and GST-CPSF6/AD and NE chimeras with non-LCR regions.

In contrast from X-ray crystal structures, which show six CPSF6 FG peptides bound to six hydrophobic pockets in an isolated CA hexamer: This is incorrect. Both Price et al and Bhattacharya et al structures showed that not all of the six binding sites in the P212121 hexamer are occupied by the FG peptide.

TRIM5α-TRIM5α assemblies are mediated by both coiled-coil interactions and B-box 2 domain interactions, not just the coiled-coil

Lack of line numbers made reviewing onerous.

Reviewer #2 (Remarks to the Author):

Wei et al apply a combination of structural, biochemical and virological assays to characterize interactions between the HIV-1 capsid and cellular binding partners CPSF6, NUP153 and SEC24C. This is an interesting and important question. They conclude that interactions between low complexity regions within the host cell proteins are essential for binding and act by increasing the avidity of interaction with the capsid. They further conclude that the capsid binding drug lenacapavir binds to unoccupied binding pockets on capsid without the need to displace bound host proteins. In my opinion, the data convince that the properties of the regions flanking the FG peptide contribute to binding of CPSF6 to HIV-1 capsid lattices both in vitro and in cells. In my view the data are not strong enough to support the proposed structural mechanism.

I have the following major concerns.

1. A control should be included to show that flanking sequences containing typical levels of charged residues do not interfere with binding of the FG repeat region to the pocket in capsid. For example, LCR-FG-LCR and the sequences where LCR is replaced with “typical resides” should bind with similar affinity to isolated capsid hexamers where there is no avidity contribution, albeit with low uM-mM affinities.

2. The cryoEM experiments are performed with CA and CPSF6 concentrations of above 100uM. To understand expected behaviour in this concentration range it would be helpful to include a control titration with the different CPSF6 constructs, perhaps similar to that shown for one such construct in Extended figure 8. Why do the control constructs not bind the tubes at this high concentration given the

presence of the FG sequence? If a peptide is added at this concentration, does it bind all six sites on the hexamer?

3. My main concerns relate to the cryoEM work:

- The authors claim that the structure allows modelling of the FG repeat – at the measured stoichiometry the FG repeat should be clearly visible within its binding pocket in two of six CA molecules in the cryoEM reconstruction at similar density to CA, but I cannot see it and would not be able to model it. Why not? If it is clearly visible, please include a figure clearly showing the FG repeat. This can be further validating my making sure that it matches the X-ray structure filtered to the same resolution.

-The resolution of the cryoEM structure is not expected to be the same at all radii. The weak additional density at high radius is presumably resolved at lower resolution than the strong capsid density. A plot of resolution at different radii within the helical reconstruction should be provided (for an example for TMV, see Fig 5G in Fromm et al, J. Struct Biol 2015). The high radius features should be filtered to the measured radial or local resolution. What is the resolution of which LCR and GST regions are resolved? They should be filtered to this measured resolution prior to interpretation. The measured resolution will likely be substantially lower than that of the capsid domains, I am very skeptical that it can be detailed enough to allow modelling of the LCR.

- The authors must carefully rule out the possibility that the density interpreted as the LCR is helically symmetrized noise. CA tubes adopt mixtures of different helical parameters but only one structure is presented here for each construct. It should also be possible to reconstruct other tube families from the same dataset with different helical parameters. If the authors interpretation is correct, the LCR density should be the same in all reconstructions. Repeating the data collection and reconstruction with a redetermination of helical symmetry would also be a useful validation.

- If the above validations can be performed, it is then also necessary to validate that the model interpreted from the density is correct. To me the link between Fig 4 d and e is tenuous – what other possible arrangements of the protein could be accommodated in the density?

Reviewer #3 (Remarks to the Author):

The manuscript entitled, 'Prion-like low complexity regions enable avid virus-host interactions during HIV=-1 infection,' by Wei et al examines the structural basis of interactions between CPSF6 and the HIV capsid. This is of significance as interactions between capsid and cellular proteins such as CPSF6, NUP153 and SEC24c. All of the proteins are similar in that they contain a central phenylalanine/glycine rich domain that has been described to interact with the capsid. These interactions are critical to the viral life cycle as the cellular proteins are involved in shuttling of the capsid through the cytoplasm and into the nucleus. The authors set out to describe the interactions with these proteins (with a focus on CPSF6) with the complete and organized capsid to identify why the previous interaction of the FG regions was at relatively low affinity. They accomplish this through a series of biochemical, virologic and structural studies that define the importance of prion-like low complexity domains that surround the FG region. These LCR are of interest as they are uncharged regions of amino acids that can take on the shape necessary to interact with a target molecule, thus driving a templated interaction. Through application of their various technique and the evaluation of mutant CPSF6 constructs the authors show convincing evidence that LCRs are critical to CPSF6 functional interaction with capsid - particular the N-terminal LCR. This manuscript expands our understanding of how CSPF6 interacts with the capsid lattice and likely gives us clues to similar interactions with the nuclear pore. Analysis of the effect of the novel antiviral LEN reveal possible mechanism of LEN activity (stabilization of the CORE). Additionally, the nature of CPSF6 binding (to areas between hexamers) lends support to the idea that HIV cores present in the nucleus are still structurally intact. It is well written and adds to the field.

I do have the following concerns:

- Each of the critical initial pieces of data (Table 1, Fig 1A-C, and FIg 1D-F) are all performed using different substrates and different assays. The table shows binding affinity with hexamers (that are shown in 1G to have weaker binding in general), panels A-C use nanotubes and D-F use intact HIV cores. It is impossible to make necessary comparisons between these (ie is LCR needed compared to FG alone). Although mostly covered in later assays this makes the critical first figure a little weak.

- Fig 1A-C - There appears to be a size shift in CPSF6 in the bound form. Is this seen consistently? If real, this could indicate a modification that is associated with binding (given this is lysate a possibility) - do the structural studies suggest unmodified CPSF6? Additionally, the nonspecific band ~55 kDA in panel B appears to be depleted by the presence of CA, but is not found in the CA bound fraction. WHat is this band and where did it go?

- In general, it would be best to have the technique used to generate the data specifically named in the figure legend or the text. The methods section is complete and I could find everything, but it was often hard to determine which assay was actually used (ie 1D-F,)

- Figure 2 - The PLA assay shows convincing co-localization of CSPF6 and CA in the nucleus. CPSF6 is controlled for in the supplementary materials. CA is not. Subsequent data (Fig 3) suggest an accumulation of CA at the periphery of the nucleus. This could be indicative of a deficit in entry through the nuclear pore. Although the overall findings suggest a difference in interaction, the data presented here could be a loss of localization due to a decreased presence of CA in the nucleus. This is an important distinction as it would help to identify the effect of interactions in the cytoplasm versus the nucleus. Comparisons used for the t-test should be explicitly stated.

- Figure 3 - The microscopy suggests the accumulation of the PIC at the periphery of the nucleus. This could be improper positioning after transit through the pore, as would be supported by the increase in LAD associated integrants, but some of the foci appear to be outside of the nucleus. Where are these complexes located? Does CPSF6 alteration change the rate of nuclear import? A time course to establish overall kinetics of movement into the nucleus and association with nuclear speckles would address this and further define the importance of CPSF6 in the process. This becomes more critical in light of later discussions regarding the stability of the core in the nucleus (and how it is affected by LEN). A time course would establish that the process has been slowed as opposed to completed early.

- Figure 3D-F - How many total integrants were sequenced per sample per cell? The change in localization in 3A suggests that there may be less integration overall. Is 3E a percentage of integrants near a gene dense region? or the average number of genes within 1Mb?

- Extended data 3B - Co-localization should be quantified. C6/NE appears to be showing the same pattern as the LCR containing mutants.

- Figure 5 - Panel A suggests that association with the core still occurs without the critical anchoring domain. This seems contradictory to much of the findings.

- Figure 6 - This is an excellent set of experiments. It might be helpful to have a schematic similar to 6A placed earlier in the manuscript so that the reader can visually examine the various LCRs used.

- Figure 7 - What are the baseline differences in infection of each of the different cell types?

- The authors conclude that LEN either displaces CPSF6 or can interact with the CPSF6/CA complex. The final explanation given would seem to be a third option: that LEN interacts with a different area of the CA not occupied by CPSF6.

- Do your structural models allow you to predict how many hexamers on the core (not a nanotube) are occupied by CPSF6? Do you think the irregular shape of the capsid influences this?

- The conclusion that CPSF6:CPSF6 interactions are templated by binding of the FG to CA is likely true, but not conclusively proven by this data alone.

Response to Referees Letter

We have found the reviewers' comments constructive and revised the manuscript accordingly. Please note the following.

i) The revised text is in red.

ii) New results are included in Figures 1b and c, Figures 5d-h; Extended Data Fig 4b, Extended Data Figure 6, Extended Data Figure 7c, Extended Data Figures 8-11, Extended Data Figure 13, and Supplemental Table 3.

iii) For clarity and to include protein sequences where needed, as requested by reviewer 3, we have reorganized previously presented data by expanding total numbers of main and extended data figures.

iv) We have included three additional authors: Arpa Hudai, Gregory A. Voth and Lorenzo Briganti. Arpa Hudai and Gregory A. Voth performed all atom molecular dynamic simulations (new Extended Data Fig. 13) and Lorenzo Briganti performed AlphaFold structural predications (new Extended Data Fig. 4b).

Below is our point-by-point response to reviewers' comments.

REVIEWER #1.

Comment: *While the data seem to support simultaneous binding of LEN and CPSF6, the authors do not show any data suggesting that this is generalizable to all FG-motif containing host factors. Please add a sentence or two to clarify this in the text.*
Response: We have added the following sentence (page 18): "Follow up studies are warranted to characterize the interplay between LEN and other FG motif containing proteins such as NUP153 and SEC24C with HIV-1 cores to elucidate additional, albeit less potent antiviral activities of this multimodal inhibitor during nuclear import and cytoplasmic trafficking."

Comment: *Use of "C6" as acronym for CPSF6 is confusing*
Response: We have changed "C6" to "CPSF6" throughout the text and figures.

Comment: *The phrase "failed to impact HIV-1 integration targeting preferences" can be read to mean that the CPSF6/AD and CPSF6/NE chimeras supports HIV-1's native preference for gene-dense regions and SPADs; please re-phrase*
Response: We have rephrased this sentence to read (page 7): "Expression of the chimeric proteins CPSF6/AD and CPSF6/NE, which lacked the LCR context for the FG peptide, exhibited HIV-1 integration patterns similar to those observed in CKO cells".

Comment:: *The cryo-EM map of GST-CPSF6(LCR-FG-LCR) bound to IP6-stabilized CA tubes was detailed enough to allow modeling of the N-terminal LCR and the FG peptide of the CPSF6269-327 fragment: At 7.4 Å resolution, this could be misleading to non-structural readers. Please explain more clearly what is meant by "detailed enough". Some brief explanation of how the modeling was done should be included in this section as well (with details in the methods).*
Response: Please see our detailed responses to Reviewer 2, which covers this comment.

Comment: *GST effects – the lack of contribution from the tag is better inferred from comparing GST-CPSF6(LCR-FG-LCR) and GST-CPSF6/AD and NE chimeras with non-LCR regions.*
Response: We agree. This is further supported by new experimental data in Extended Data Fig 8 (also see our responses to reviewer 2).

Comment: *In contrast from X-ray crystal structures, which show six CPSF6 FG peptides bound to six hydrophobic pockets in an isolated CA hexamer: This is incorrect. Both Price et al and Bhattacharya et al structures showed that not all of the six binding sites in the P212121 hexamer are occupied by the FG peptide.*
Response: Thank you for bringing this to our attention. In the Extended Data Figure 12 legend we compare our CA_{hex} + IP6 + CPSF6 structure with published structures of CA_{hex} + IP6 and CA_{hex} + CPSF6 FG peptide, and we

find an excellent agreement between our tripartite complex versus the published bipartite complexes. All deposited crystal structures of CPSF6 FG peptide + CA_{hex} complexes including PDB: 4U0A (P6 space group), PDB: 4U0B (P212121 space group), PDB: 4WYM and PDB: 6AY9 show 6 CPSF6 FG peptides bound to a CA_{hex}. However, the reviewer may have more direct knowledge of broader, unpublished crystallographic efforts. If this assumption is correct, sub-stoichiometric binding of the CPSF6 FG peptide to a CA_{hex} would not be surprising given very low binding affinity for these interactions. We also note that PDB: 4U0C (P6 space group) and PDB: 4U0D (P212121 space group) show 6 and 3 NUP153 FG peptides bound to a CA_{hex}. Again, low affinity binding of FG peptides could be the primary reason for sub-stoichiometric interactions of the NUP153 FG peptide + CA_{hex} observed in certain crystal forms. To avoid any confusion with respect to the particular sentence cited above by the reviewer, we removed the reference to the previous studies and instead, we now compare our X-ray and cryo-EM results (see page 15): "In contrast to our X-ray crystal structure (Extended Data Fig 12a), which shows six CPSF6 FG peptides bound to six hydrophobic pockets in an isolated CA hexamer, our cryo-EM studies reveal the differential stoichiometry of CPSF6(LCR-FG-LCR) molecules binding to only two out of six cognate sites in the context of extended CA lattices (Fig 5g)."

Comment: *TRIM5α-TRIM5α assemblies are mediated by both coiled-coil interactions and B-box 2 domain interactions, not just the coiled-coil*
Response: Agreed and corrected. The revised sentence on page 17 reads: "TRIM5α-TRIM5α assemblies are mediated by B-box 2 and coiled-coil interactions resulting in a TRIM5α hexagonal cage surrounding the hexameric CA lattices"

Comment: *Lack of line numbers made reviewing onerous.*
Response: We apologize. Line numbers are now included.

REVIEWER #2.

Comment: *A control should be included to show that flanking sequences containing typical levels of charged residues do not interfere with binding of the FG repeat region to the pocket in capsid. For example, LCR-FG-LCR and the sequences where LCR is replaced with "typical resides" should bind with similar affinity to isolated capsid hexamers where there is no avidity contribution, albeit with low uM-mM affinities.*
Response: This is an excellent point. See new **Extended Data Fig 8** and the respective figure legend, which conclusively address the raised question. Specifically, Extended Data Fig 8b shows similarly low binding affinities of GST-CPSF6 and GST-CPSF6(FG)/nonLCR to isolated CA hexamers. Also, note that GST-CPSF6(FG)/nonLCR exhibits similarly low binding affinity to native cores and isolated hexamers (compare **a** and **b** in **Extended Data Fig 8**). In sharp contrast, GST-CPSF6(LCR-FG-LCR) exhibits markedly enhanced binding affinity with respect to native cores vs isolated hexamers (compare **a** and **b** in **Extended Data Fig 8**). In addition to these experimental results, we performed AlphaFold structural predictions of the chimeric protein segments investigated here. Please see **Extended Data Fig 4b**, the respective figure legend as well as the relevant text in Results on page 6.

Comment: *The cryoEM experiments are performed with CA and CPSF6 concentrations of above 100uM. To understand expected behaviour in this concentration range it would be helpful to include a control titration with the different CPSF6 constructs, perhaps similar to that shown for one such construct in Extended figure 8. Why do the control constructs not bind the tubes at this high concentration given the presence of the FG sequence? If a peptide is added at this concentration, does it bind all six sites on the hexamer?*
Response: Please see new **Extended Data Fig 8**, which shows that GST-CPSF6(FG)/nonLCR binds to native cores, isolated CA hexamers and CA nanotubes, albeit GST-CPSF6(FG)/nonLCR displays similarly low binding affinity to all these CA assemblies. This binding is FG mediated as evidenced by **Extended Data Fig 8c**, which shows that GST-CPSF6(ΔFG)/non-LCR fails to bind to CA nanotubes. Taken together, our biochemical observation that only residual binding of 120 µM GST-CPSF6(FG)/nonLCR to CA nanotubes is seen in **Extended Data Fig 8c** explains why we do not see its density in cryo-EM maps.

Comment: *- The authors claim that the structure allows modelling of the FG repeat – at the measured stoichiometry the FG repeat should be clearly visible within its binding pocket in two of six CA molecules in the cryoEM reconstruction at similar density to CA, but I cannot see it and would not be able to model it. Why not? If it is clearly visible, please include a figure clearly showing the FG repeat. This can be further validating my making sure that it matches the X-ray structure filtered to the same resolution.*

Response: Images of our CPSF6-decorated CA tubes show that CPSF6 binding is not uniform, but instead seems to happen in patches. This is consistent with what is seen for many (if not most) proteins that decorate helical tubes. The combined effect of this substoichiometric CPSF6 binding and the limited resolution of our cryo-EM helical maps makes FG peptide density hard to detect. We used a pseudo-single particle approach to analyze our data and, in the resulting map, we can see an indication of FG peptide density in the expected positions. However, due to the substoichiometric CPSF6 binding, FG peptide density is weaker than CA density and is apparent only at relatively low thresholds. We felt that the quality of that pseudo-single particle map was not sufficiently high to merit inclusion in the manuscript. In contrast, displaying the symmetrized helical map at an appropriate threshold shows clearly how CPSF6 density contacts CA hexamers and this is shown in the new Fig. 5g. We know from results of published x-ray crystallography, biochemistry and virology studies that the FG peptide directly engages with the CA hydrophobic pocket. In agreement with these well-established results (which are confirmed by our own x-ray crystallography, biochemistry and virology results presented in our manuscript), our cryo-EM studies show that CPSF6 density extends toward specific CA hexamer hydrophobic pockets, whereas the map calculated from GST-CPSF6 (ΔFG) does not show any additional density around CA hexamers. Furthermore, our cryo-EM observation that CPSF6 makes two contacts on neighboring CA hexamers is consistent with our biochemical data indicating a 2:6 CPSF6:CA interaction stoichiometry. We think that the convergence of several independent lines of experimental evidence strongly supports the conclusion that the GST-CPSF6 binding to CA tubes that we see in our cryo-EM maps is due to direct interactions of CPSF6 FG peptide to cognate CA hydrophobic pockets. The structural details of FG motif binding to CA hexamers have been well-established. The principal contribution from our study is uncovering the mechanism for avid binding of CPSF6 (and likely SEC24C and NUP153) to a mature capsid lattice, and our cryo-EM map provides critical information by demonstrating multivalent CPSF6 LCR assembly along adjoining capsid hexamers.

For context, published cryo-EM maps of TRIM-5α bound to CA tubes have a comparably limited resolution. Yet, large hexagonal lattices of polyvalent TRIM-5α assemblies on top of the mature capsid lattice can be clearly seen. Those maps could not delineate how the short TRIM-5α peptide (the SPRY-motif) binds to CA hexamers and, since there is no high-resolution crystal structure of the SPRY-motif bound to CA hexamers, the structural details of TRIM-5α binding to CA remain unknown. Yet, cryo-EM studies of CA-TRIM-5α interaction have been instrumental for understanding the mechanism (validated by virology assays) behind avid binding of TRIM-5α to mature capsid.

Comment: *-The resolution of the cryoEM structure is not expected to be the same at all radii. The weak additional density at high radius is presumably resolved at lower resolution than the strong capsid density. A plot of resolution at different radii within the helical reconstruction should be provided (for an example for TMV, see Fig 5G in Fromm et al, J. Struct Biol 2015). The high radius features should be filtered to the measured radial or local resolution. What is the resolution of which LCR and GST regions are resolved? They should be filtered to this measured resolution prior to interpretation.*

Response: We thank the reviewer for making this excellent point. In the original version of the manuscript we low-pass filtered our cryo-EM maps to facilitate interpretation of poorly ordered density, but the reviewer's suggestion to filter the maps according to local resolution is clearly a better approach than applying a uniform low-pass filter. We have calculated local resolution values and filtered the maps accordingly (see **Extended Data 9**).

Comment: *The measured resolution will likely be substantially lower than that of the capsid domains, I am very skeptical that it can be detailed enough to allow modelling of the LCR.*

Response: We apologize for this unintentionally misleading general statement, and for not explaining the purpose and outcomes of our initial modeling efforts. The reviewer is absolutely correct that our cryo-EM results are not at the resolution needed to provide specific details for LCR conformations. Instead, our initial efforts have focused on making sure that our interpretation of GST-CPSF6 density was correct. We have made the following observations:

1) The available X-ray structure of GST fits well within the respective cryo-EM density;

2) The N-terminal LCR chain could be readily placed within the CPSF6 density. Furthermore, N-terminal LCR accounted for the bulk of CPSF6 density;
3) The length of the N-terminal LCR allowed it to readily extend to the CPSF6:CA contact points (new Fig 5g) thereby enabling the FG peptide binding to cognate hydrophobic CA pockets as seen by X-ray crystallography.
4) The close proximity of LCR chains from different CPSF6 molecules have suggested extensive LCR-LCR interactions.

Collectively, these spatial constraints together with corroborating findings from HDX experiments (protections in LCRs), biochemical assays (2:6 CPSF6:CA binding stoichiometry), and extensive virology assays (indicating the biological significance of CPSF6 LCRs and more specifically of the N-terminal LCR), allowed us to propose the LCR-LCR mediated mechanism for polyvalent CPSF6 assembly onto mature CA lattice. However, as we have acknowledged above limited resolution of cryo-EM maps did not allow us to delineate different LCR conformations or specific details of LCR-LCR interactions. To make this absolutely clear, we now offer a more conservative, diagrammatic representation of CPSF6-CPSF6 interactions (see **Extended Data Fig 11b**) that is consistent with the limited resolution of our cryo-EM maps. Furthermore, we have accordingly revised the cryo-EM sections of the text on pages 8 and 9.

Importantly, we have now extended our efforts to perform all atom (AA) molecular dynamic (MD) simulation (see **Extended Data Fig 13** and respective section titled (see pages 10 and 11): "Conformational analysis of GST-CPSF6$_{261\text{-}358}$ (LCR-FG-LCR) bound to CA-hexamer lattice from all-atom (AA) molecular dynamics (MD) simulations". The main findings of our AA MD simulations are: i) The N-terminal LCRs primarily contribute to the CPSF6-CPSF6 assembly forming a network of highly interacting CPSF6 chains templated by the CA lattice; ii) N-terminal LCRs adopt multiple conformations, which allow effective assembly of the highly interactive network of CPSF6 chains.

Also note that the AA MD simulation results regrading conformational flexibility of LCR-LCR interactions are consistent with our experimental results showing that native CPSF6 LCR can effectively be replaced by other prion-like LCRs with completely different primary structures from unrelated proteins. These findings are consistent with what is generally known about prion-like LCR-LCR interactions. When prion-like LCRs are brought in close proximity (in the case with CPSF6 by its FG motif binding to adjacently positioned CA hexamers), they form highly interactive network of hydrophobic chains, which typically adopt multiple conformations. Taken together, our AA MD simulations further support of our extensive virology, biochemistry, cryo-EM and HDX-MS results, which collectively reveal the previously undescribed mechanism of prion-like LCR mediated high affinity binding of CPSF6 to CA lattices.

Comment: *- The authors must carefully rule out the possibility that the density interpreted as the LCR is helically symmetrized noise. CA tubes adopt mixtures of different helical parameters but only one structure is presented here for each construct. It should also be possible to reconstruct other tube families from the same dataset with different helical parameters. If the authors interpretation is correct, the LCR density should be the same in all reconstructions. Repeating the data collection and reconstruction with a redetermination of helical symmetry would also be a useful validation.*

Response: Another excellent point. We have been well-aware of the risk of misinterpreting artifactual density resulting from unidentified problems in helical processing and had calculated a second CPSF6-CA map from a different subset of helical images to verify that what we considered CPSF6-related density was real. As the reviewer suggested, we now include **Extended Data Fig 11a** showing the excellent correspondence (accounting for differences in helical symmetry) of CPSF6 density in both, independently calculated helical maps.

Comment: *- If the above validations can be performed, it is then also necessary to validate that the model interpreted from the density is correct. To me the link between Fig 4 d and e is tenuous – what other possible arrangements of the protein could be accommodated in the density?*

Response: Also a very good point. Although its resolution is limited, the cryo-EM map of CPSF6-CA tubes uniquely determines the "topology" of CPSF6 interaction with the CA hexamer lattice by identifying the position of GST tags, the points of LCR interaction with the CA lattice and the position of LCR-related density. This structural information, along with the length of LCR domains and information from complementary biochemical and HDX results, was considered to put forward the diagrammatic representation of LCR interactions presented in a revised **Extended Data Fig. 11b**.

In conclusion, we would like to make the following general point. Structural studies with disordered FG containing prion-like LCRs are notoriously difficult. Therefore, it is remarkable that we've been able to account for the CPSF6 LCR density in our Cryo-EM maps and detect protection in LCR by HDX-MS. Despite limited resolution of our cryo-EM maps (as expected from conformationally flexible LCR-LCR interactions), our principal structural findings -- i) CPSF6 molecules contact two adjoining CA hexamers (**Fig 5g**), and ii) LCR-LCR interactions populate zig-zagging density extending between the rows of adjoining hexamers (**Fig 5h**) -- strongly support our main discovery of the previously undescribed mechanism of prion-like LCR mediated, avid, polyvalent binding of FG motif containing CPSF6 (and likely also NUP153 and SEC24C) to mature CA lattices.

REVIEWER #3.

Comment: *- Each of the critical initial pieces of data (Table 1, Fig 1A-C, and FIg 1D-F) are all performed using different substrates and different assays. The table shows binding affinity with hexamers (that are shown in 1G to have weaker binding in general), panels A-C use nanotubes and D-F use intact HIV cores. It is impossible to make necessary comparisons between these (ie is LCR needed compared to FG alone). Although mostly covered in later assays this makes the critical first figure a little weak.*
Response: We have now included new **Extended Data Fig 8**, which further strengthens initial data presented in **Fig 1**.

Comment:*- Fig 1A-C - There appears to be a size shift in CPSF6 in the bound form. Is this seen consistently? If real, this could indicate a modification that is associated with binding (given this is lysate a possibility) - do the structural studies suggest unmodified CPSF6?*
Response: The apparent differences in migration patterns in the previous image that were correctly noticed by the reviewer were due to different NaCl content in tested samples: cell lysates and pulled-down fractions contained low and very high NaCl, respectively. Now we have adjusted NaCl content to be very similar in all fractions, which revealed very similar migration patterns for CPSF6 in cell lysates and pull-down fractions (see New **Figure 1a**).

Comment: *Additionally, the nonspecific band ~55 kDA in panel B appears to be depleted by the presence of CA, but is not found in the CA bound fraction. WHat is this band and where did it go?*
Response: We have now used a more specific antibody (NB100-93329, Novus) against NUP153 and we no longer see the non-specific band observed previously (see new **Figure 1b**). Furthermore, we added the following statement to the Figure 1 legend: "The proteins of interest were visualized by antibodies ab175237(Abcam) against CPSF6, NB100-93329 (Novus) against NUP153, and ab122633 (Abcam) against SEC24C".

Comment:*- In general, it would be best to have the technique used to generate the data specifically named in the figure legend or the text. The methods section is complete and I could find everything, but it was often hard to determine which assay was actually used (ie 1D-F,).*
Response: We now state in Figure 1 legend: "(d-f) Quantitation of GST-mediated affinity pull-down of native HIV-1 cores bound to indicated concentrations of GST-CPSF6$_{261-358}$(LCR-FG-LCR) vs GST-CPSF6(FG)/nonLCR…"

Comment: *- Figure 2 - The PLA assay shows convincing co-localization of CSPF6 and CA in the nucleus. CPSF6 is controlled for in the supplementary materials. CA is not. Subsequent data (Fig 3) suggest an accumulation of CA at the periphery of the nucleus. This could be indicative of a deficit in entry through the nuclear pore. Although the overall findings suggest a difference in interaction, the data presented here could be a loss of localization due to a decreased presence of CA in the nucleus. This is an important distinction as it would help to identify the effect of interactions in the cytoplasm versus the nucleus.*
Response: The reviewer is correct that PLA assays reveal only interacting protein-protein complexes whereas localization of non-interacting individual proteins could not be determined by this assay. Hence, we carried out complementary assays depicted in **Figure 3** (also see our response with respect to Figure 3 below). As to which specific cellular compartments CPSF6 interacts with HIV cores, we note that in Figures 2 and 3 we examined interactions between predominantly nuclear full-length CPSF6 and HIV-1, whereas in Figures 7 and 8 we analyzed

interactions between cytoplasmic $CPSF6_{358}$ and incoming HIV-1. All these assays demonstrate an essential role of the CPSF6 LCR for interactions with incoming HIV-1 cores during infection.

Comment: *Comparisons used for the t-test should be explicitly stated.*
Response: We now state in Figure 2 legend: "Statistical significance of comparison of WT versus the chimeric CPSF6 proteins was determined by Student t-test."

Comment: *- Figure 3 - The microscopy suggests the accumulation of the PIC at the periphery of the nucleus. This could be improper positioning after transit through the pore, as would be supported by the increase in LAD associated integrants, but some of the foci appear to be outside of the nucleus. Where are these complexes located? Does CPSF6 alteration change the rate of nuclear import? A time course to establish overall kinetics of movement into the nucleus and association with nuclear speckles would address this and further define the importance of CPSF6 in the process. This becomes more critical in light of later discussions regarding the stability of the core in the nucleus (and how it is affected by LEN). A time course would establish that the process has been slowed as opposed to completed early.*
Response: This is an excellent point and we present new Extended Data Fig 6 which shows the kinetics of nuclear import of HIV-1 VRCs in infected cells. We have also revised the text with regard to the previous and this comment (see the first two paragraphs in the section "The LCR is required for CPSF6's function in HIV-1 infection." Pages 6-7). We conclude that nonLCR containing chimeric CPSF6/AD and CPSF6/NE proteins fail to engage with HIV-1 during infection in general, whereas LCR containing full-length CPSF6/FU and CPSF6/CD can effectively substitute WT CPSF6 during HIV-1 nuclear import and transport to NSs.

Comment: *- Figure 3D-F - How many total integrants were sequenced per sample per cell? The change in localization in 3A suggests that there may be less integration overall.*
Response: Thank you for this question. As 5×10^4 cells were infected per sample and all infections were performed in duplicate (save for the CKO sample, where the data was from a prior study), the number of total integrants sequenced per sample per cell varied from approximately 0.05 to 0.65.

As highlighted in the "Sequencing of HIV-1 integration sites" section of Methods in the paper's Supplement, we have used LM-PCR to generate DNA libraries for integration site sequencing. This process follows numerous steps, including: genomic DNA shearing > DNA purification > asymmetric linker ligation > DNA purification > PCR (multiplexed x16) > PCR pooling and purification > 2nd round PCR (multiplexed x16) > PCR pooling and purification > Illumina sequencing (Matreyek et al., 2014 Retrovirology; Serrao et al., 2016 J Vis Exp).

Due to the multi-step library preparation process, the number of sites recovered per sample need not reflect the level of HIV-1 infection across samples, and indeed, integration site recovery varied in this study by as much as ~13-fold across samples. Although CPSF6 plays a critical role in HIV-1 integration targeting in all cell types, we would not expect the different HEK293T cell samples used in this experiment to display large infection differences (Sowd et al., 2016 PNAS; Francis et al., 2020 Nat Commun; Li et al., 2020 mBio). To address the comment about integration levels between samples, we now provide overall infection levels of the various cell samples, which, predictably, did not vary from one another in any meaningful way (**Extended Data Figure 6a**). We accordingly conclude that the variation in site recovery across samples is due to the nature of the library prep, which requires multiple steps of DNA purification and rounds of PCR amplification. Due to the nature of the statistical tests employed for data analysis (Fisher's exact; Wilcoxon rank sum), we were able to derive meaningful differences between the patterns of HIV-1 integration in the difference cell samples despite the variation in integration site recovery across the samples (**Supplementary Table 2**).

Comment: *Is 3E a percentage of integrants near a gene dense region? or the average number of genes within 1Mb?*
Response: Thank you for this comment. The new **Fig 4b** y-axis label was relabeled "Avg. gene number/Mb"

Comment: *- Extended data 3B - Co-localization should be quantified. C6/NE appears to be showing the same pattern as the LCR containing mutants.*
Response: Thank you for this excellent suggestion. See the quantitation results in new **Extended Data Fig 7c**.

Comment: *- Figure 5 - Panel A suggests that association with the core still occurs without the critical anchoring domain. This seems contradictory to much of the findings.*
Response: In fact, HDX-MS results show the opposite. It seems that we failed to explain the technical details regarding how HDX-MS data are generated and analyzed. The assays are performed with GST-CPSF6$_{261-358}$ +/- CA and GST-CPSF6$_{261-358}$(ΔFG) +/- CA. The protections are only observed in the context of GST-CPSF6$_{261-358}$ + CA but not GST-CPSF6$_{261-358}$(ΔFG) + CA, which indicate that the anchoring FG peptide is essential for the complex formation.

With respect to technical aspects of HDX-MS experiments. These experiments are performed with full-length GST-CPSF6$_{261-358}$ +/- CA and GST-CPSF6$_{261-358}$(ΔFG) +/- CA. The reactions are then quenched (see Materials and Methods), and the proteins are digested by pepsin to generate individual short peptides amenable for MS/MS analysis. The protection patterns of resulting fragments tell us which segments in the context of the full-length protein were protected upon the complex formation. We understand that reviewer's question is related to protection of the CPSF6 (aa266-315) peptide. What we've been trying to explain is that since this peptide does not contain the FG motif, its protection in the context of full-length GST-CPSF$_{261-358}$, was due likely to LCR-LCR interactions, which is consistent with all other results in our manuscript. Again, the aa266-315 peptide is specifically protected in the context of GST-CPSF6$_{261-358}$ + CA but not with GST-CPSF6$_{261-358}$(ΔFG) + CA, which argues that both the anchoring FG motif and LCR-LCR interactions are important for the complex formation.

We have now rearranged the labels in **Figure 6** and revised the respective text in Results (pages 11-12) to make above points clearer.

Comment: *- Figure 6 - This is an excellent set of experiments. It might be helpful to have a schematic similar to 6A placed earlier in the manuscript so that the reader can visually examine the various LCRs used.*
Response: Thank you for this very helpful suggestion. We now provide schematics and amino acid sequences for all our chimeric protein constructs, which are shown in respective Figures (see Extended Data Figures 2, 3, 5 and 8).

Comment: *- Figure 7 - What are the baseline differences in infection of each of the different cell types?*
Response: We have now added the following information to the Figure 9 (formally Figure 7) legend: "VSV-G pseudotyped HIV-scarlet viruses (MOI=0.5) were used to infect indicated cells. The infection levels in different cell lines without LEN treatment were 43.9 ± 2% (WT), 38.5 ± 0.9% (CKO) and 38.4 ± 1.6% (WT+CPSF6)."

Comment:*- The authors conclude that LEN either displaces CPSF6 or can interact with the CPSF6/CA complex. The final explanation given would seem to be a third option: that LEN interacts with a different area of the CA not occupied by CPSF6.*
Response: This is an excellent suggestion and we have now revised the title of this section to read (page 14): "**LEN interacts with HIV-1 cores at unoccupied sites without displacing pre-bound CPSF6**" as well as modified the respective sections of the text to read (page 14): "ii) LEN can interact with HIV-1 cores at unoccupied sites without displacing pre-bound CPSF6 and thereby inhibit infection"; (page 16) "Thus, LEN does not have to outcompete tightly bound CPSF6 from the HIV-1 cores. Instead, the inhibitor effectively engages with unoccupied hydrophobic CA sites and allosterically modulates the mature CA lattice."

Comment: *- Do your structural models allow you to predict how many hexamers on the core (not a nanotube) are occupied by CPSF6? Do you think the irregular shape of the capsid influences this?*
Response: Predicting the number of hexamers in the conical core that bind to CPSF6 would be highly speculative and we refrain from doing so. We experimentally determined the binding stoichiometry between GST-CPSF6 and tubular CA assemblies (**Extended Data Fig 16**). However, it is not feasible to extend these assays to quantitate CPSF6 interactions with native cores because of the difficulty of obtaining adequate quantities of sufficiently purified and stable native cores for such assays.

On the other hand, based on the available literature and our current findings we are willing to speculate about CPSF6 binding to CA nanotubes vs native cores. The major notable difference between these CA assemblies is the presence of pentamers in the native cores. The recent study from the Pornillos group (presented at the Retroviruses meeting at CSHL in May this year and later at the virtual HIV Structural Biology meeting) has revealed that the CPSF6 FG peptide specifically binds to native CA hexamers but not to native CA pentamers. While we cannot cite these exciting unpublished studies, they in turn reinforce the biological relevance of our

current work with curved hexameric lattices. We furthermore note that the published structural studies (ref#9 and 10) have established close structural similarities between hexameric CA lattices in the context of nanotubes vs native cores. Our new all atom molecular dynamic simulation results in **Extended Data Fig 13** indicate high conformational flexibility of LCR-LCR interactions. Taken together, it is logical to propose that our findings with GST-CPSF6 binding to nanotubes can be extended to CPSF6 interactions with native HIV-1 cores. This notion is supported by our extensive virology experiments that highlight the essential role of CPSF6 LCR for the virus-host interactions. We have now added the following text to Discussion (page 16-17): "While our cryo-EM experiments have been performed with tubular CA assemblies and GST-CPSF6$_{261-358}$, our virology experiments have validated an essential role of the LCR in the context of full-length CPSF6 interactions with HIV-1 cores in infected cells (**Figs 2-4, Extended Data Fig 7**). We also note close structural similarities between curved hexameric CA lattices in the context of tubular assemblies versus native conical capsid [9,10]. Therefore, it is logical to propose that the LCR-LCR interactions, which exhibit high conformational flexibility (**Extended Data Fig 13**), mediate avid, polyvalent assembly of CPSF6 molecules onto the conical capsid during early steps of HIV-1 replication."

Comment: - *The conclusion that CPSF6:CPSF6 interactions are templated by binding of the FG to CA is likely true, but not conclusively proven by this data alone.*

Response: We agree with the reviewer's assessment. To further clarify this point, we extended our previous statement to read (see Discussion on pages 16): "In turn, CPSF6-CPSF6 interactions are templated by binding of the embedded FG peptides to a subset of cognate hydrophobic CA pockets positioned along adjoining hexamers (**Fig 5, Extended Data Fig 11**). This notion is supported by the observations that CPSF6(ΔFG) failed to induce CPSF6-CPSF6 interactions *in vitro* in the presence of the mature CA lattice (**Fig 6** and **Extended Data Fig 14**) or inside infected cells [4]."

REVIEWERS' COMMENTS

Reviewer #2 (Remarks to the Author):

Overall, my view is that the extensive biochemical and virological data, together with the interesting and novel ideas presented, will be a valuable and interesting addition to the literature. The manuscript has also been improved by the addition of the MD data. The presentation of the cryo-EM is much better but in my opinion there are still conclusions drawn that are not supported by the data. These interpretations are generally supported by the biochemical and virological data, but that does not justify over-interpretation of the cryo-EM data and the authors should please be more cautious in what they write.

In particular:

The contact points between CPSF6 and CA tubes shown in Figure 5g are not the FG binding pockets (compare Fig 5g and Ext 11b). While it is perhaps reasonable to assume FG binding from the biochemical and virological data and the literature, there does not appear to be any cryo-EM evidence presented that FG repeats are bound into the cognate sites in the tubes. The authors have given possible reasons for this in the response to reviewers but they should probably state it in the manuscript and the inability to resolve FG is still surprising to me. The following statements therefore do not seem strictly correct: “CPSF6 contacts match the position of the hydrophobic pockets in pairs of adjoining CA hexamers”; “FG peptides bound to CA hexamer complexes were generated based on the X-ray crystal structure of the CA hexamer + IP6 + CPSF6313–327 complex (Extended Data Fig 12) and placed in the cognate hydrophobic CA pockets of specific CA monomers in adjoining CA hexamers to match densities observed in the cryo-EM maps (Fig 5g).”

In the absence of an observation of bound FG repeat, the structural data does not directly support the stoichiometry of FG binding. The low-resolution symmetrised density for CPSF6 and GST also does not provide convincing evidence for the overall stoichiometry of binding. In this context I don’t see how the stoichiometry of FG binding can be reliably deduced from the EM data. The following statement therefore seems to be incorrect: “In contrast to our X-ray crystal structure (Extended Data Fig 12a), which shows six CPSF6 FG peptides bound to six hydrophobic pockets in an isolated CA hexamer, our cryo-EM studies reveal the differential stoichiometry of CPSF6(LCR-FG-LCR) molecules binding to only two out of six cognate sites in the context of extended CA lattices (Fig 5g).”

Reviewer #3 (Remarks to the Author):

The authors have provided an excellent point by point rebuttal to each of the reviewers and have made a myriad of changes to the manuscript. The manuscript is much improved by these changes and the addition of substantial new data. The authors have addressed my concerns.

Point-by-point response to the reviewers' comments.

We appreciate additional constructive comments from Reviewer # 2 and have revised the manuscript accordingly. The revised text is in red.

Sincerely,

Mamuka Kvaratskhelia
Corresponding author

REVIEWER #2.

Comment: *The following statements therefore do not seem strictly correct: "CPSF6 contacts match the position of the hydrophobic pockets in pairs of adjoining CA hexamers";*

Response: The statement has been modified as follows (lines 200-203 on page 8): "CPSF6 contacts to adjoining CA hexamers most likely correspond to FG peptide binding to the cognate CA hydrophobic pockets seen in published[6,28] and our crystal structures of the CA hexamer + IP6 + $CPSF6_{313–327}$ peptide complex (Supplementary Fig 12, Supplementary Table 4)."

Comment: *"FG peptides bound to CA hexamer complexes were generated based on the X-ray crystal structure of the CA hexamer + IP6 + CPSF6313–327 complex (Extended Data Fig 12) and placed in the cognate hydrophobic CA pockets of specific CA monomers in adjoining CA hexamers to match densities observed in the cryo-EM maps (Fig 5g)."*

Response: The statement has been modified as follows (lines 247-249 on page 10): "FG peptides bound to CA hexamer complexes were generated based on the X-ray crystal structure of the CA hexamer + IP6 + $CPSF6_{313–327}$ complex (Supplementary Fig 12) and placed onto adjoining CA hexamers in accordance with the cryo-EM maps (Fig 5)."

Comment: *In the absence of an observation of bound FG repeat, the structural data does not directly support the stoichiometry of FG binding. The low-resolution symmetrised density for CPSF6 and GST also does not provide convincing evidence for the overall stoichiometry of binding. In this context I don't see how the stoichiometry of FG binding can be reliably deduced from the EM data. The following statement therefore seems to be incorrect: "In contrast to our X-ray crystal structure (Extended Data Fig 12a), which shows six CPSF6 FG peptides bound to six hydrophobic pockets in an isolated CA hexamer, our cryo-EM studies reveal the differential stoichiometry of CPSF6(LCR-FG-LCR) molecules binding to only two out of six cognate sites in the context of extended CA lattices (Fig 5g)."*

Response: The statement has been modified as follows (lines 387-391 on page 16):" The answer to this question is provided by our cryo-EM and biochemical studies (Fig 5 and Supplementary Fig 16). In contrast to our X-ray crystal structure (Supplementary Fig 12a), which shows six CPSF6 FG peptides bound to six hydrophobic pockets in an isolated CA hexamer, our cryo-EM studies reveal the differential, sub-stoichiometric binding of CPSF6(LCR-FG-LCR) molecules to extended CA lattices (Fig 5)."
